# Genome-wide analysis of NBS-LRR genes in Rosaceae species reveals distinct evolutionary patterns

**DOI:** 10.3389/fgene.2022.1052191

**Published:** 2022-11-10

**Authors:** Liping Guo, Chen You, Hanghang Zhang, Yukun Wang, Rui Zhang

**Affiliations:** ^1^ College of Horticulture, Northwest Agriculture and Forestry University, Yangling, China; ^2^ Guangdong Provincial Key Laboratory of Utilization and Conservation of Food and Medicinal Resources in Northern Region, Shaoguan University, Shaoguan, China; ^3^ Henry Fok School of Biology and Agriculture, Shaoguan University, Shaoguan, China

**Keywords:** Rosaceae, NBS-LRR, evolutionary patterns, phylogenetic analysis, gene duplication/loss

## Abstract

The nucleotide-binding site and leucine-rich repeat (*NBS-LRR*) genes, one of the largest gene families in plants, are evolving rapidly and playing a critical role in plant resistance to pathogens. In this study, a genome-wide search in 12 Rosaceae genomes screened out 2188 *NBS-LRR* genes, with the gene number varied distinctively across different species. The reconciled phylogeny revealed 102 ancestral genes (7 *RNLs*, 26 *TNLs*, and 69 *CNLs*), which underwent independent gene duplication and loss events during the divergence of the Rosaceae. The *NBS-LRR* genes exhibited dynamic and distinct evolutionary patterns in the 12 Rosaceae species due to independent gene duplication/loss events, which resulted the discrepancy of *NBS-LRR* gene number among Rosaceae species. Specifically, *Rubus occidentalis*, *Potentilla micrantha*, *Fragaria iinumae* and *Gillenia trifoliata*, displayed a “first expansion and then contraction” evolutionary pattern; *Rosa chinensis* exhibited a “continuous expansion” pattern; *F. vesca* had a “expansion followed by contraction, then a further expansion” pattern, three *Prunus* species and three *Maleae* species shared a “early sharp expanding to abrupt shrinking” pattern. Overall, this study elucidated the dynamic and complex evolutionary patterns of *NBS-LRR* genes in the 12 Rosaceae species, and could assist further investigation of mechanisms driving these evolutionary patterns.

## Introduction

Plants are threatened by diverse pathogens in the natural environment during their life cycle. To defend against invading pathogens, plants have evolved a specific immune system, in which the first line of defence response called PAMP- triggered immunity (PTI) and the second called effector-triggered immunity (ETI) (Dangl and Jones, 2001; Bhar et al., 2021). The disease *RESISTANCE* (*R*) gene can recognize invading pathogens and initiate the second line of defence (Jones and Dangl, 2006; Bhar et al., 2021). The nucleotide-binding site and leucine-reach repeat (*NBS-LRR*) genes constitute the largest class of *R* genes, and confer resistance to numerous plant pathogens (Meyers et al., 2005; Jones and Dangl, 2006; Zhou et al., 2020). A typical *NBS-LRR* gene comprises a variable N-terminal domain, a central nucleotide-binding site (NBS) domain containing several highly conserved and strictly ordered motifs, and a diverse C-terminal leucine-reach repeat (LRR) domain highly adaptable and involved in protein-protein interactions and pathogen recognition. *NBS-LRR* genes can be further classified into three subclasses, *TIR*-*NBS-LRR* (*TNL*), *CC*-*NBS-LRR* (*CNL*), and *RPW8*-*NBS-LRR* (*RNL*), based on whether their N-terminal region contains a Toll/interleukin-1 receptor (TIR) domain, a coiled-coil (CC) domain, or a resistance to powdery mildew 8 (RPW8) domain (Shao et al., 2014; Shao et al., 2016; Zhang et al., 2016; Qian et al., 2017; Shao et al., 2019).

Functional characterization revealed that *TNL* or *CNL* genes usually serve to trigger resistance pathways in plants by recognizing specific pathogens (Jones and Dangl, 2006; McHale et al., 2006; Zhou et al., 2020). Pathogens invasion recognized by the LRR domain of TNL or CNL proteins can elicits conformational changes in the NBS domain, and further cause multimerization of the TIR or CC domain and transmission of defense signals (Xue et al., 2019). By contrast, *RNL* genes do not function like regular *R* genes; they tend to function downstream and transduce signals from TNL or CNL proteins (Bonardi et al., 2011). *NBS-LRR* genes play essential roles in plant immunity during growth and development. For example, in *Arabidopsis thaliana*, the *TNL* gene *RPS4* confers specific resistance to a bacterial pathogen in an enhanced disease susceptibility 1 (*EDS1*) allele-dependent manner (Gassmann et al., 2010). A *CNL* resistance gene in cotton, *GbCNL130*, confers resistance to verticillium wilt across different hosts (Li et al., 2021). Likewise, the *CNL* gene *Pm21* (POWDERY MILDEW RESISTANCE21) confers broad-spectrum resistance to wheat powdery mildew disease (He et al., 2018); *Pi64*, which encodes a CNL protein, confers high-level and broad-spectrum resistance to leaf and neck blast in rice (Ma et al., 2015). *RppM*, encoding a typical CNL protein, confers resistance to southern corn rust in maize (Wang et al., 2022).

*NBS-LRR* genes originate in the green plant lineage (Shao et al., 2019), and have evolved into a large gene family in angiosperms *via* frequent recombination between paralogs, gene duplications/losses, and high substitution rate (Leister, 2004). The number of *NBS-LRR* genes differs markedly between species, owing to varying numbers of gene gain and loss events during evolution. Among Orchidaceae species, *Dendrobium catenatum* contains 115 *NBS-LRR* genes, while *Gastrodia elata* only harbors five *NBS-LRR* genes (Xue et al., 2019). In crop species, there are 129 and 508 *NBS-LRR* genes in maize and rice, respectively, representing a four-fold discrepancy (Li et al., 2010). With increasing availability of sequenced genomes for plants, genome-wide evolutionary analysis of *NBS-LRR* genes have been performed among many closely related species or subspecies to reveal diverse evolutionary patterns. For example, the *NBS-LRR* gene family in four Poaceae genomes, rice, maize, *Sorghum bicolor*, and *Brachypodium distachyon*, displays a “contracting” pattern (Li et al., 2010). In four Fabaceae species, *Medicago truncatula*, pigeon pea, common bean, and soybean, the *NBS-LRR* genes exhibit a “consistently expanding” pattern (Shao et al., 2014). Additionally, frequent lineage losses and deficient gene duplications dominate the evolution of *NBS-LRR* gene in three Cucurbitaceae genomes (cucumber, melon, and watermelon), resulting a low copy number in these species (Lin et al., 2013). Interestingly, *NBS-LRR* genes also display diverse evolutionary patterns in plants belonging to the same family. For example, in the Solanaceae, potato *BS-LRR* genes exhibit a “consistent expansion” pattern, tomato are characterized by a “expansion followed by contraction” pattern, while pepper display a “shrinking” pattern (Qian et al., 2017). In the Soapberry genomes, yellowhorn *NBS-LRR* genes exhibit an “expansion followed by contraction” pattern, while in both *Acer yangbiense* and longan, the gene evolutionary pattern is “expansion followed by contraction, and then further expansion” (Zhou et al., 2020). These results suggest that the evolutionary patterns of the *NBS-LRR* gene family are diverse in different plant species.

As one of the most economically important plant families, the Rosaceae consists of 90 genera with approximately 3,000 species distributed worldwide, including major fruits such as apple, strawberry, pear, peach, and sweet cherry, as well as ornamental flowers such as rose, rowan, and flowering peach (Longhi et al., 2014). These plants face constant threats from pathogens including bacteria, fungi, nematodes, and viruses, which results in seriously economic losses. In order to mitigate the threat of pathogens to Rosaceae plants, it is necessary to investigate *NBS-LRR* gene family members and understand their essential roles in different Rosaceae species. In recent years, with many Rosaceae family members having been sequenced, multiple genomes of this family are now available (https://www.rosaceae.org/). The systematic evaluation and comparison of *NBS-LRR* genes at the genome level in more Rosaceae species is needed to obtain a better understanding of the evolutionary history in this gene family. In the present study, 12 sequenced genome datasets from Rosaceae were subjected to genome-wide comparative analysis. The aim of this study was to unveil the evolutionary patterns of *NBS-LRR* genes in a wide range of Rosaceae species, and provide a foundation for further study of the mechanisms driving these evolutionary changes.

## Materials and methods

### Identification and classification of *NBS-LRR* genes

Whole genome sequences and annotation files of 12 Rosaceae species (*Fragaria vesca*, *Fragaria iinumae*, *Gillenia trifoliata*, *Prunus armeniaca*, *Prunus avium*, *Prunus persica*, *Potentilla micrantha*, *Pyrus betulifolia*, *Rosa chinensis*, *Rubus occidentalis*, *Malus baccata*, and *Malus* x *domestica*) were downloaded from the Genome Database for Rosaceae (https://www.rosaceae.org/; Supplementary File S1). BLAST and HMMER searches with the hidden Markov model of the NB-ARC domain (PF00931) as a query were simultaneously performed to identify the candidate *NBS-LRR* genes in the 12 Rosaceae genomes. The threshold expectation value was set at 1.0 for the BLAST search, and default parameters were used for the HMM search. All the obtained candidate genes were merged, and the redundant hits were removed manually. The remaining candidate genes then were subjected to online Pfam analysis (http://pfam.sanger. ac.uk/) and NCBI-CDD search to further corroborate the presence of N-terminal domain (CC (PF18052)/TIR (PF01582)/RPW8 (PF05659)) and NBS domains by an E-value of 10^−4^. Finally, the *NBS-LRR* genes were divided into *TNL*, *RNL*, and *CNL* classes based on their N-terminal domain.

### Analysis of *NBS-LRR* gene structures and conserved motif of NBS domain

To analyze the conserved motifs of NBS domain of TNL, RNL, and CNL proteins, the amino acid sequences of NBS domain were extracted and subsequently submitted to MEME online program (motifs should MEME find = 10) and WebLogo software (Crooks et al., 2004) with default parameters. Full-length coding sequences (CDS) and genomic DNA sequences of the *NBS-LRR* genes from 12 Rosaceae species were collected using TBtools (Chen et al., 2020) based on gff3 annotation files and submitted to GSDS2.0 (http://gsds.gao-lab.org/) to reveal the intron positions and phases among the three classes of *NBS-LRR* genes.

### Sequence alignment and phylogenetic analysis of *NBS-LRR* genes

The sequence alignment of the NBS domain and phylogenetic analysis were conducted as described by Zhang et al. (2016). The amino acid sequences of the NBS domain were firstly extracted by TBtools (Chen et al., 2020) and aligned using Muscle integrated into MEGA 5.0 with the default settings. The resultant amino acid sequence alignments were subsequently corrected and improved manually using MEGA 7.0. The maximum likelihood method phylogenetic tree of *NBS-LRR* genes were constructed based on the aligned amino acid sequences using JTT + F + R10 model of IQ-Tree (Nguyen et al., 2015). Branch support values were assessed using UFBoot2 tests, and the scale bar indicated the genetic distance (Minh et al., 2013). In addition, the gene duplication/loss events of *NBS-LRR* genes during the speciation of the 12 Rosaceae taxa were identified by reconciling the unrooted tree with the real species tree using Notung software (Chen et al., 2000).

### Synteny analysis within and across the rosaceae genomes

To identify collinear gene pairs and syntenic blocks within a genome or between different genomes, the pair-wise all-against-all blast of protein sequences was first conducted using the Diamond software. Then, the obtained results and gff3 annotation files were subjected to MCScanX (Wang et al., 2012) for intra- and interspecies microsynteny detection and gene duplication type determination. Finally, synteny relationship of *NBS-LRR* genes was displayed by TBtools (Chen et al., 2020). The ratio of nonsynonymous to synonymous nucleotide substitution (*Ka/Ks*) of gene pairs in each subclass were calculated according to Zhong et al. (2018).

## Results

### Identification of *NBS-LRR* genes in 12 rosaceae species

**FIGURE 1:**
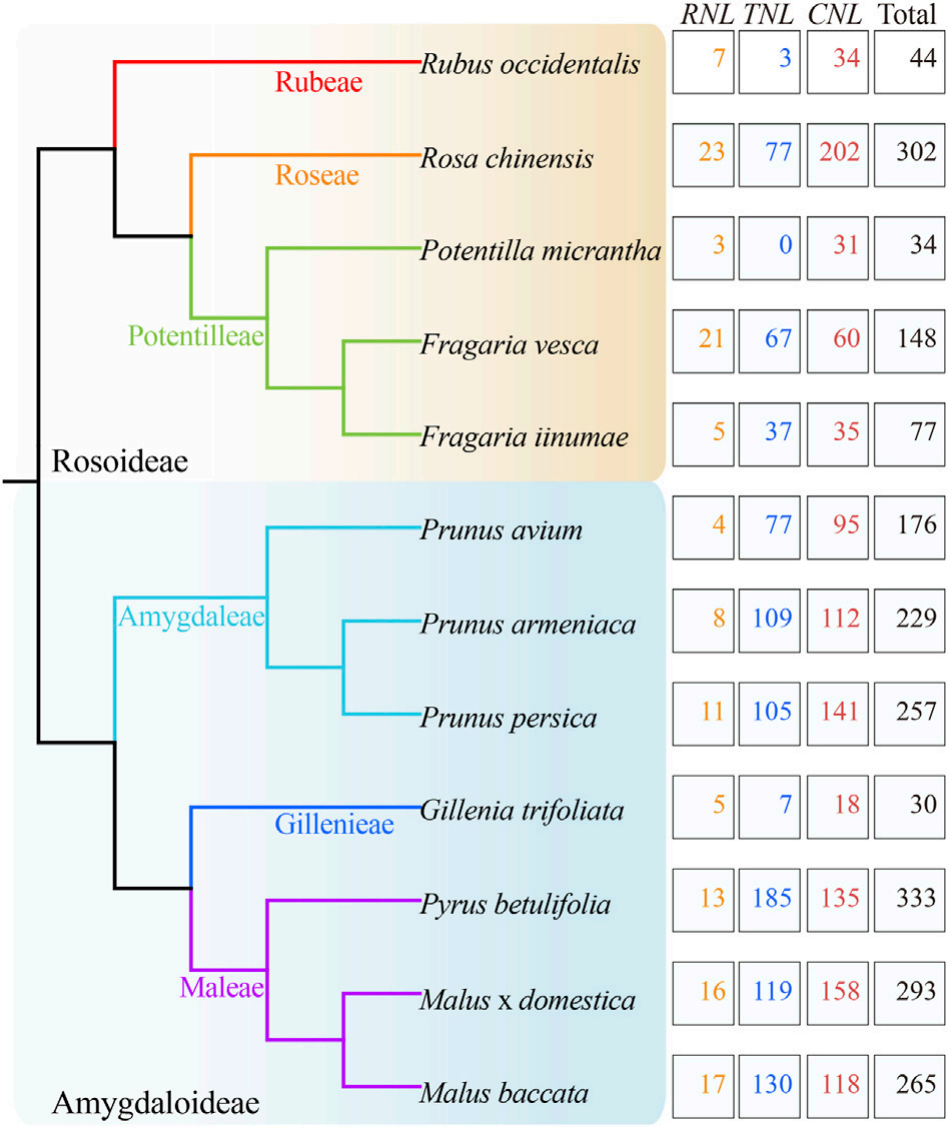
Identification and classification of *NBS-LRR* genes in 12 Rosaceae genomes. The phylogenetic tree (left) was constructed according to Xiang et al. (2017). The total number of *NBS-LRR* genes and their classification in each species (right) are shown.

After confirming the N-terminal CC/RPW8/TIR domains and the central NBS domain in protein sequences, 2188 *NBS-LRR* genes were identified from 12 Rosaceae genomes (Figure 1; Table 1; Supplementary Table S1). We found that the number of *NBS-LRR* genes varied considerably among different taxa. *Py. betulifolia* contained the largest number of *NBS-LRR* genes (333, 0.56% of all annotated genes). There were 302 (0.68%), 293 (0.65%), and 265 (0.58%) *NBS-LRR* genes in *Ro*. *chinensis*, *M. domestica* and *M. baccata* genomes, respectively. However, only 30 (0.10%), 34 (0.10%), and 44 (0.13%) *NBS-LRR* genes were identified in *G*. *trifoliata*, *Po*. *micrantha*, and *Ru. occidentalis* genomes, respectively (Table 1). We also found that the number of *NBS-LRR* genes differed markedly even among closely related species. For example, in the closely related species, *F. iinumae* and *F. vesca*, 77 and 148 *NBS-LRR* genes were found, respectively (Figure 1; Table 1). These results suggest that species-specific expansion or contraction of the *NBS-LRR* gene family has occurred in 12 Rosaceae species.

**TABLE 1:** The number of *NBS-LRR* genes identified in 12 Rosaceae genomes.

Domain compositions	*F. iinumae*	*F. vesca*	*G. trifoliata*	*M. baccata*	*M. domestica*	*Po. micrantha*	*Pr.armeniaca*	*Pr. avium*	*Pr. persica*	*Py. betulifolia*	*Ro. chinensis*	*Ru. occidentalis*
*RNL* Subclass	5 (6.49%)	21 (14.19%)	5 (16.67%)	17 (6.42%)	16 (5.46%)	3 (8.82%)	8 (3.49%)	4 (2.27%)	11 (4.28%)	13 (3.9%)	23 (7.62%)	7 (15.91%)
*RNL* (intact)	0	16	1	1	0	0	3	0	2	0	5	3
RN	5	5	4	16	16	3	5	4	9	13	18	4
*TNL* Subclass	37 (48.05%)	67 (45.27%)	7 (23.33%)	130 (49.06%)	119 (40.61%)	0 (0.00%)	109 (47.6%)	77 (43.75%)	105 (40.86%)	185 (55.56%)	77 (25.50%)	3 (6.82%)
*TNL* (intact)	5	58	0	7	10	0	6	5	4	12	4	0
*TN*	32	9	7	123	109	0	103	72	101	173	73	3
*CNL* Subclass	35 (45.45%)	60 (40.54%)	18 (60.00%)	118 (44.53%)	158 (53.92%)	31 (91.18%)	112 (48.91%)	95 (53.98%)	141 (54.86%)	135 (40.54%)	202 (66.89%)	34 (77.27%)
*CNL* (intact)	5	49	11	39	58	18	42	35	65	46	93	14
*CN*	30	11	7	79	100	13	70	60	76	89	109	20
Total number	77	148	30	265	293	34	229	176	257	333	302	44
Proportion of Intact genes	12.99%	83.11%	40.00%	17.74%	23.21%	52.94%	22.27%	22.73%	27.63%	17.42%	33.77%	38.64%
Proportion to total protein-coding genes	0.33%	0.52%	0.10%	0.58%	0.65%	0.10%	0.75%	0.40%	0.96%	0.56%	0.68%	0.13%
Average gene size (bp)	6,169.52	5,332.29	4,321.77	6,363.63	4,807.84	4332.558,824	8,221.33	5,027.25	4,914.43	7,161.54	4,107.38	6,584.16
Genome size (Mb)	270.00	240.00	320.00	778.00	742.00	406	230.00	353.00	265.00	511.00	560.00	293.00
Average length of coding-sequence (bp)	3,346.73	3,757.21	3,030.60	3,060.12	3,379.34	2,844.97	3,510.07	2,818.08	3,113.59	3,394.83	2,885.40	3,012.43

Based on the conserved domains (CC/RPW8/TIR) in the N-terminal region, the *NBS-LRR* genes in the 12 Rosaceous species were classified into three subclasses (Figure 1; Table 1). There were 1139, 916, and 133 members in *CNL*, *TNL*, and *RNL* subclasses, respectively. The *CNL* genes were detected in all 12 Rosaceae species, while the *TNL* genes was absent in *Po. micrantha*. *RNL* genes were also found in all Rosaceae species, albeit with lower numbers than those of *CNL* and *TNL* genes. In the 12 Rosaceae species, only 617 *NBS-LRR* genes were structurally complete containing all three types of domains (CC/RPW8/TIR-NBS-LRR), and the rest 1571 genes lacked the LRR domain (Table 1). More interestingly, the *F. vesca* had the highest proportion of intact *NBS-LRR* genes (83.11%), while the *F. iinumae* comprised the fewest intact *NBS-LRR* genes (12.99%). Theoretically, *NBS-LRR* genes should have an intact structure to trigger immune responses and transmit defense signals, but several studies indicated that genes without intact structures may also function in plant immunity (Nandety et al., 2013; Kato et al., 2014).

### Conserved motifs of the NBS domain in rosaceae

**FIGURE 2:**
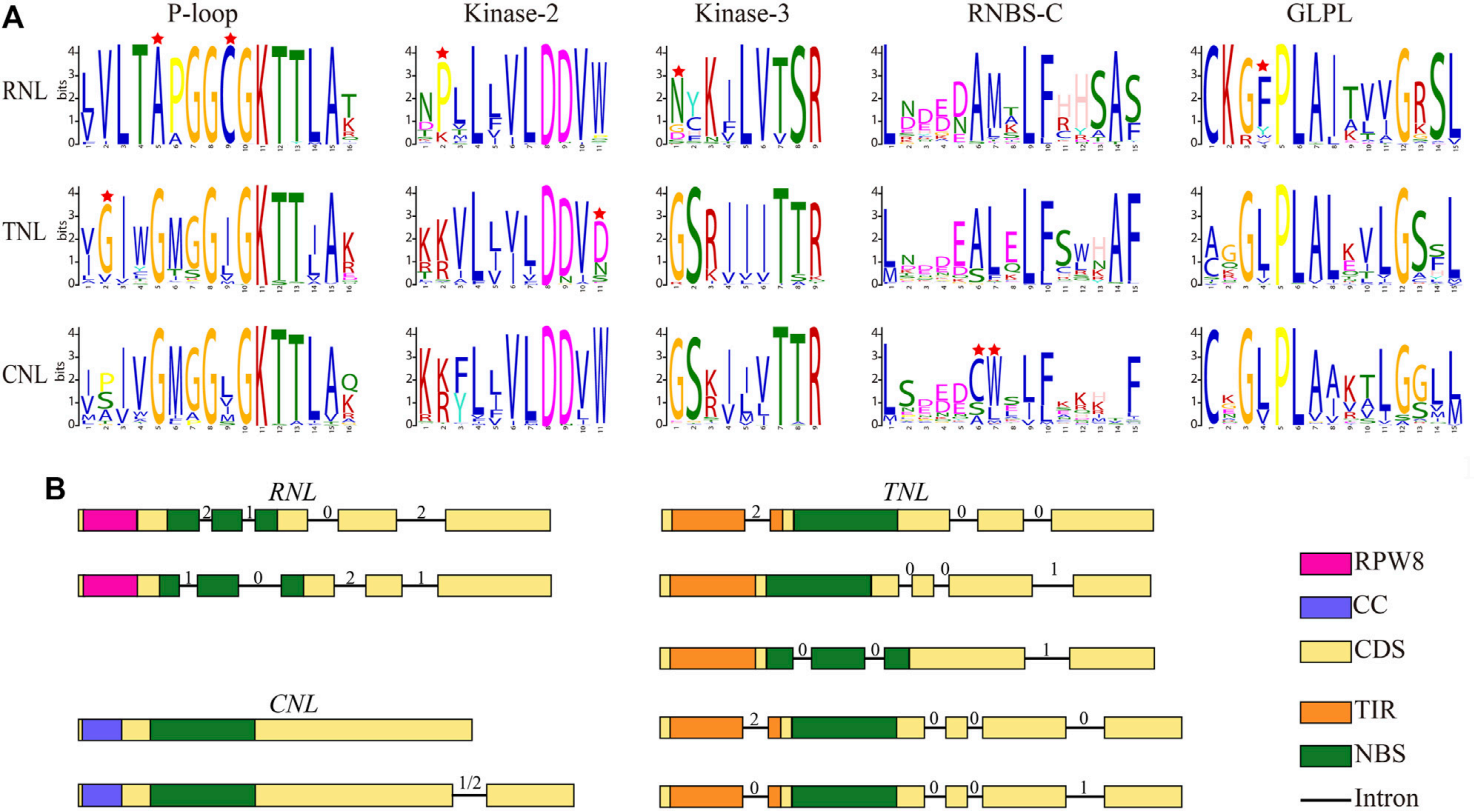
Conserved motifs and exon–intron structure models for the three *NBS-LRR* gene subclasses in Rosaceae genomes. **(A)** Amino acids of five conserved motifs. Conserved amino acids among *CNL*, *TNL*, and *RNL* subclass genes are labeled with a red star. **(B)** Exon-intron structure. Filled boxes represent exons and lines represent introns. The numbers on top represent the intron phase.

It has been shown that the NBS domain is composed of several functionally conservative motifs, which are always strictly ordered (DeYoung and Innes, 2006; Yue et al., 2016). We analyzed the conserved motifs in the NBS domain of *NBS-LRR* genes identified in this study. Five conserved motifs, P-loop, Kinase-2, Kinase-3, RNBS-C, and GLPL, were identified from the N-terminal to C-terminal regions of NBS domain (Figure 2A). These motifs displayed different degree of similarities among *RNL*, *TNL*, and *CNL* subclasses. P-loop and Kinase-2 motifs showed not only high similarities among the three subclasses but also high conservations within each subclass, compared with other motifs. However, the Kinase-3 motif showed distinctive differences among the three subclasses, but was conserved within each subclass. Notably, RNBS-C and GLPL motifs exhibit remarkable differences both among the three subclasses and within each subclass. In addition, unique amino acid sites could be used as preliminary labels to characterize subclass members without phylogenetic analysis (Figure 2A). For example, the fifth site alanine (A) in the P-loop motif, the second site proline (P) in the Kinase-2 motif, the first site asparagine (N) in the Kinase-3 motif, and the fourth site phenylalanine (F) in the GLPL motif could serve as preliminary labels to categorize *RNL* subclass members. For *TNL* subclass members, the second site glycin (G) in the P-loop motif and the 11th site aspartic acid (D) could be used as preliminary labels. Moreover, the sixth site cysteine (C) and seventh site tryptophan (W) in the RNBS-C motif could be used to identify *CNL* subclass members.

### Diversity of *NBS-LRR* gene structure

Furthermore, we analyzed structural features of *NBS-LRR* genes identified in this study, including the number of introns, intron position, and intron phase (excluding 34 *Po. micrantha NBS-LRR* genes because the *Po*. *micrantha* genome was only assembled at the contig level). The number of introns was distinctively different in *NBS-LRR* genes of 11 Rosaceae species, ranging from 0 to 31, and the average number of introns in *RNL*, *TNL*, and *CNL* subclass members was 4.70, 4.76, and 1.43, respectively (Supplementary Table S2). In the *RNL* subclass, the majority of members had four introns, with the first two introns separating the NBS domain (Figure 2B). There were two types of intron phases in the *RNL* subclass: 2, 1, 0, 2 and 1, 0, 2, 1. In the *CNL* subclass, 438 members had no intron, while 339 members harbored 1 N-terminal intron and the intron phase was 1 or 2 (Figure 2B). In the *TNL* subclass, a total of 163, 283, and 172 members contained 3, 4, or 5 introns, respectively. Most 3-intron *TNL* subclass members had three types of intron phase: 2, 0, 0 with the first intron separating the TIR domain; 0, 0, 1 with three N-terminal introns; 0, 0, 1 with the first two introns separating the NBS domain. The majority of the 4-intron *TNL* subclass members had two types of intron phase (2, 0, 0, 0 and 0, 0, 0, 1), with the first intron separating the TIR domain (Figure 2B). These results indicate that *NBS-LRR* genes in Rosaceae species have subclass-specific characteristics that can be used to categorize *CNL*, *TNL*, and *RNL* subclass members.

### Phylogenetic analysis of *NBS-LRR* genes

**FIGURE 3:**
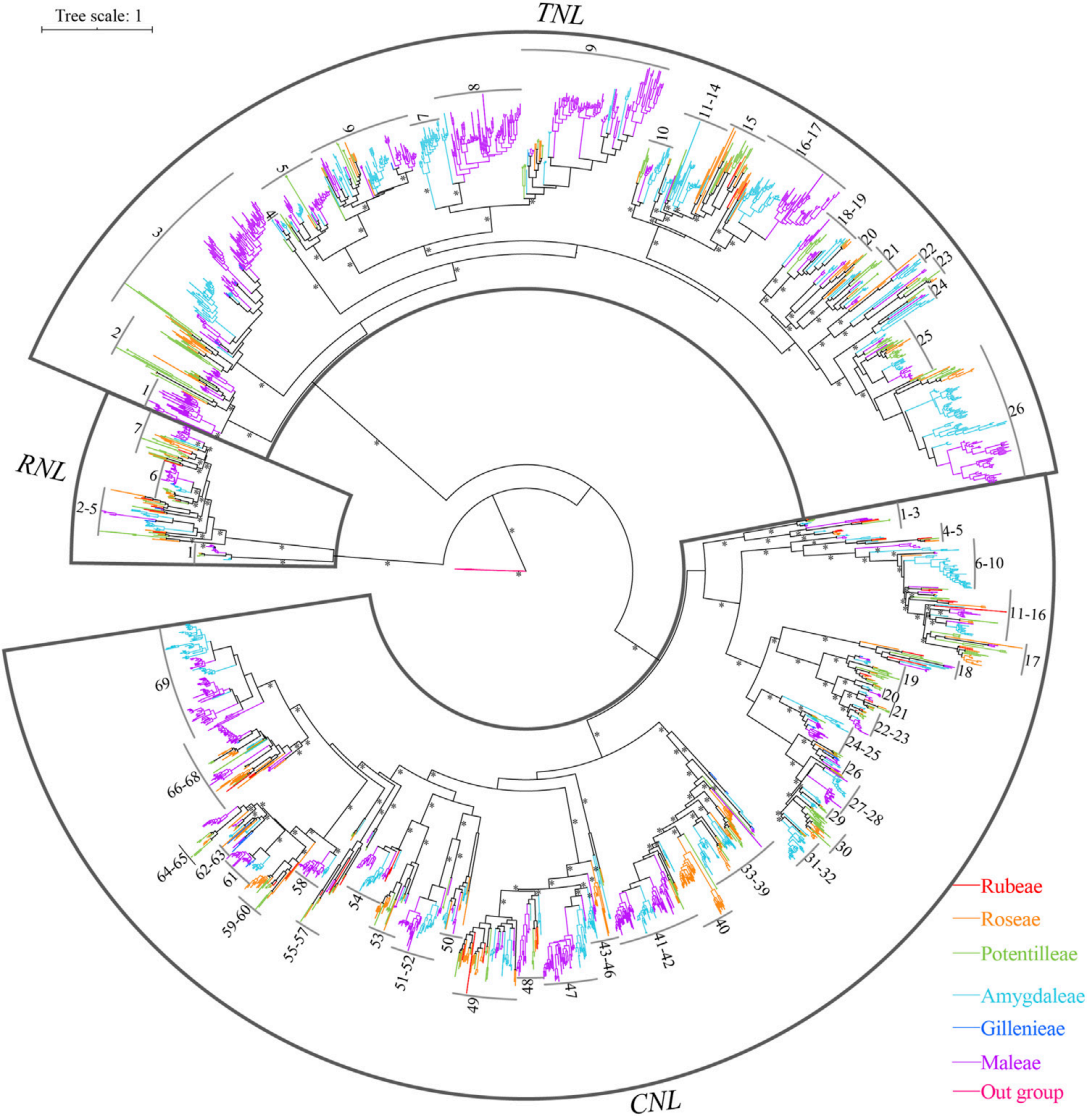
Phylogenetic relationships of *NBS-LRR* genes from Rosaceae genomes. *NBS-LRR* genes from distinctive plant tribes are indicated by different colors. Branch support values > 70% for basal nodes are indicated by an asterisk (*). The detailed phylogenetic trees are shown in Supplementary Figures S1, S2.

To explore the phylogenetic relationships and evolutionary history of *NBS-LRR* genes, phylogenetic analysis was carried out with the basal angiosperm *Amborellla trichopoda NBS* as outgroups. We found that three clades exactly represented the divergence of *RNLs*, *TNLs*, and *CNLs* (Supplementary Figure S1; Figure 3). All *NBS-LRR* genes fell into groups of corresponding subclasses without exception. By comparing the lengths of the three branches, it was possible to estimate that the *RNL* subclass had the lowest evolutionary rate than the others.

In addition, the reconstructed *NBS-LRR* gene phylogeny revealed 102 ancestral lineages of *NBS-LRR* genes, including 7 *RNL* lineages, 26 *TNL* lineages, and 69 *CNL* lineages (Figure 3; Supplementary Figure S2; Supplementary Table S3). Not all 12 Rosaceae species retained to the 102 ancestral lineages in their genomes. For example, *Pr. armeniaca* kept 67 lineages (3 *RNLs*, 21 *TNLs*, and 43 *CNLs*); *Pr*. *persica* maintained 65 lineages (4 *RNLs*, 20 *TNLs*, and 41 *CNLs*); *Ro*. *chinensis* had 56 lineages (7 *RNLs*, 18 *TNLs*, and 41 *CNLs*). By contrast, fewer *NBS-LRR* ancestral lineages were retained by *G*. *trifoliate* (4 *RNLs*, 4 *TNLs*, and 12 *CNLs*), *Po*. *micrantha* (3 *RNLs* and 19 *CNLs*), and *Ru*. *occidentalis* (4 *RNLs*, 3 *TNLs*, and 19 *CNLs*). Despite this, none of these 102 ancestral lineages was retained by all the 12 species. Lineages 1 and 6 of *RNLs*, as well as lineages 18, 21, and 68 of *CNLs*, were reserved in 11 of the 12 investigated species, indicating their evolutionary conservativeness; while lineages 9, 44, and 59 of *CNLs* were only found in one species (*Ro. chinensis*), indicating their evolutionary uniqueness.

**FIGURE 4:**
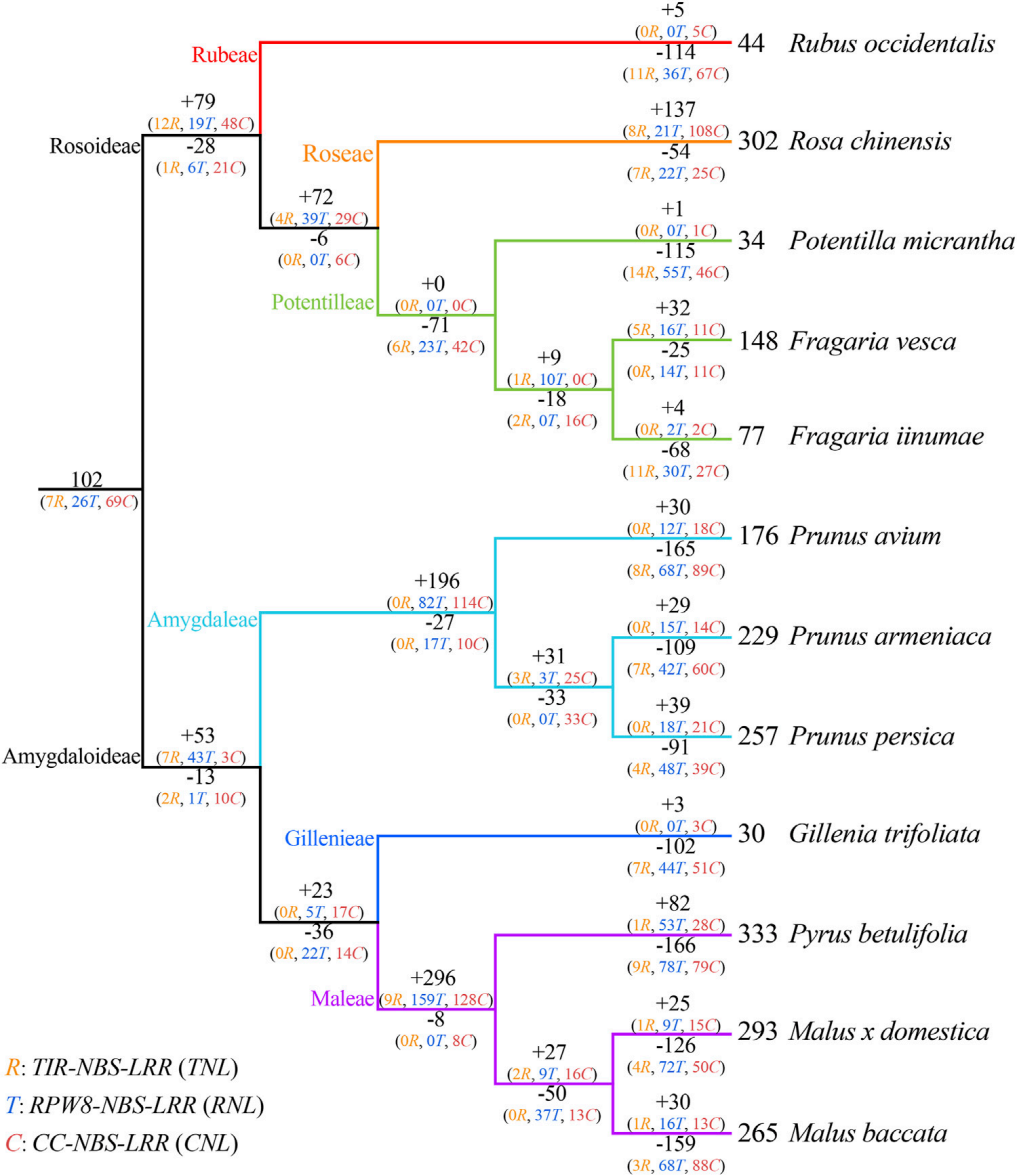
Duplication and loss events of *NBS-LRR* genes during Rosaceae evolution. Gene gains and losses are indicated by + or—symbols on each branch. Detailed information for gene gain and loss events is shown in Supplementary Figure S2.

We also observed species-specific expansion of *NBS-LRR* genes in the Rosaceae (Figure 4; Supplementary Figure S2; Supplementary Table S3). For example, the species-specific gene duplications have expanded the gene numbers of lineages 40, 49, 60, and 68 of the *CNLs*, and lineage 15 of the *TNLs* in *Ro*. c*hinensis*, and lineages 7 of the *RNLs* in *F. vesca.* The species-specific expansion was also found in lineages 3 and 9 of the *TNL*s, and lineage 69 of the *CNL*s in *Py. betulifolia*. In the case of *M*. *domestica*, species-specific gene expansion was found in lineages 3, 8, and 9 of the *TNL*, and lineages 42 and 69 of the *CNL* subclass. As for *M*. *baccata*, there was species-specific gene expansion in lineages 3, 8, and 9 of the *TNL*, and lineage 69 of the *CNL*. These species-specific gene duplications have expanded the *NBS-LRR* numbers of 12 Rosaceae species. The previous research also found that the species-specific duplications has mainly contributed the recent expansion of NBS-LRR genes in five Rosaceae species (Zhong et al., 2015). In addition, independent gene loss events were evident in the evolutionary history of *NBS-LRR* genes in the Rosaceae, and none of the species maintained all 102 ancestral lineages.

### Evolutionary patterns of *NBS-LRR* genes

To investigate the evolutionary patterns of *NBS-LRR* genes in the 12 Rosaceae species, the phylogenetic tree was reconciled with the species tree to recovered gene loss and duplication events that occurred during species speciation (Figure 4; Supplementary Figure S2; Supplementary Table S4). The number of common ancestors increased from 102 to 153 (+79/−28) before differentiation of the Rosoideae subfamily. The evolutionary pattern of *NBS-LRR* genes *in Ru*. *occidentalis* which diverged first, displayed an “expansion followed by contraction” evolutionary pattern, with gene losses outpacing gene duplications, resulting in only 44 *NBS-LRR* genes in its genome. The evolutionary pattern of *NBS-LRR* genes in *Ro. chinensis* was “continuous expansion”, and recent species-specific duplications contributed substantially to the number of *NBS-LRR* genes in the genome. *Po*. *micrantha* and *F. iinumae* shared a similar “first expansion and then constant contraction” pattern. In *Po*. *micrantha*, a large number of genes were lost recently, leaving in only 34 *NBS-LRR* genes in the current genome. In *F. vesca*, the evolutionary pattern was “expansion followed by contraction, then a further expansion”, resulting in more *NBS-LRR* genes than in the two closely related species *Po. micrantha* and *F. iinumae*.

The number of *NBS-LRR* genes increased from 102 to 142 (+53/−13) before the divergence of seven species in Amygdaloideae. Subsequently, *NBS-LRR* genes in the *Prunus* genus firstly underwent a sharp gene duplication (+196/−27), leading to an increase in the number of *NBS-LRR* genes to 311 in their common ancestor. Subsequently, all three species in the *Prunus* genus experienced recent abrupt gene losses, resulting in a shrinking in the number of *NBS-LRR* genes. Therefore, the *NBS-LRR* genes in the three *Prunus* species underwent an “early sharp expanding to abrupt shrinking” pattern. In addition, three Maleae species, *Py*. *betulifolia*, *M*. *baccata*, and *M*. *domestica*, shared a similar evolutionary patterns to those of *Prunus* species. The large number of gene duplications (+296/−8) experienced by the common ancestor of Maleae contributed to the large number of *NBS-LRR* genes, while the recent gene losses was responsible for the differences in *NBS-LRR* gene number among the three species. *G. trifoliata* exhibited a “first expansion and then contraction”pattern, and should have experienced more severe gene losses than gene duplications, resulting in only 30 *NBS-LRR* genes in its genome. These results suggest that gene duplication/loss events are the main factor explaining the distinctive differences of *NBS-LRR* gene number among Rosaceae species, and that *NBS-LRR* genes in the 12 Rosaceae species underwent dynamic and complex evolutionary processes.

### Syntenic relationship of *NBS-LRR* genes in rosaceae genomes

**FIGURE 5:**
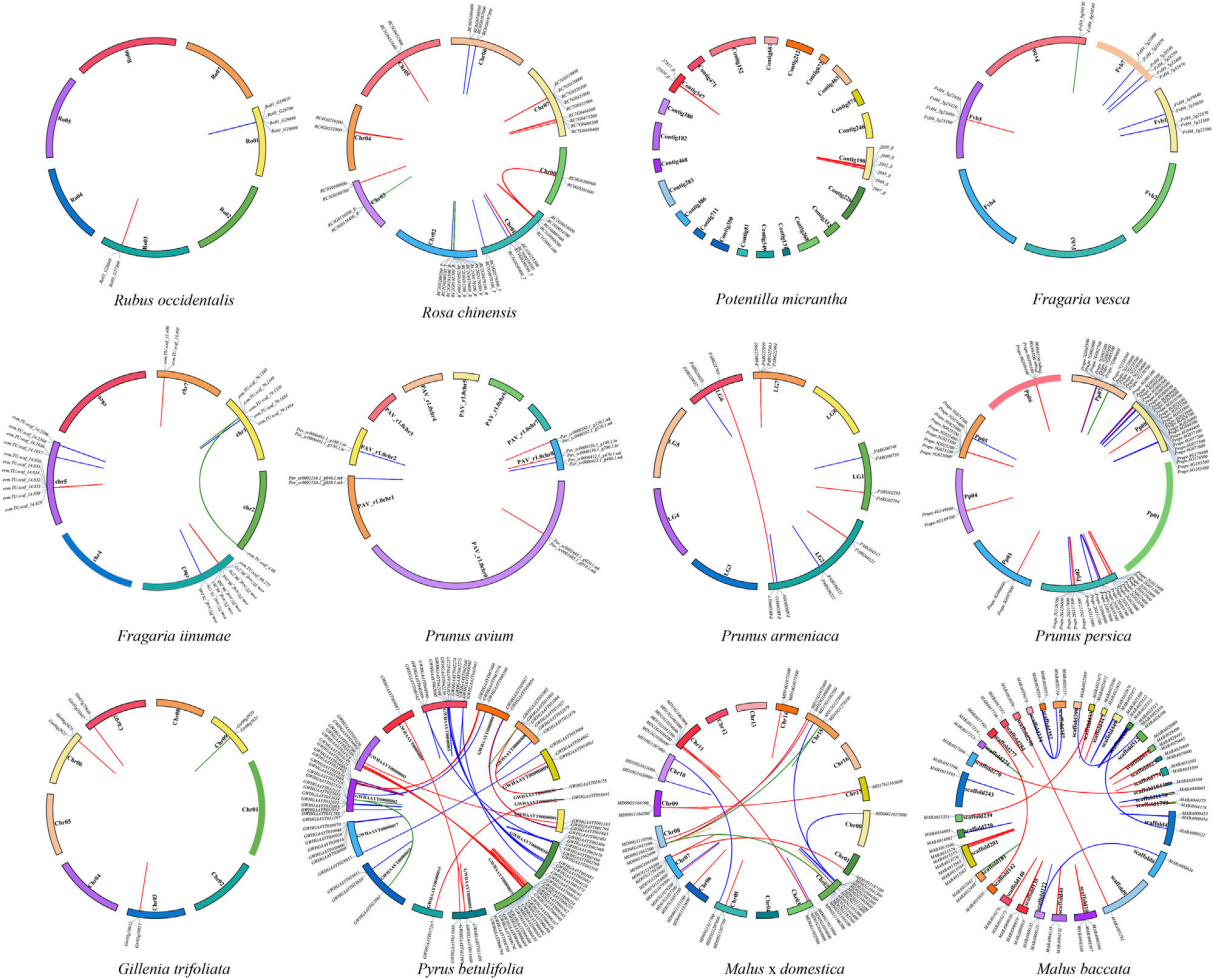
Localization and synteny of *NBS-LRR* genes in Rosaceae genomes. *NBS-LRR* genes in 12 Rosaceae species were mapped to different chromosomes/contigs/scaffolds. Gene pairs of *RNLs*, *TNLs*, and *CNLs* with a syntenic relationship are connected with green, blue and red lines, respectively.

Intra-genomic synteny analysis was performed to uncover whether the *NBS-LRR* genes were derived from whole genome duplications (duplication of genes *via* an increase in ploidy), segmental duplications (copying of entire blocks of genes from one chromosome to another), tandem duplications (duplication of a gene *via* unequal crossing over between similar alleles) or ectopic duplications (duplication of individual or small groups of genes to an unlinked locus) (Leister, 2004; Panchy et al., 2016). And 243 gene pairs (i.e., 31 *RNL* pairs, 83 *TNL* pairs, and 129 *CNL* pairs) were identified (Figure 5; Table 2). The number of tandem duplication events of *NBS-LRR* genes was 183, considerably greater than the number of segmental duplication events (60 in total). The number of tandem duplications was higher in 11 of the 12 investigated species, with one exception of *Ro. chinensis*, in which 27 segmental duplication events and 26 tandem duplication events were identified. Interestingly, there were no segmental duplication events in *F*. *vesca*, *G*. *trifoliata*, and *Ru*. *occidentalis*. These results indicate that tandem duplication might be the main type of *NBS-LRR* gene duplications in the Rosaceae. Nevertheless, we speculate that other types of duplication may have occurred because the number of *NBS-LRR* genes in both *F*. *vesca* and *Pr*. *armeniaca* was higher than the number of *NBS-LRR* ancestral lineages, while only nine duplication events were identified.

**TABLE 2:** Gene duplication types for *NBS-LRR* genes in 12 Rosaceae species.

Species	*RNL*		*TNL*		*CNL*		Total segmental duplications	Total tandem duplications	Total gene pairs in each species
	Segmental	Tandem	Segmental	Tandem	Segmental	Tandem			
*F. iinumae*	1	2	0	4	0	10	1	16	17
*F. vesca*	0	1	0	6	0	2	0	9	9
*G. trifoliata*	0	1	0	0	0	3	0	4	4
*M. baccata*	0	2	5	8	4	20	9	30	39
*M. domestica*	4	3	2	5	6	10	12	18	30
*Po. micrantha*	0	0	0	0	0	4	0	4	4
*Pr. armeniaca*	0	0	0	4	1	4	1	8	9
*Pr. avium*	0	0	0	3	0	3	0	6	6
*Pr. persica*	0	4	2	14	0	23	2	41	43
*Py. betulifolia*	1	3	13	12	13	11	27	26	53
*Ro. chinensis*	0	6	0	5	8	6	8	17	25
*Ru. occidentalis*	0	3	0	0	0	1	0	4	4
Total number	31		83		129		60	183	243

**FIGURE 6:**
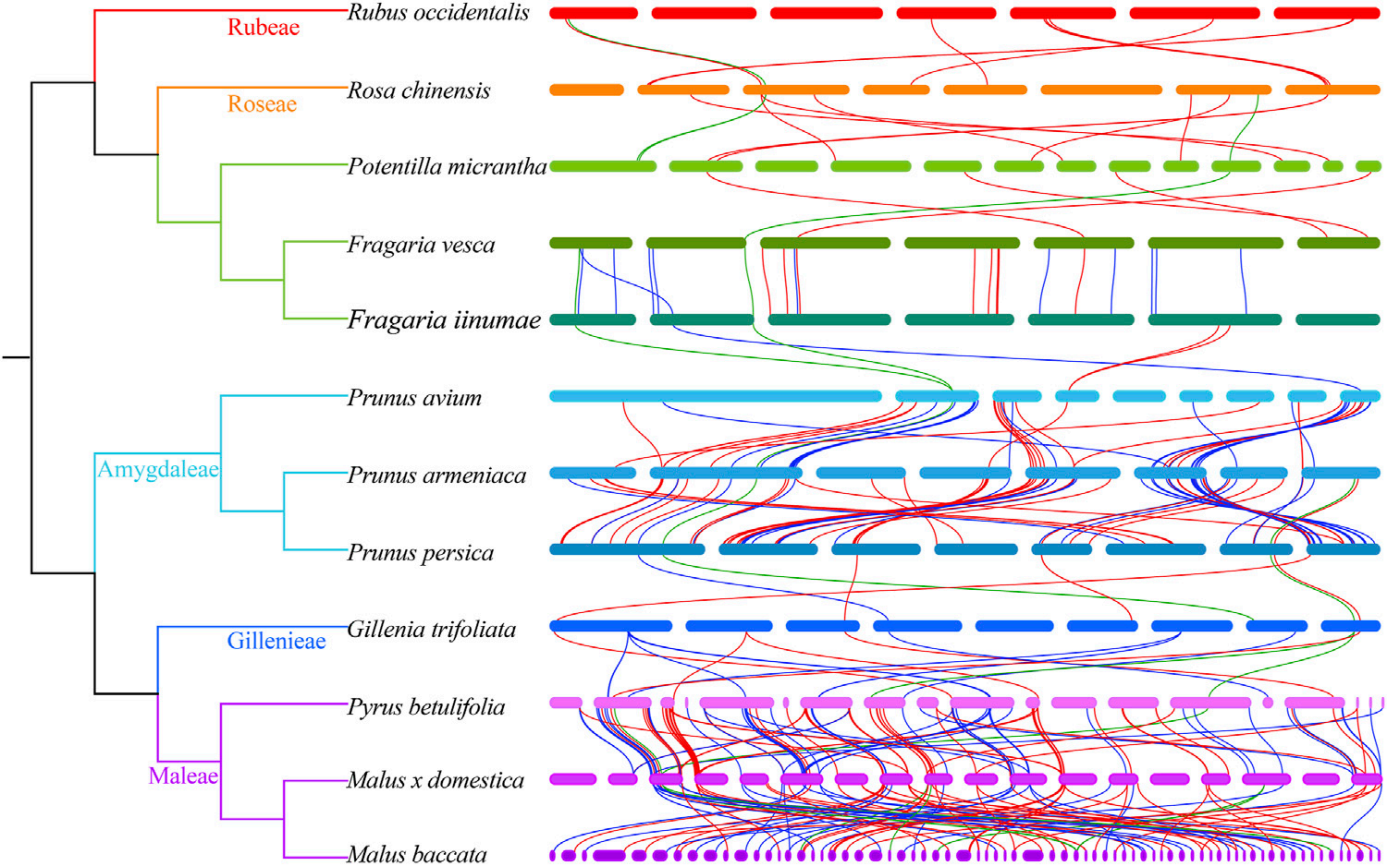
Collinearity of *NBS-LRR* genes between closely related Rosaceae species. The green, blue and red lines show the collinear gene pairs of *RNLs*, *TNLs* and *CNLs* between 12 Rosaceae species.

Based on the results of evolutionary analysis, we also investigated collinear relationships of *NBS-LRR* genes between closely related Rosaceae species, and identified 425 syntenic gene pairs (i.e., 28 *RNL* pairs, 182 *TNL* pairs, and 215 *CNL* pairs; Figure 6 and Supplementary Table S5). Notably, a large number of syntenic gene pairs was found among Maleae and *Prunus* species. For example, there were 117 collinear gene pairs between *Py*. *betulifolia* and *M*. *domestica*, 94 between *M*. *domestica* and *M*. *baccata*, and 85 between *Pr*. *armeniaca* and *Pr*. *avium*. Furthermore, we found that the number of *NBS-LRR* genes and their genetic relationship might influence the number of syntenic gene pairs between closely related species. For instance, 22 syntenic gene pairs existed between *F*. *iinumae* and *F*. *vesca*, but only five pairs were found between *F*. *iinumae* and *Pr*. *avium*.

**FIGURE 7:**
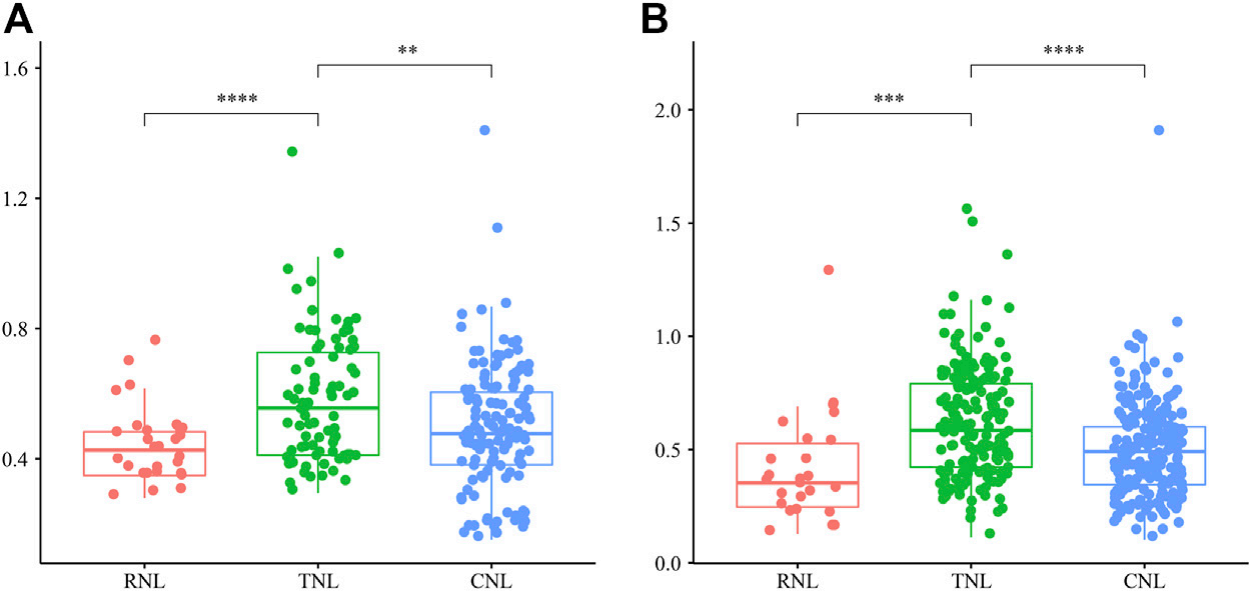
The *Ka/Ks* ratios of *NBS-LRR* gene pairs in 12 Rosaceae genomes. **(A)** The *Ka/Ks* ratios of intraspecies *NBS-LRR* pairs in three subclasses. **(B)** The *Ka/Ks* ratios of interspecies *NBS-LRR* pairs in three subclasses. In the boxplot, the middle line indicates the median, the box indicates the range of the 25 to 75th percentiles of the total data, the whiskers extend to data points less than 1.5 × IQR (interquartile range) away from the 1st/3rd quartile.

To detect the direction and intensity of selection, we calculated the *Ka/Ks* ratios of genes pairs in each subclass. As shown in Figure 7, most of the gene pairs of each subclass had had *Ka/Ks* values less than 1, which indicated that which indicated that most *NBS-LRR* genes were under purifying selection in the 12 Rosaceae species. However, 4 paralogs (2 *TNLs* and 2 *CNLs*) and 12 orthologs (1 *RNL*, 9 *TNLs* and 2 *CNLs*) had *Ka/Ks* ratios greater than 1, respectively, illustrating that these *NBS*-*LRR* genes were driven by positive selection. The *Ka/Ks* ratios showed highly significant difference among the three subclasses, with *TNL* genes having significantly greater average *Ka/Ks* values than those of *RNLs* and *CNLs*, demonstrating that the *TNLs* are subject to stronger diversifying selection and a faster evolutionary rate than the others.

## Discussion

### Differences in the number of *NBS-LRR* genes in the rosaceae genomes

Previous studies have found that the number of *NBS-LRR* genes differs considerably between plant species, even in genetically close species or subspecies. For example, a study of *NBS-LRR* genes in 22 angiosperm genomes revealed prominent differences among species, with 571 *NBS-LRR* genes being identified in the *M. truncatula* genome, while only 88 in the *Thellungiella salsuginea* genome (Shao et al., 2016). In potato, a Solanaceae species, *NBS-LRR* genes were 1.75-fold more than those in the closely related tomato and pepper (Qian et al., 2017). Moreover, the number of *NBS-LRR* genes in maize was shown to be only a fourth of that in rice (Li et al., 2010). Studies on *Oryza*, *Glycine* and *Gossypium* also showed large differences in the number of *NBS-LRR* genes, not only among species, but also within species (Zhang et al., 2010). In the present study, we also found extreme differences in the number of *NBS-LRR* genes among the 12 Rosaceae genomes. For example, there are more than 300 *NBS-LRR* genes in each of *Ro. chinensis* and *Py. betulifolia* genomes, but only 30, 34, and 44 *NBS-LRR* genes in *G. trifoliate*, *Po. micrantha*, and *Ru. occidentalis* genomes, respectively (Figure 1; Table 1). It is noteworthy that the number of *NBS-LRR* genes in each species in the present study may be the lowest compared with previous studies. For example, Zhong et al. (2015) and Jia et al. (2015) identified 354 and 437 *NBS*-*LRR* genes in peaches, respectively, while we retained only 144 genes. Arya at al. (2014) and Zhong et al. (2015) reported 748 and 1015 *NBS*-*LRR* genes in apple, respectively, while we only preserved 293 for analysis. The two main reasons for this difference may be that we used different datasets in different researchers, and the other is that we only retained those genes with both N-terminal domain and NBS domain, while the previous study retained the genes containing only NBS or LRR domains. Several factors, such as genome size, natural selection, distribution range, and artificial selection, can influence the number of members in a plant gene family (Zhang et al., 2010). However, we found no specific relationship between the number of *NBS-L*RR genes and genome size in 12 Rosaceae species (Table 1). Rosaceae species with large genomes do not necessarily contain a large number of *NBS-LRR* genes. For example, *Po. micrantha* contains fewer *NBS-LRR* genes than *F. vesca*, *Pr. armeniaca*, and *Pr. persica*, despite having the largest genome (Table 1). Similar results have been reported in several plant families. In Fabaceae, the genome size of soybean is twice that of *M*. *truncatula*, but soybean contains fewer *NBS-LRR* genes than *M*. *truncatula* (Shao et al., 2014). Also, there is no relationship between the number of *NBS-LRR* genes and genome size in three Sapindaceae species (Zhou et al., 2020). In addition, the number of *NBS-LRR* genes in gramineous plants is not related to the number of protein-encoding genes (Li et al., 2010). In line with this finding, we found no correlation between the number of *NBS-LRR* genes and the number of protein-coding genes in the 12 Rosaceae species (Table 1).

Gene duplications and losses can cause gene expansion and contraction, thereby resulting in differences in the number of *NBS-LRR* genes among different species or subspecies. In Sapindaceae, the *NBS-LRR* genes of longan, yellowhorn, and *A. yangbiense* underwent different recent gene duplication/loss events, resulting in clear differences in the number of *NBS-LRR* genes in these three species (Zhou et al., 2020). Moreover, gene duplication/loss-induced differences in the number of *NBS-LRR* gene among species have been reported for Cucurbitaceae, Leguminous, Nightshade, Brassica, Gramineae, and Orchidaceae plants (Li et al., 2010; Lin et al., 2013; Shao et al., 2014; Li et al., 2016; Qian et al., 2017; Xue et al., 2020). Our results also indicate that species-specific gene duplication/loss events occurred in *NBS-LRR* gene evolution after diverging from the common ancestor. Consequently, the number of *NBS-LRR* genes differed between different species, even closely related species. For example, the number of *NBS-LRR* genes in *Ro. chinensis* increased rapidly due to recent species-specific gene duplication. On the contrary, the number of *NBS-LRR* genes in *Ru. occidentalis* is quite low due to abrupt gene loss events (Figure 1).

Four types of gene duplications, tandem duplications, ectopic duplications, whole-genome duplications, and segmental duplications, have been reported in *NBS-LRR* gene evolution (Leister, 2004). In Fabaceae, Brassicaceae, and Solanaceae, most *NBS-LRR* gene duplications are tandem duplications (Shao et al., 2014; Zhang et al., 2016; Qian et al., 2017). In *D. catenatum* and *Phalaenopsis equestris*, tandem and ectopic duplications are the main causes of *NBS-LRR* gene expansion (Xue et al., 2020). In current study, both tandem duplications and segmental duplications facilitated diversity and evolution of *NBS-LRR* genes Rosaceae species, with tandem duplications as the main factor. Our result was consistent with the previous study which also claimed tandem duplication played a major role in *NBS*-encoding gene expansion in the four Rosaceae species (Jia et al., 2015).

Accordingly, the differences in the number of *NBS-LRR* genes in the Rosaceae species might be related to distributions of each species in natural and cultivated systems. Rosaceae species possessing a large number of *NBS-LRR* genes are always distributed widely, and might have undergone frequent natural or artificial selection, resulting in high resistance to pathogens. For instance, *F. iinumae* is found only in northwestern Japan, and has fewer *NBS-LRR* genes presumably due to the relatively stable environment and pathogen diversity. Different from *F. iinumae*, *F. vesca* is mainly distributed in Europe, Asia, and North America. The wide planting area, complex growth environment, and multiple pathogens increase selection pressure for survival; therefore, more *NBS-LRR* genes are present in the *F. vesca* genome. Similarly, the number of *NBS-LRR* genes is also high in Maleae, *Prunus*, and *Rosa* species due to the extensive planting range (Figure 1; Table 1).

### Evolutionary patterns of NBS-LRR genes in rosaceae species

*NBS-LRR* genes in angiosperms have evolved quickly in dynamic and diverse patterns. In *M. truncatula*, pigeon pea, common bean, and soybean, the evolutionary pattern of *NBS-LRR* genes is characterized by “consistent species-specific gene duplication” (Shao et al., 2014); in *T. salsuginea*, *Capsella rubella*, *Brassica rapa*, *Arabidopsis lyrate*, and *A. thaliana*, the pattern is “expansion first and then contraction” (Zhang et al., 2016). Even in closely related species, *NBS-LRR* genes underwent different evolutionary processes. For instance, *NBS-LRR* genes in yellowhorn underwent “expansion followed by contraction”, but the pattern was “expansion followed by contraction and a further expansion” for *A. yangbiense* and longan (Zhou et al., 2020). Using evolutionary analysis, the *NBS-LRR* ancestral lineages in 12 Rosaceae species were reconstructed in the present study, and their dynamic evolutionary patterns during species differentiation were traced. A total of 102 *NBS-LRR* ancestors were recovered, and they underwent species-specific evolution during divergence (Figure 4; Supplementary Figure S2; Supplementary Table S4).

Similar to Poaceae, Brassicaceae, and Sapindaceae plants (Li et al., 2010; Zhang et al., 2016; Zhou et al., 2020), the *NBS-LRR* genes in the Rosaceae displayed diverse evolutionary patterns (Figure 4; Supplementary Figure S2; Supplementary Table S4). For example, the evolutionary pattern of *NBS-LRR* genes in *Ru. occidentalis*, *Po*. *micrantha*, *F*. *iinumae*, and *Pr*. *avium* was “first expanded then contracted,” while that in *Ro*. *chinensis* was “continuous expansion.” Three *Prunus* species and three *Maleae* species shared an “early sharp expanding to abrupt shrinking” pattern. This evidence suggests that complex evolutionary patterns are the main reason for the differences in the number of *NBS-LRR* genes in surveyed Rosaceae plants.

### Factors responsible for differences between *NBS-LRR* subclasses

*NBS-LRR* genes can be divided into *CNL*, *TNL*, and *RNL* subclasses (Shao et al., 2016; Zhang et al., 2016; Qian et al., 2017; Shao et al., 2019). In many plants, there are more *CNL*s than *TNLs* and *RNLs*. Shao et al. reported more *CNLs* than the other two subclasses in 19 of 22 angiosperm species (Shao et al., 2016). In addition, the proportion of *CNL* genes in the *NBS-LRR* gene family can be >90% in *Phyllostachys heterocycle*, *B. distachyon*, pepper, rice, maize, *S. bicolor*, millet (*Setaria italic*), banana (*Musa acuminate*), tomato, and sesame (*Sesamum indicum*) (Shao et al., 2016). In the present study, we found a differing number of *CNLs*, *TNLs*, and *RNLs* in Rosaceae genomes, with *CNLs* being the most prevalent (1139) and *RNLs* being the scarcest (133) (Figure 1 and Table 1).

There are two possible reasons for the differences in the number of *NBS-LRR* genes between the three subclasses, namely, the differing number of ancestral genes, and the complex pattern of genetic evolution among different subclasses. In five Brassicaceae plants (*T. salsuginea*, *C. rubella*, *B. rapa*, *A. lyrate*, and *A. thaliana*), 148 *TNL*, 70*CNL*, and 10 *RNL* ancestors eventually expanded into 478 *TNL*, 272*CNL*, and 32*RNL* genes (Zhang et al., 2016). In three Sapindaceae genomes (yellowhorn, longan, and *A*. *yangbiense*), the number of *CNL* ancestors is much greater than those of *TNL* ancestors and *RNL* ancestors, resulting in a much higher number of *CNL* genesthan *TNL* genes and *RNL* genes in extant species (Zhou et al., 2020). In three Solanaceae crop species (potato, tomato, and pepper), the number of *CNL* ancestors is also much greater than those of *RNL* ancestors and *TNL* ancestors, leading to a much higher number of *CNL* genes than *TNL* genes and *RNL* genes in extant species (Qian et al., 2017). In legumes, *CNLs* and *TNLs* have the same number of ancestral genes, but the number of *CNL* genes is higher than that of *TNL* genes due to the *CNL* subclass showing a higher gene expansion rate (Shao et al., 2014).

In the present study, the number of *CNL*, *RNL*, and *TNL* subclass genes also differed significantly. Based on phylogenetic analysis, common ancestors of *NBS-LRR* genes (7 *RNL* ancestors, 26 *TNL* ancestors, and 69 *CNL* ancestors) were identified in 12 Rosaceae species (Figure 3; Supplementary Figure S2). The ancestors of each subclass underwent complex evolutionary patterns in each species. Except for *Ru. occidentalis* and *Po. micrantha*, the number of *RNL* genes is considerably lower than that of *CNL* and *TNL* genes in Rosaceae species, mainly because of the much lower number of *RNL* ancestors than that of *CNL* and *TNL* ancestors. The *TNL* lineage may have been lost in *Po. micrantha* during the recent divergence process (Figure 1; Table 1). In addition, the number of *CNL* and *TNL* genes is not remarkably different between the two *Fragaria* species (i.e., *F. iinumae* and *F. vesca*), which might be because *CNL* genes underwent more gene loss events and *TNL* genes underwent more gene duplication events. A similar situation occurred in *Pr. avium*, *Pr. armeniaca*, *Py. betulifolia*, and *M. baccata*, resulting in more *TNL* genes than *CNL* genes, or no clear differences (Figure 4; Supplementary Figure S2; Supplementary Table S4). Overall, the discrepancies in the number of *CNL*, *RNL*, and *TNL* subclass genes in Rosaceae species are mainly attributed to prominent differences in the number of ancestral genes and gene duplication/loss events.

In conclusion, genome-wide comparative analysis of *NBS-LRR* genes of 12 Rosaceae species were performed. In total, 2188 genes were identified, with the number varied considerably among different taxa. The *NBS-LRR* genes in the 12 Rosaceous species were classified into three subclasses, with the number of *CNLs* being the highest and *RNLs* the lowest. A total of 102 ancestral genes were reconciled in the common ancestor of the 12 Rosaceae species. The analysis of gene duplication/loss events revealed the *NBS-LRR* genes of 12 Rosaceae species exhibited dynamic and distinct evolutionary patterns in the 12 Rosaceae species.

## Data Availability

The original contributions presented in the study are included in the article/Supplementary Material, further inquiries can be directed to the corresponding authors.
