# Supplementary material for: Genome-wide analysis of NBS-LRR genes in Rosaceae species reveals distinct evolutionary patterns
Source: Front Genet. 2022 Nov 10;13:1052191. doi: 10.3389/fgene.2022.1052191 (PMC9685399; doi:10.3389/fgene.2022.1052191)
Supplement: Supplementary file 4 [file DataSheet3.PDF]

the Genome Database for Rosaceae： https://www.rosaceae.org/
Fragaria vesca: https://www.rosaceae.org/species/fragaria_vesca/genome_v4.0.a1
Fragaria iinumae: https://www.rosaceae.org/species/fragaria_iinumae/genome_v1.0
Malus x domestica: https://www.rosaceae.org/species/malus/malus_x_domestica/genome_GDDH13_v1.1
Malus baccata: https://www.rosaceae.org/species/malus_baccata/genome_v1.0
Prunus armeniaca: https://www.rosaceae.org/species/prunus_armeniaca/genome_v1.0
Prunus persica: https://www.rosaceae.org/species/prunus_persica/genome_v2.0.a1
Prunus avium: https://www.rosaceae.org/species/prunus_avium/genome_v1.0.a1
Pyrus betulifolia: https://www.rosaceae.org/species/pyrus_betulifolia/genome_v1.0
Rosa chinensis: https://www.rosaceae.org/species/rosa/chinensis/genome_v1.0
Gillenia trifoliata: https://www.rosaceae.org/Analysis/10816123
Rubus occidentalis: https://www.rosaceae.org/analysis/268
Potentilla micrantha: https://www.rosaceae.org/analysis/274
