# Supplementary material for: Genome-wide analysis of NBS-LRR genes in Rosaceae species reveals distinct evolutionary patterns
Source: Front Genet. 2022 Nov 10;13:1052191. doi: 10.3389/fgene.2022.1052191 (PMC9685399; doi:10.3389/fgene.2022.1052191)
Supplement: Supplementary file 5 [file DataSheet1.PDF]

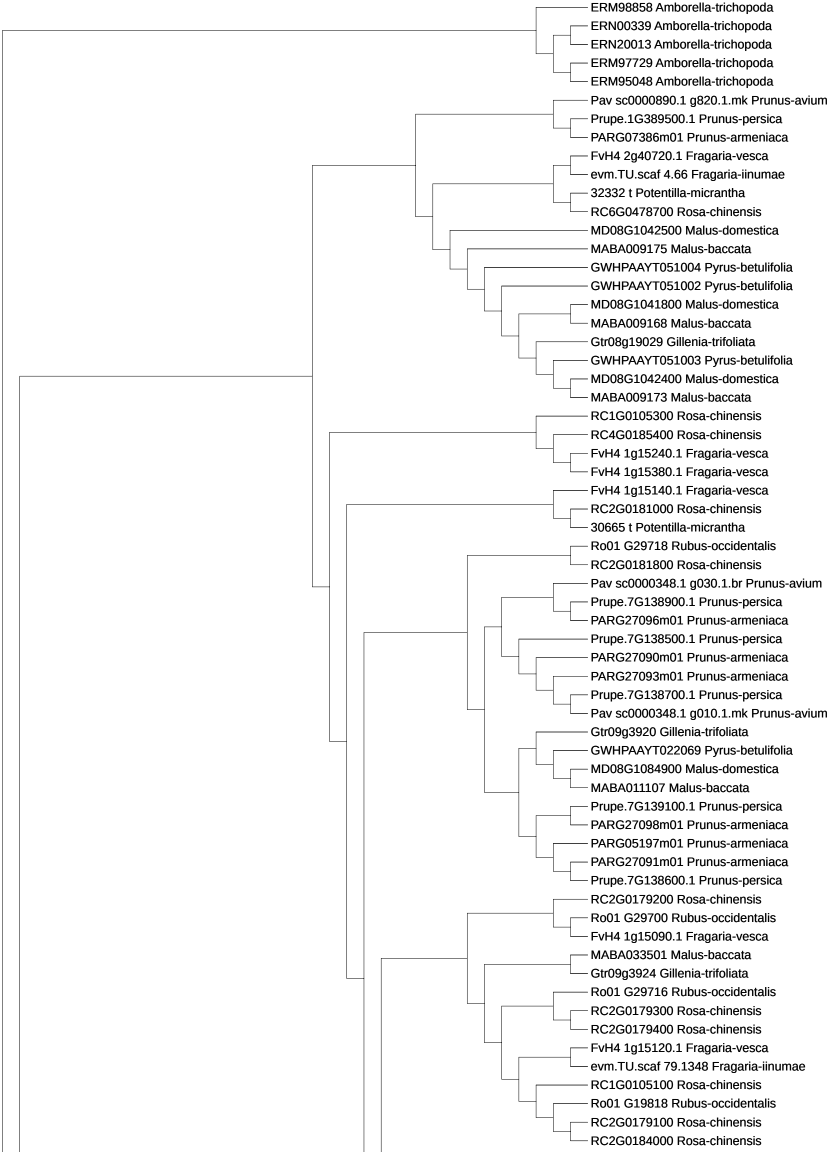
ERM98858 Amborella-trichopoda
ERN00339 Amborella-trichopoda
ERN20013 Amborella-trichopoda
ERM97729 Amborella-trichopoda
ERM95048 Amborella-trichopoda
Pav sc0000890.1 g820.1.mk Prunus-avium
Prupe.1G389500.1 Prunus-persica
PARG07386m01 Prunus-armeniaca
FvH4 2g40720.1 Fragaria-vesca
evm.TU.scaf 4.66 Fragaria-iinumae
32332 t Potentilla-micrantha
RC6G0478700 Rosa-chinensis
MD08G1042500 Malus-domestica
MABA009175 Malus-baccata
GWHPAAYT051004 Pyrus-betulifolia
GWHPAAYT051002 Pyrus-betulifolia
MD08G1041800 Malus-domestica
MABA009168 Malus-baccata
Gtr08g19029 Gillenia-trifoliata
GWHPAAYT051003 Pyrus-betulifolia
MD08G1042400 Malus-domestica
MABA009173 Malus-baccata
RC1G0105300 Rosa-chinensis
RC4G0185400 Rosa-chinensis
FvH4 1g15240.1 Fragaria-vesca
FvH4 1g15380.1 Fragaria-vesca
FvH4 1g15140.1 Fragaria-vesca
RC2G0181000 Rosa-chinensis
30665 t Potentilla-micrantha
Ro01 G29718 Rubus-occidentalis
RC2G0181800 Rosa-chinensis
Pav sc0000348.1 g030.1.br Prunus-avium
Prupe.7G138900.1 Prunus-persica
PARG27096m01 Prunus-armeniaca
Prupe.7G138500.1 Prunus-persica
PARG27090m01 Prunus-armeniaca
PARG27093m01 Prunus-armeniaca
Prupe.7G138700.1 Prunus-persica
Pav sc0000348.1 g010.1.mk Prunus-avium
Gtr09g3920 Gillenia-trifoliata
GWHPAAYT022069 Pyrus-betulifolia
MD08G1084900 Malus-domestica
MABA011107 Malus-baccata
Prupe.7G139100.1 Prunus-persica
PARG27098m01 Prunus-armeniaca
PARG05197m01 Prunus-armeniaca
PARG27091m01 Prunus-armeniaca
Prupe.7G138600.1 Prunus-persica
RC2G0179200 Rosa-chinensis
Ro01 G29700 Rubus-occidentalis
FvH4 1g15090.1 Fragaria-vesca
MABA033501 Malus-baccata
Gtr09g3924 Gillenia-trifoliata
Ro01 G29716 Rubus-occidentalis
RC2G0179300 Rosa-chinensis
RC2G0179400 Rosa-chinensis
FvH4 1g15120.1 Fragaria-vesca
evm.TU.scaf 79.1348 Fragaria-iinumae
RC1G0105100 Rosa-chinensis
Ro01 G19818 Rubus-occidentalis
RC2G0179100 Rosa-chinensis
RC2G0184000 Rosa-chinensis

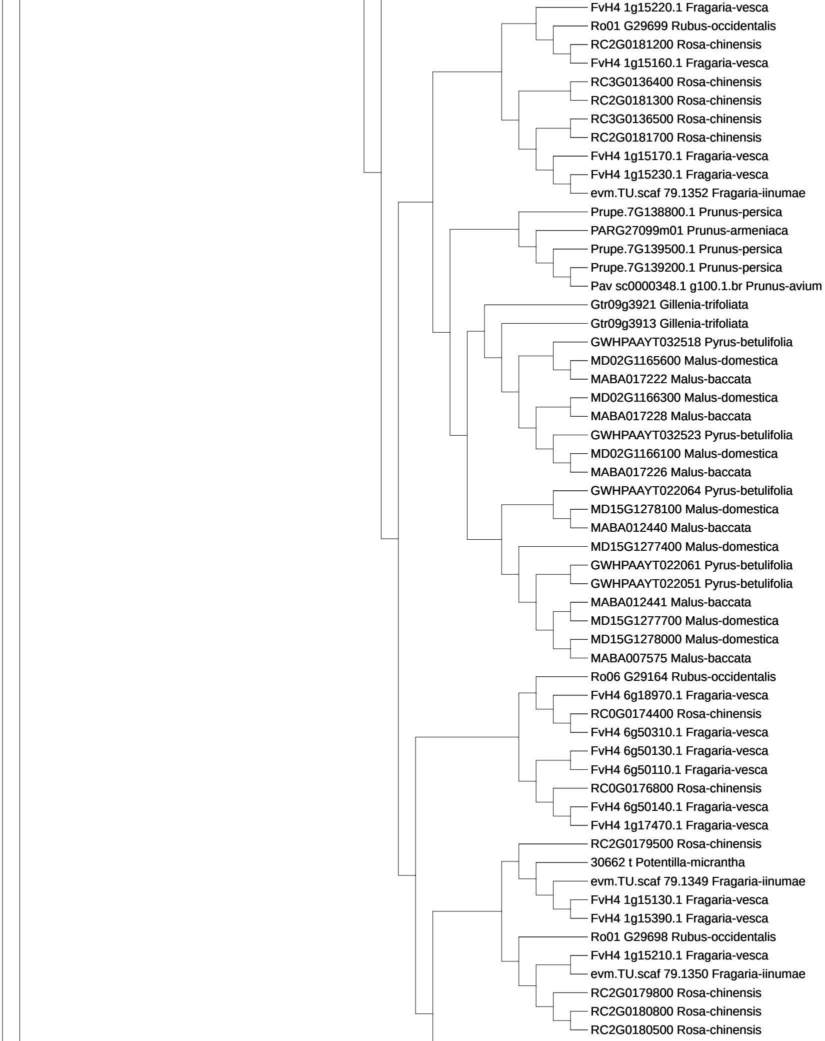
FvH4 1g15220.1 Fragaria-vesca
Ro01 G29699 Rubus-occidentalis
RC2G0181200 Rosa-chinensis
FvH4 1g15160.1 Fragaria-vesca
RC3G0136400 Rosa-chinensis
RC2G0181300 Rosa-chinensis
RC3G0136500 Rosa-chinensis
RC2G0181700 Rosa-chinensis
FvH4 1g15170.1 Fragaria-vesca
FvH4 1g15230.1 Fragaria-vesca
evm.TU.scaf 79.1352 Fragaria-iinumae
Prupe.7G138800.1 Prunus-persica
PARG27099m01 Prunus-armeniaca
Prupe.7G139500.1 Prunus-persica
Prupe.7G139200.1 Prunus-persica
Pav sc0000348.1 g100.1.br Prunus-avium
Gtr09g3921 Gillenia-trifoliata
Gtr09g3913 Gillenia-trifoliata
GWHPAAYT032518 Pyrus-betulifolia
MD02G1165600 Malus-domestica
MABA017222 Malus-baccata
MD02G1166300 Malus-domestica
MABA017228 Malus-baccata
GWHPAAYT032523 Pyrus-betulifolia
MD02G1166100 Malus-domestica
MABA017226 Malus-baccata
GWHPAAYT022064 Pyrus-betulifolia
MD15G1278100 Malus-domestica
MABA012440 Malus-baccata
MD15G1277400 Malus-domestica
GWHPAAYT022061 Pyrus-betulifolia
GWHPAAYT022051 Pyrus-betulifolia
MABA012441 Malus-baccata
MD15G1277700 Malus-domestica
MD15G1278000 Malus-domestica
MABA007575 Malus-baccata
Ro06 G29164 Rubus-occidentalis
FvH4 6g18970.1 Fragaria-vesca
RC0G0174400 Rosa-chinensis
FvH4 6g50310.1 Fragaria-vesca
FvH4 6g50130.1 Fragaria-vesca
FvH4 6g50110.1 Fragaria-vesca
RC0G0176800 Rosa-chinensis
FvH4 6g50140.1 Fragaria-vesca
FvH4 1g17470.1 Fragaria-vesca
RC2G0179500 Rosa-chinensis
30662 t Potentilla-micrantha
evm.TU.scaf 79.1349 Fragaria-iinumae
FvH4 1g15130.1 Fragaria-vesca
FvH4 1g15390.1 Fragaria-vesca
Ro01 G29698 Rubus-occidentalis
FvH4 1g15210.1 Fragaria-vesca
evm.TU.scaf 79.1350 Fragaria-iinumae
RC2G0179800 Rosa-chinensis
RC2G0180800 Rosa-chinensis
RC2G0180500 Rosa-chinensis

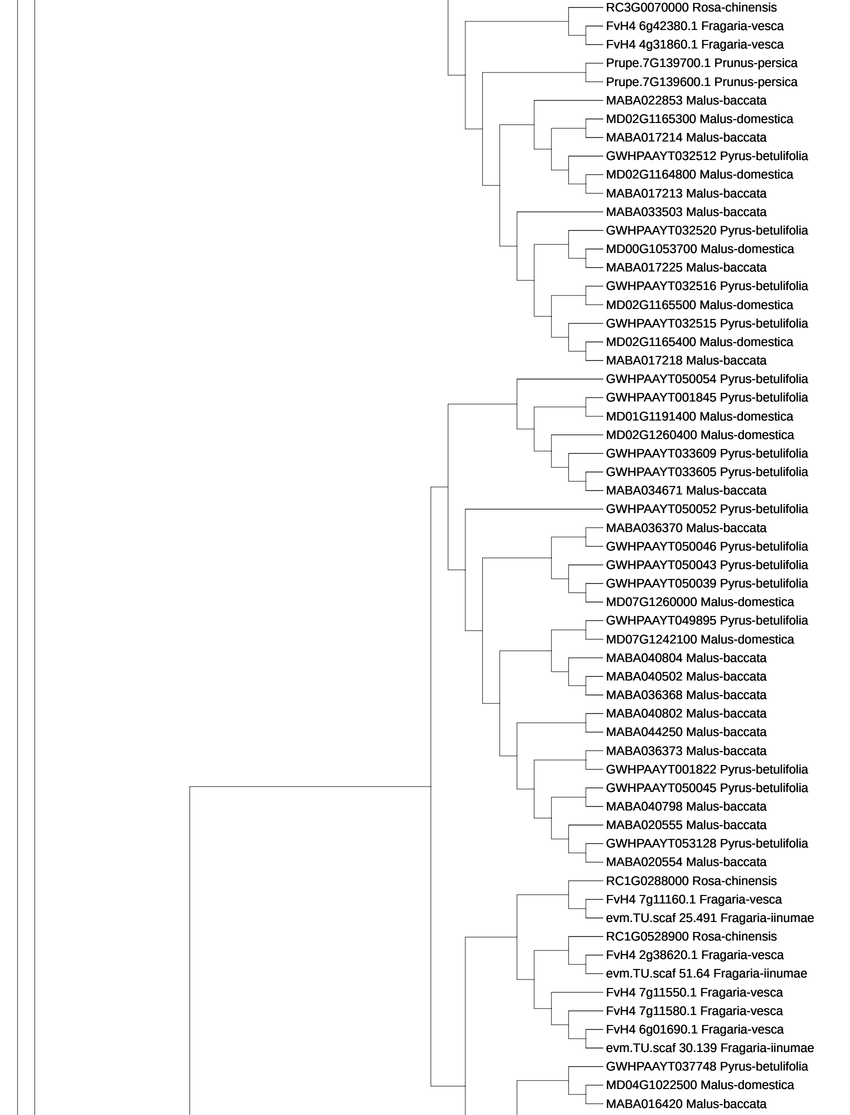
RC3G0070000 Rosa-chinensis
FvH4 6g42380.1 Fragaria-vesca
FvH4 4g31860.1 Fragaria-vesca
Prupe.7G139700.1 Prunus-persica
Prupe.7G139600.1 Prunus-persica
MABA022853 Malus-baccata
MD02G1165300 Malus-domestica
MABA017214 Malus-baccata
GWHPAAYT032512 Pyrus-betulifolia
MD02G1164800 Malus-domestica
MABA017213 Malus-baccata
MABA033503 Malus-baccata
GWHPAAYT032520 Pyrus-betulifolia
MD00G1053700 Malus-domestica
MABA017225 Malus-baccata
GWHPAAYT032516 Pyrus-betulifolia
MD02G1165500 Malus-domestica
GWHPAAYT032515 Pyrus-betulifolia
MD02G1165400 Malus-domestica
MABA017218 Malus-baccata
GWHPAAYT050054 Pyrus-betulifolia
GWHPAAYT001845 Pyrus-betulifolia
MD01G1191400 Malus-domestica
MD02G1260400 Malus-domestica
GWHPAAYT033609 Pyrus-betulifolia
GWHPAAYT033605 Pyrus-betulifolia
MABA034671 Malus-baccata
GWHPAAYT050052 Pyrus-betulifolia
MABA036370 Malus-baccata
GWHPAAYT050046 Pyrus-betulifolia
GWHPAAYT050043 Pyrus-betulifolia
GWHPAAYT050039 Pyrus-betulifolia
MD07G1260000 Malus-domestica
GWHPAAYT049895 Pyrus-betulifolia
MD07G1242100 Malus-domestica
MABA040804 Malus-baccata
MABA040502 Malus-baccata
MABA036368 Malus-baccata
MABA040802 Malus-baccata
MABA044250 Malus-baccata
MABA036373 Malus-baccata
GWHPAAYT001822 Pyrus-betulifolia
GWHPAAYT050045 Pyrus-betulifolia
MABA040798 Malus-baccata
MABA020555 Malus-baccata
GWHPAAYT053128 Pyrus-betulifolia
MABA020554 Malus-baccata
RC1G0288000 Rosa-chinensis
FvH4 7g11160.1 Fragaria-vesca
evm.TU.scaf 25.491 Fragaria-iinumae
RC1G0528900 Rosa-chinensis
FvH4 2g38620.1 Fragaria-vesca
evm.TU.scaf 51.64 Fragaria-iinumae
FvH4 7g11550.1 Fragaria-vesca
FvH4 7g11580.1 Fragaria-vesca
FvH4 6g01690.1 Fragaria-vesca
evm.TU.scaf 30.139 Fragaria-iinumae
GWHPAAYT037748 Pyrus-betulifolia
MD04G1022500 Malus-domestica
MABA016420 Malus-baccata

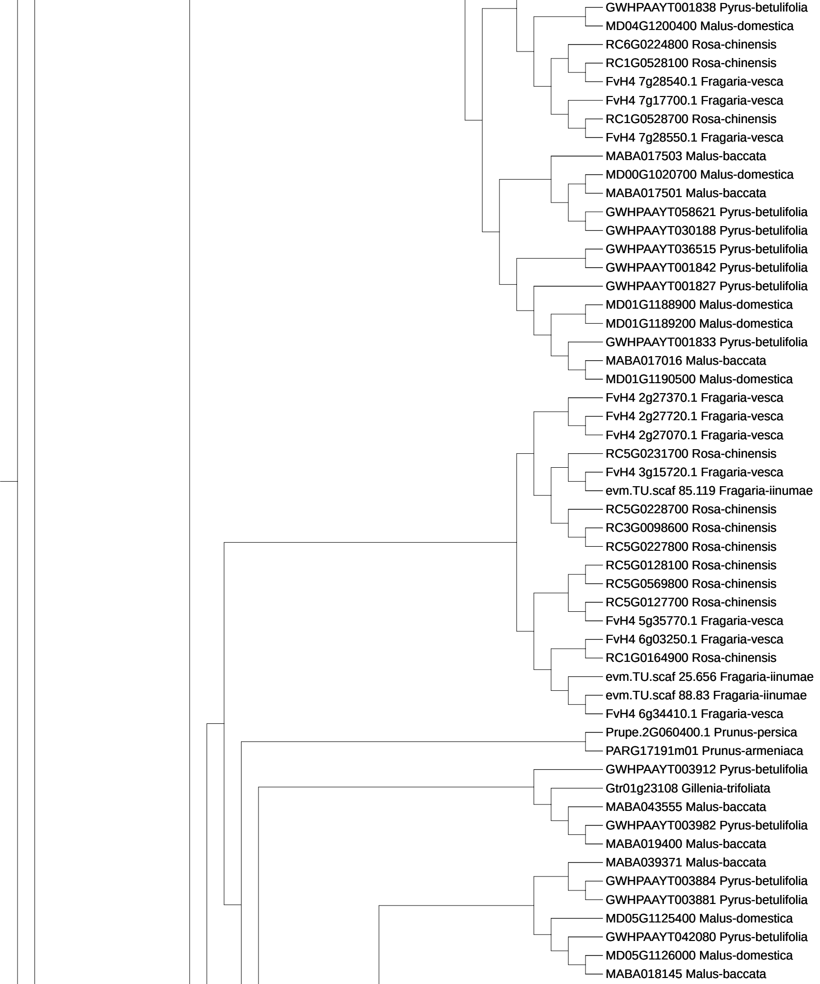
GWHPAAYT001838 Pyrus-betulifolia
MD04G1200400 Malus-domestica
RC6G0224800 Rosa-chinensis
RC1G0528100 Rosa-chinensis
FvH4 7g28540.1 Fragaria-vesca
FvH4 7g17700.1 Fragaria-vesca
RC1G0528700 Rosa-chinensis
FvH4 7g28550.1 Fragaria-vesca
MABA017503 Malus-baccata
MD00G1020700 Malus-domestica
MABA017501 Malus-baccata
GWHPAAYT058621 Pyrus-betulifolia
GWHPAAYT030188 Pyrus-betulifolia
GWHPAAYT036515 Pyrus-betulifolia
GWHPAAYT001842 Pyrus-betulifolia
GWHPAAYT001827 Pyrus-betulifolia
MD01G1188900 Malus-domestica
MD01G1189200 Malus-domestica
GWHPAAYT001833 Pyrus-betulifolia
MABA017016 Malus-baccata
MD01G1190500 Malus-domestica
FvH4 2g27370.1 Fragaria-vesca
FvH4 2g27720.1 Fragaria-vesca
FvH4 2g27070.1 Fragaria-vesca
RC5G0231700 Rosa-chinensis
FvH4 3g15720.1 Fragaria-vesca
evm.TU.scaf 85.119 Fragaria-iinumae
RC5G0228700 Rosa-chinensis
RC3G0098600 Rosa-chinensis
RC5G0227800 Rosa-chinensis
RC5G0128100 Rosa-chinensis
RC5G0569800 Rosa-chinensis
RC5G0127700 Rosa-chinensis
FvH4 5g35770.1 Fragaria-vesca
FvH4 6g03250.1 Fragaria-vesca
RC1G0164900 Rosa-chinensis
evm.TU.scaf 25.656 Fragaria-iinumae
evm.TU.scaf 88.83 Fragaria-iinumae
FvH4 6g34410.1 Fragaria-vesca
Prupe.2G060400.1 Prunus-persica
PARG17191m01 Prunus-armeniaca
GWHPAAYT003912 Pyrus-betulifolia
Gtr01g23108 Gillenia-trifoliata
MABA043555 Malus-baccata
GWHPAAYT003982 Pyrus-betulifolia
MABA019400 Malus-baccata
MABA039371 Malus-baccata
GWHPAAYT003884 Pyrus-betulifolia
GWHPAAYT003881 Pyrus-betulifolia
MD05G1125400 Malus-domestica
GWHPAAYT042080 Pyrus-betulifolia
MD05G1126000 Malus-domestica
MABA018145 Malus-baccata

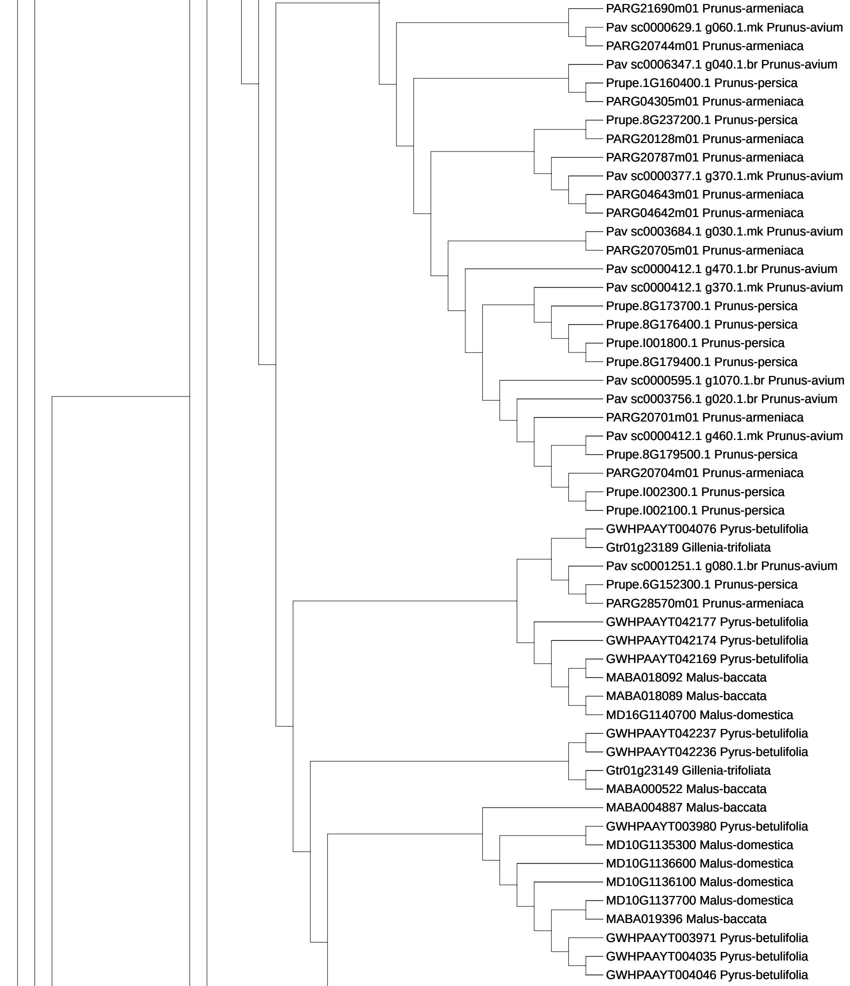
PARG21690m01 Prunus-armeniaca
Pav sc0000629.1 g060.1.mk Prunus-avium
PARG20744m01 Prunus-armeniaca
Pav sc0006347.1 g040.1.br Prunus-avium
Prupe.1G160400.1 Prunus-persica
PARG04305m01 Prunus-armeniaca
Prupe.8G237200.1 Prunus-persica
PARG20128m01 Prunus-armeniaca
PARG20787m01 Prunus-armeniaca
Pav sc0000377.1 g370.1.mk Prunus-avium
PARG04643m01 Prunus-armeniaca
PARG04642m01 Prunus-armeniaca
Pav sc0003684.1 g030.1.mk Prunus-avium
PARG20705m01 Prunus-armeniaca
Pav sc0000412.1 g470.1.br Prunus-avium
Pav sc0000412.1 g370.1.mk Prunus-avium
Prupe.8G173700.1 Prunus-persica
Prupe.8G176400.1 Prunus-persica
Prupe.I001800.1 Prunus-persica
Prupe.8G179400.1 Prunus-persica
Pav sc0000595.1 g1070.1.br Prunus-avium
Pav sc0003756.1 g020.1.br Prunus-avium
PARG20701m01 Prunus-armeniaca
Pav sc0000412.1 g460.1.mk Prunus-avium
Prupe.8G179500.1 Prunus-persica
PARG20704m01 Prunus-armeniaca
Prupe.I002300.1 Prunus-persica
Prupe.I002100.1 Prunus-persica
GWHPAAYT004076 Pyrus-betulifolia
Gtr01g23189 Gillenia-trifoliata
Pav sc0001251.1 g080.1.br Prunus-avium
Prupe.6G152300.1 Prunus-persica
PARG28570m01 Prunus-armeniaca
GWHPAAYT042177 Pyrus-betulifolia
GWHPAAYT042174 Pyrus-betulifolia
GWHPAAYT042169 Pyrus-betulifolia
MABA018092 Malus-baccata
MABA018089 Malus-baccata
MD16G1140700 Malus-domestica
GWHPAAYT042237 Pyrus-betulifolia
GWHPAAYT042236 Pyrus-betulifolia
Gtr01g23149 Gillenia-trifoliata
MABA000522 Malus-baccata
MABA004887 Malus-baccata
GWHPAAYT003980 Pyrus-betulifolia
MD10G1135300 Malus-domestica
MD10G1136600 Malus-domestica
MD10G1136100 Malus-domestica
MD10G1137700 Malus-domestica
MABA019396 Malus-baccata
GWHPAAYT003971 Pyrus-betulifolia
GWHPAAYT004035 Pyrus-betulifolia
GWHPAAYT004046 Pyrus-betulifolia

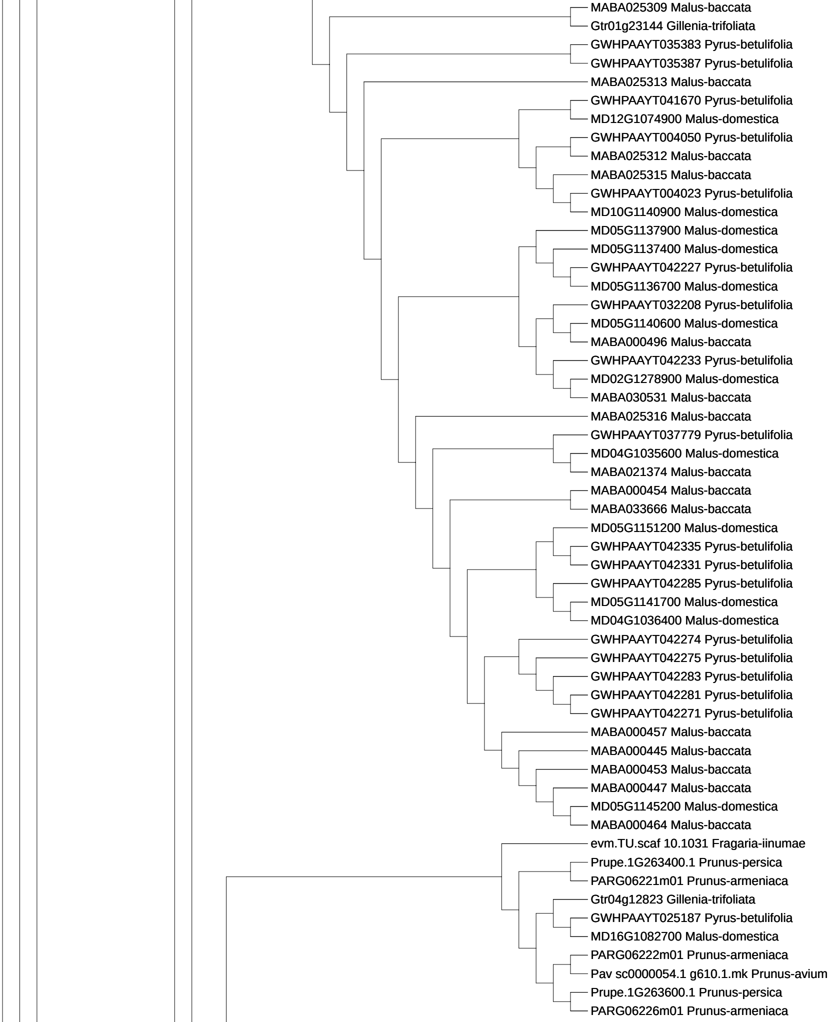
MABA025309 Malus-baccata
Gtr01g23144 Gillenia-trifoliata
GWHPAAYT035383 Pyrus-betulifolia
GWHPAAYT035387 Pyrus-betulifolia
MABA025313 Malus-baccata
GWHPAAYT041670 Pyrus-betulifolia
MD12G1074900 Malus-domestica
GWHPAAYT004050 Pyrus-betulifolia
MABA025312 Malus-baccata
MABA025315 Malus-baccata
GWHPAAYT004023 Pyrus-betulifolia
MD10G1140900 Malus-domestica
MD05G1137900 Malus-domestica
MD05G1137400 Malus-domestica
GWHPAAYT042227 Pyrus-betulifolia
MD05G1136700 Malus-domestica
GWHPAAYT032208 Pyrus-betulifolia
MD05G1140600 Malus-domestica
MABA000496 Malus-baccata
GWHPAAYT042233 Pyrus-betulifolia
MD02G1278900 Malus-domestica
MABA030531 Malus-baccata
MABA025316 Malus-baccata
GWHPAAYT037779 Pyrus-betulifolia
MD04G1035600 Malus-domestica
MABA021374 Malus-baccata
MABA000454 Malus-baccata
MABA033666 Malus-baccata
MD05G1151200 Malus-domestica
GWHPAAYT042335 Pyrus-betulifolia
GWHPAAYT042331 Pyrus-betulifolia
GWHPAAYT042285 Pyrus-betulifolia
MD05G1141700 Malus-domestica
MD04G1036400 Malus-domestica
GWHPAAYT042274 Pyrus-betulifolia
GWHPAAYT042275 Pyrus-betulifolia
GWHPAAYT042283 Pyrus-betulifolia
GWHPAAYT042281 Pyrus-betulifolia
GWHPAAYT042271 Pyrus-betulifolia
MABA000457 Malus-baccata
MABA000445 Malus-baccata
MABA000453 Malus-baccata
MABA000447 Malus-baccata
MD05G1145200 Malus-domestica
MABA000464 Malus-baccata
evm.TU.scaf 10.1031 Fragaria-iinumae
Prupe.1G263400.1 Prunus-persica
PARG06221m01 Prunus-armeniaca
Gtr04g12823 Gillenia-trifoliata
GWHPAAYT025187 Pyrus-betulifolia
MD16G1082700 Malus-domestica
PARG06222m01 Prunus-armeniaca
Pav sc0000054.1 g610.1.mk Prunus-avium
Prupe.1G263600.1 Prunus-persica
PARG06226m01 Prunus-armeniaca

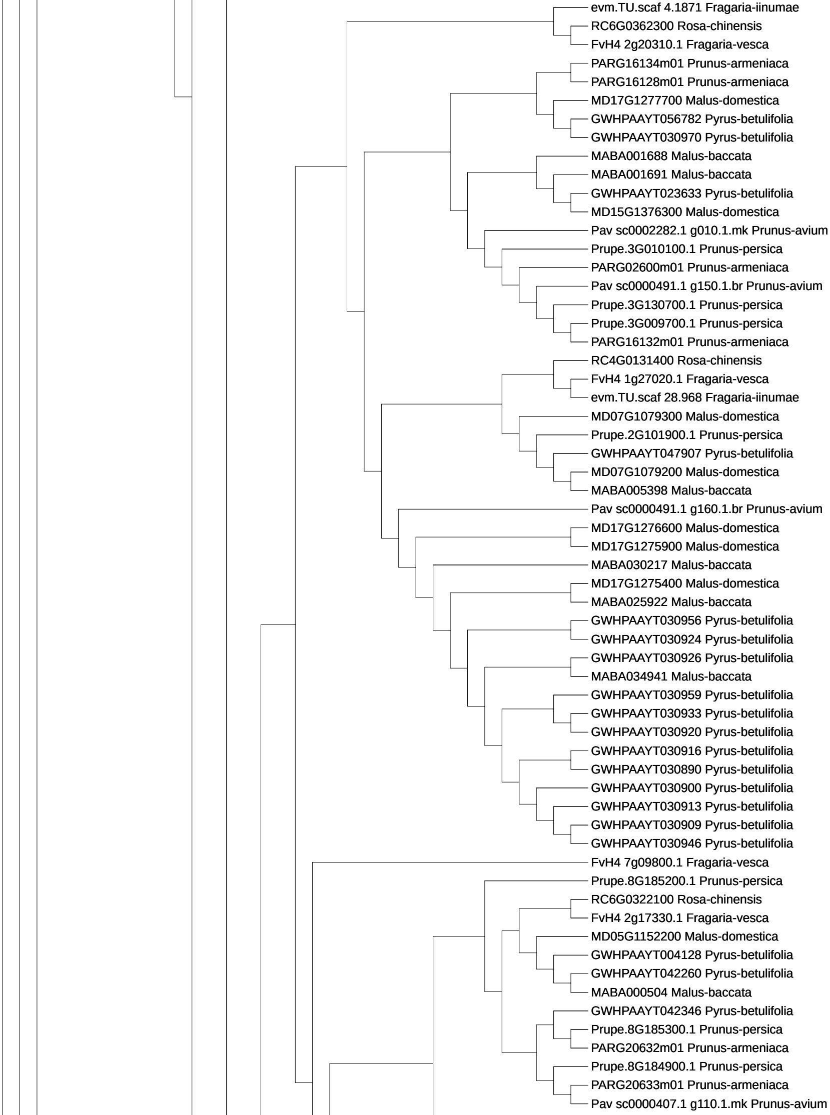
evm.TU.scaf 4.1871 Fragaria-iinumae
RC6G0362300 Rosa-chinensis
FvH4 2g20310.1 Fragaria-vesca
PARG16134m01 Prunus-armeniaca
PARG16128m01 Prunus-armeniaca
MD17G1277700 Malus-domestica
GWHPAAYT056782 Pyrus-betulifolia
GWHPAAYT030970 Pyrus-betulifolia
MABA001688 Malus-baccata
MABA001691 Malus-baccata
GWHPAAYT023633 Pyrus-betulifolia
MD15G1376300 Malus-domestica
Pav sc0002282.1 g010.1.mk Prunus-avium
Prupe.3G010100.1 Prunus-persica
PARG02600m01 Prunus-armeniaca
Pav sc0000491.1 g150.1.br Prunus-avium
Prupe.3G130700.1 Prunus-persica
Prupe.3G009700.1 Prunus-persica
PARG16132m01 Prunus-armeniaca
RC4G0131400 Rosa-chinensis
FvH4 1g27020.1 Fragaria-vesca
evm.TU.scaf 28.968 Fragaria-iinumae
MD07G1079300 Malus-domestica
Prupe.2G101900.1 Prunus-persica
GWHPAAYT047907 Pyrus-betulifolia
MD07G1079200 Malus-domestica
MABA005398 Malus-baccata
Pav sc0000491.1 g160.1.br Prunus-avium
MD17G1276600 Malus-domestica
MD17G1275900 Malus-domestica
MABA030217 Malus-baccata
MD17G1275400 Malus-domestica
MABA025922 Malus-baccata
GWHPAAYT030956 Pyrus-betulifolia
GWHPAAYT030924 Pyrus-betulifolia
GWHPAAYT030926 Pyrus-betulifolia
MABA034941 Malus-baccata
GWHPAAYT030959 Pyrus-betulifolia
GWHPAAYT030933 Pyrus-betulifolia
GWHPAAYT030920 Pyrus-betulifolia
GWHPAAYT030916 Pyrus-betulifolia
GWHPAAYT030890 Pyrus-betulifolia
GWHPAAYT030900 Pyrus-betulifolia
GWHPAAYT030913 Pyrus-betulifolia
GWHPAAYT030909 Pyrus-betulifolia
GWHPAAYT030946 Pyrus-betulifolia
FvH4 7g09800.1 Fragaria-vesca
Prupe.8G185200.1 Prunus-persica
RC6G0322100 Rosa-chinensis
FvH4 2g17330.1 Fragaria-vesca
MD05G1152200 Malus-domestica
GWHPAAYT004128 Pyrus-betulifolia
GWHPAAYT042260 Pyrus-betulifolia
MABA000504 Malus-baccata
GWHPAAYT042346 Pyrus-betulifolia
Prupe.8G185300.1 Prunus-persica
PARG20632m01 Prunus-armeniaca
Prupe.8G184900.1 Prunus-persica
PARG20633m01 Prunus-armeniaca
Pav sc0000407.1 g110.1.mk Prunus-avium

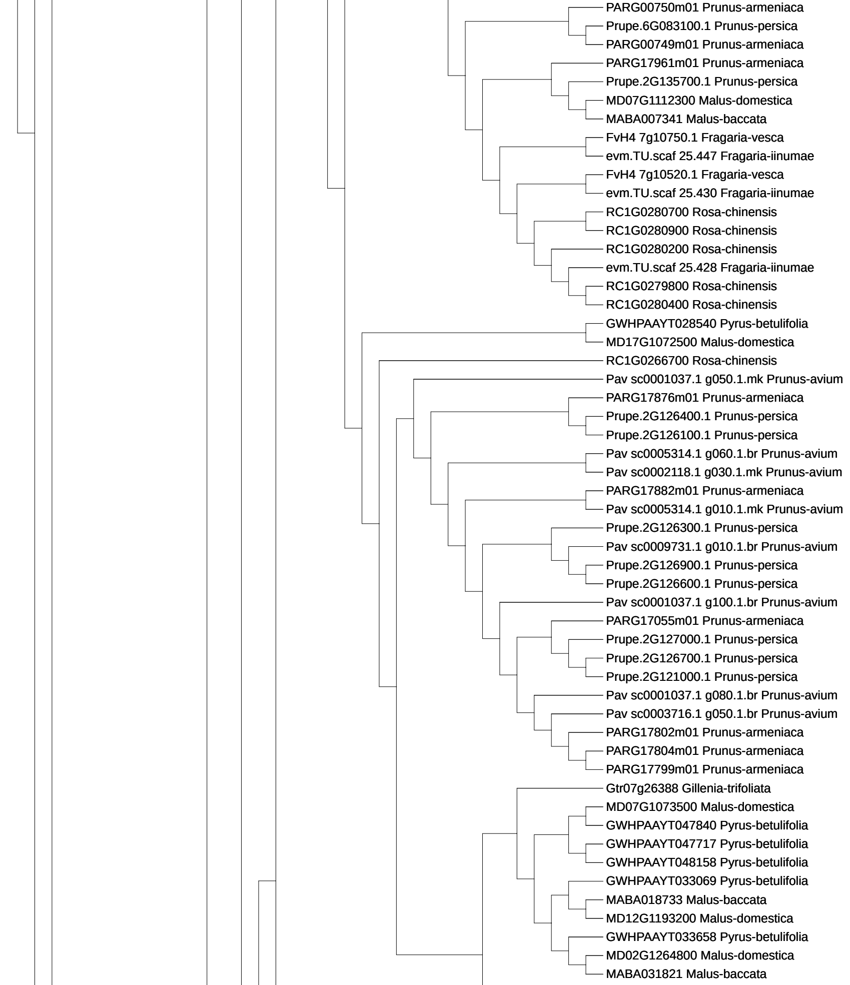
PARG00750m01 Prunus-armeniaca
Prupe.6G083100.1 Prunus-persica
PARG00749m01 Prunus-armeniaca
PARG17961m01 Prunus-armeniaca
Prupe.2G135700.1 Prunus-persica
MD07G1112300 Malus-domestica
MABA007341 Malus-baccata
FvH4 7g10750.1 Fragaria-vesca
evm.TU.scaf 25.447 Fragaria-iinumae
FvH4 7g10520.1 Fragaria-vesca
evm.TU.scaf 25.430 Fragaria-iinumae
RC1G0280700 Rosa-chinensis
RC1G0280900 Rosa-chinensis
RC1G0280200 Rosa-chinensis
evm.TU.scaf 25.428 Fragaria-iinumae
RC1G0279800 Rosa-chinensis
RC1G0280400 Rosa-chinensis
GWHPAAYT028540 Pyrus-betulifolia
MD17G1072500 Malus-domestica
RC1G0266700 Rosa-chinensis
Pav sc0001037.1 g050.1.mk Prunus-avium
PARG17876m01 Prunus-armeniaca
Prupe.2G126400.1 Prunus-persica
Prupe.2G126100.1 Prunus-persica
Pav sc0005314.1 g060.1.br Prunus-avium
Pav sc0002118.1 g030.1.mk Prunus-avium
PARG17882m01 Prunus-armeniaca
Pav sc0005314.1 g010.1.mk Prunus-avium
Prupe.2G126300.1 Prunus-persica
Pav sc0009731.1 g010.1.br Prunus-avium
Prupe.2G126900.1 Prunus-persica
Prupe.2G126600.1 Prunus-persica
Pav sc0001037.1 g100.1.br Prunus-avium
PARG17055m01 Prunus-armeniaca
Prupe.2G127000.1 Prunus-persica
Prupe.2G126700.1 Prunus-persica
Prupe.2G121000.1 Prunus-persica
Pav sc0001037.1 g080.1.br Prunus-avium
Pav sc0003716.1 g050.1.br Prunus-avium
PARG17802m01 Prunus-armeniaca
PARG17804m01 Prunus-armeniaca
PARG17799m01 Prunus-armeniaca
Gtr07g26388 Gillenia-trifoliata
MD07G1073500 Malus-domestica
GWHPAAYT047840 Pyrus-betulifolia
GWHPAAYT047717 Pyrus-betulifolia
GWHPAAYT048158 Pyrus-betulifolia
GWHPAAYT033069 Pyrus-betulifolia
MABA018733 Malus-baccata
MD12G1193200 Malus-domestica
GWHPAAYT033658 Pyrus-betulifolia
MD02G1264800 Malus-domestica
MABA031821 Malus-baccata

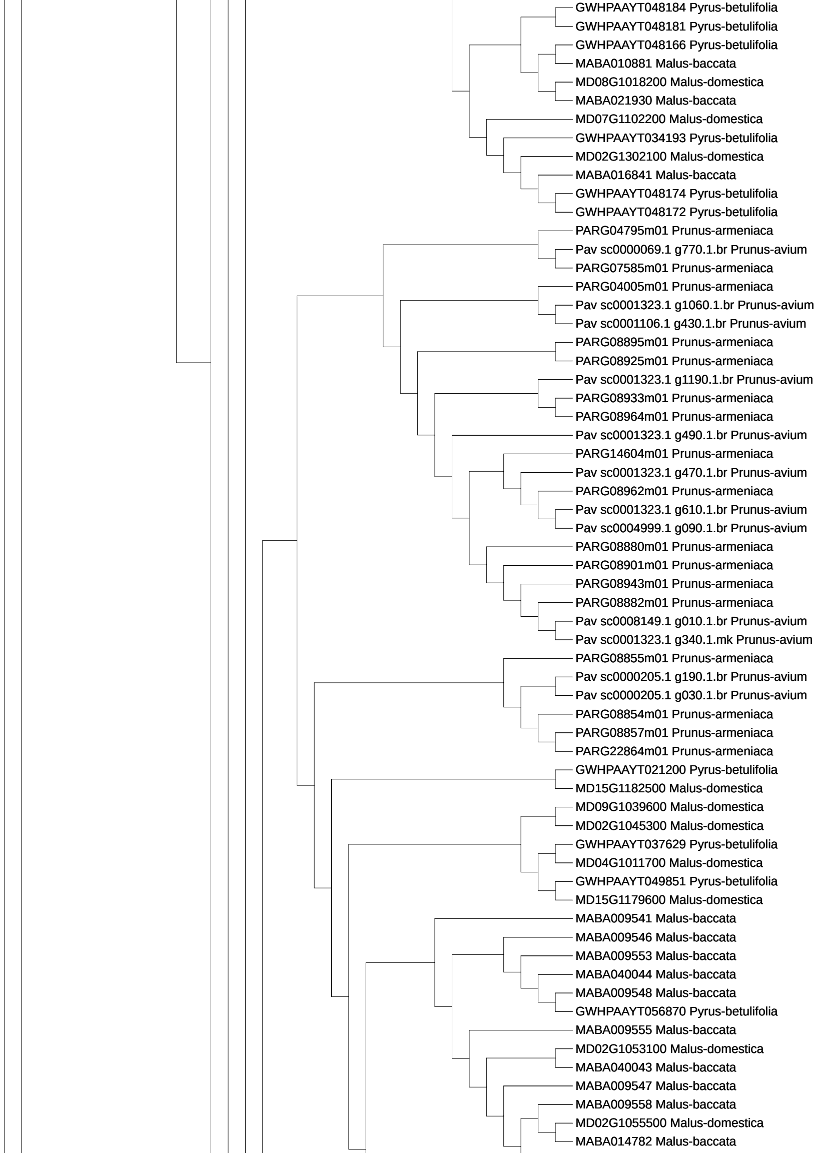
GWHPAAYT048184 Pyrus-betulifolia
GWHPAAYT048181 Pyrus-betulifolia
GWHPAAYT048166 Pyrus-betulifolia
MABA010881 Malus-baccata
MD08G1018200 Malus-domestica
MABA021930 Malus-baccata
MD07G1102200 Malus-domestica
GWHPAAYT034193 Pyrus-betulifolia
MD02G1302100 Malus-domestica
MABA016841 Malus-baccata
GWHPAAYT048174 Pyrus-betulifolia
GWHPAAYT048172 Pyrus-betulifolia
PARG04795m01 Prunus-armeniaca
Pav sc0000069.1 g770.1.br Prunus-avium
PARG07585m01 Prunus-armeniaca
PARG04005m01 Prunus-armeniaca
Pav sc0001323.1 g1060.1.br Prunus-avium
Pav sc0001106.1 g430.1.br Prunus-avium
PARG08895m01 Prunus-armeniaca
PARG08925m01 Prunus-armeniaca
Pav sc0001323.1 g1190.1.br Prunus-avium
PARG08933m01 Prunus-armeniaca
PARG08964m01 Prunus-armeniaca
Pav sc0001323.1 g490.1.br Prunus-avium
PARG14604m01 Prunus-armeniaca
Pav sc0001323.1 g470.1.br Prunus-avium
PARG08962m01 Prunus-armeniaca
Pav sc0001323.1 g610.1.br Prunus-avium
Pav sc0004999.1 g090.1.br Prunus-avium
PARG08880m01 Prunus-armeniaca
PARG08901m01 Prunus-armeniaca
PARG08943m01 Prunus-armeniaca
PARG08882m01 Prunus-armeniaca
Pav sc0008149.1 g010.1.br Prunus-avium
Pav sc0001323.1 g340.1.mk Prunus-avium
PARG08855m01 Prunus-armeniaca
Pav sc0000205.1 g190.1.br Prunus-avium
Pav sc0000205.1 g030.1.br Prunus-avium
PARG08854m01 Prunus-armeniaca
PARG08857m01 Prunus-armeniaca
PARG22864m01 Prunus-armeniaca
GWHPAAYT021200 Pyrus-betulifolia
MD15G1182500 Malus-domestica
MD09G1039600 Malus-domestica
MD02G1045300 Malus-domestica
GWHPAAYT037629 Pyrus-betulifolia
MD04G1011700 Malus-domestica
GWHPAAYT049851 Pyrus-betulifolia
MD15G1179600 Malus-domestica
MABA009541 Malus-baccata
MABA009546 Malus-baccata
MABA009553 Malus-baccata
MABA040044 Malus-baccata
MABA009548 Malus-baccata
GWHPAAYT056870 Pyrus-betulifolia
MABA009555 Malus-baccata
MD02G1053100 Malus-domestica
MABA040043 Malus-baccata
MABA009547 Malus-baccata
MABA009558 Malus-baccata
MD02G1055500 Malus-domestica
MABA014782 Malus-baccata

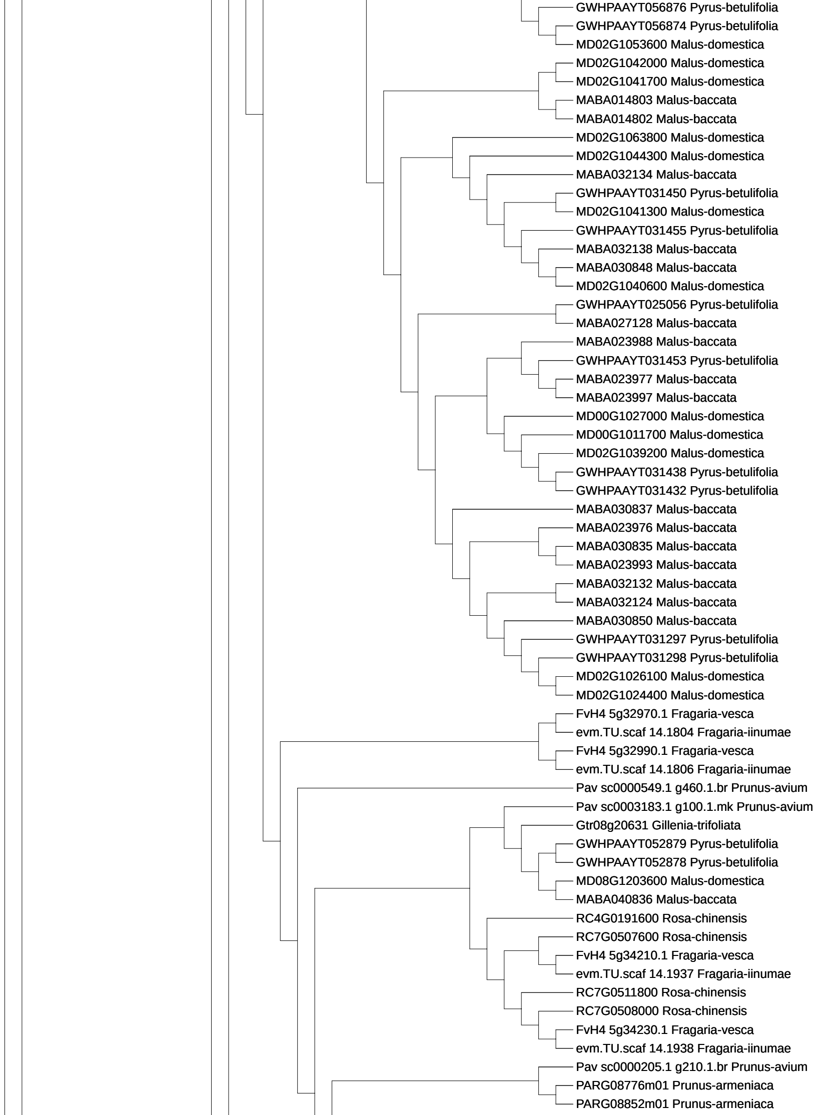
GWHPAAYT056876 Pyrus-betulifolia
GWHPAAYT056874 Pyrus-betulifolia
MD02G1053600 Malus-domestica
MD02G1042000 Malus-domestica
MD02G1041700 Malus-domestica
MABA014803 Malus-baccata
MABA014802 Malus-baccata
MD02G1063800 Malus-domestica
MD02G1044300 Malus-domestica
MABA032134 Malus-baccata
GWHPAAYT031450 Pyrus-betulifolia
MD02G1041300 Malus-domestica
GWHPAAYT031455 Pyrus-betulifolia
MABA032138 Malus-baccata
MABA030848 Malus-baccata
MD02G1040600 Malus-domestica
GWHPAAYT025056 Pyrus-betulifolia
MABA027128 Malus-baccata
MABA023988 Malus-baccata
GWHPAAYT031453 Pyrus-betulifolia
MABA023977 Malus-baccata
MABA023997 Malus-baccata
MD00G1027000 Malus-domestica
MD00G1011700 Malus-domestica
MD02G1039200 Malus-domestica
GWHPAAYT031438 Pyrus-betulifolia
GWHPAAYT031432 Pyrus-betulifolia
MABA030837 Malus-baccata
MABA023976 Malus-baccata
MABA030835 Malus-baccata
MABA023993 Malus-baccata
MABA032132 Malus-baccata
MABA032124 Malus-baccata
MABA030850 Malus-baccata
GWHPAAYT031297 Pyrus-betulifolia
GWHPAAYT031298 Pyrus-betulifolia
MD02G1026100 Malus-domestica
MD02G1024400 Malus-domestica
FvH4 5g32970.1 Fragaria-vesca
evm.TU.scaf 14.1804 Fragaria-iinumae
FvH4 5g32990.1 Fragaria-vesca
evm.TU.scaf 14.1806 Fragaria-iinumae
Pav sc0000549.1 g460.1.br Prunus-avium
Pav sc0003183.1 g100.1.mk Prunus-avium
Gtr08g20631 Gillenia-trifoliata
GWHPAAYT052879 Pyrus-betulifolia
GWHPAAYT052878 Pyrus-betulifolia
MD08G1203600 Malus-domestica
MABA040836 Malus-baccata
RC4G0191600 Rosa-chinensis
RC7G0507600 Rosa-chinensis
FvH4 5g34210.1 Fragaria-vesca
evm.TU.scaf 14.1937 Fragaria-iinumae
RC7G0511800 Rosa-chinensis
RC7G0508000 Rosa-chinensis
FvH4 5g34230.1 Fragaria-vesca
evm.TU.scaf 14.1938 Fragaria-iinumae
Pav sc0000205.1 g210.1.br Prunus-avium
PARG08776m01 Prunus-armeniaca
PARG08852m01 Prunus-armeniaca

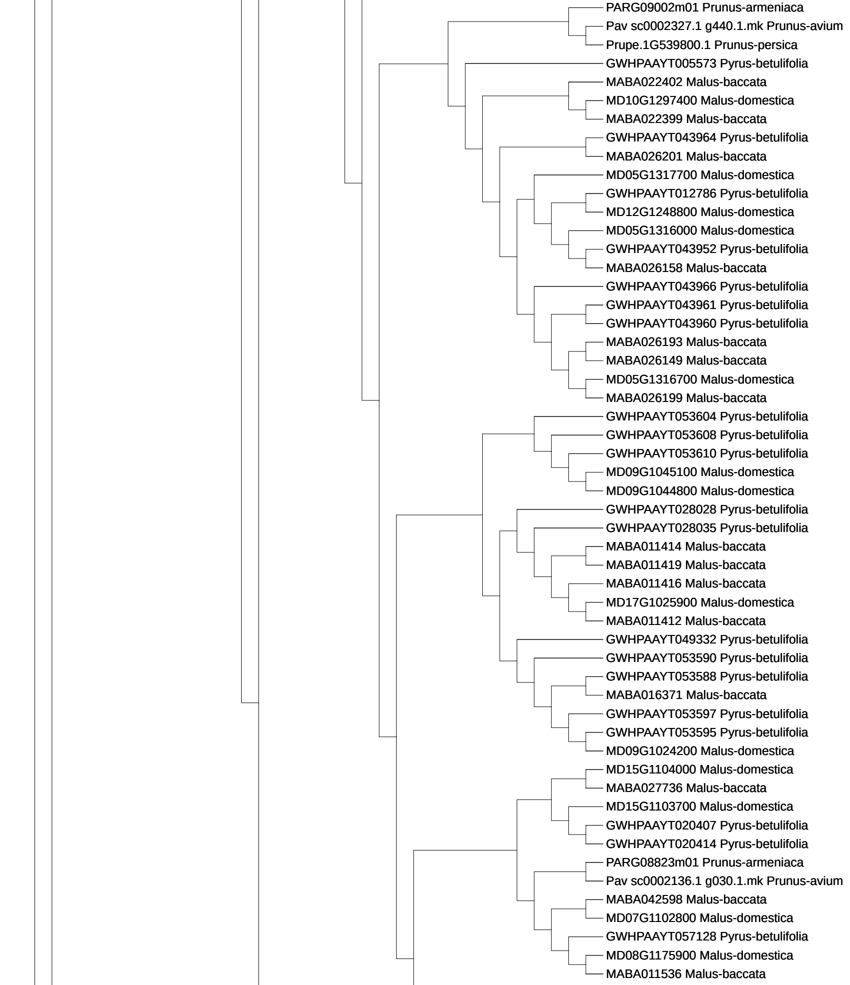
PARG09002m01 Prunus-armeniaca
Pav sc0002327.1 g440.1.mk Prunus-avium
Prupe.1G539800.1 Prunus-persica
GWHPAAYT005573 Pyrus-betulifolia
MABA022402 Malus-baccata
MD10G1297400 Malus-domestica
MABA022399 Malus-baccata
GWHPAAYT043964 Pyrus-betulifolia
MABA026201 Malus-baccata
MD05G1317700 Malus-domestica
GWHPAAYT012786 Pyrus-betulifolia
MD12G1248800 Malus-domestica
MD05G1316000 Malus-domestica
GWHPAAYT043952 Pyrus-betulifolia
MABA026158 Malus-baccata
GWHPAAYT043966 Pyrus-betulifolia
GWHPAAYT043961 Pyrus-betulifolia
GWHPAAYT043960 Pyrus-betulifolia
MABA026193 Malus-baccata
MABA026149 Malus-baccata
MD05G1316700 Malus-domestica
MABA026199 Malus-baccata
GWHPAAYT053604 Pyrus-betulifolia
GWHPAAYT053608 Pyrus-betulifolia
GWHPAAYT053610 Pyrus-betulifolia
MD09G1045100 Malus-domestica
MD09G1044800 Malus-domestica
GWHPAAYT028028 Pyrus-betulifolia
GWHPAAYT028035 Pyrus-betulifolia
MABA011414 Malus-baccata
MABA011419 Malus-baccata
MABA011416 Malus-baccata
MD17G1025900 Malus-domestica
MABA011412 Malus-baccata
GWHPAAYT049332 Pyrus-betulifolia
GWHPAAYT053590 Pyrus-betulifolia
GWHPAAYT053588 Pyrus-betulifolia
MABA016371 Malus-baccata
GWHPAAYT053597 Pyrus-betulifolia
GWHPAAYT053595 Pyrus-betulifolia
MD09G1024200 Malus-domestica
MD15G1104000 Malus-domestica
MABA027736 Malus-baccata
MD15G1103700 Malus-domestica
GWHPAAYT020407 Pyrus-betulifolia
GWHPAAYT020414 Pyrus-betulifolia
PARG08823m01 Prunus-armeniaca
Pav sc0002136.1 g030.1.mk Prunus-avium
MABA042598 Malus-baccata
MD07G1102800 Malus-domestica
GWHPAAYT057128 Pyrus-betulifolia
MD08G1175900 Malus-domestica
MABA011536 Malus-baccata

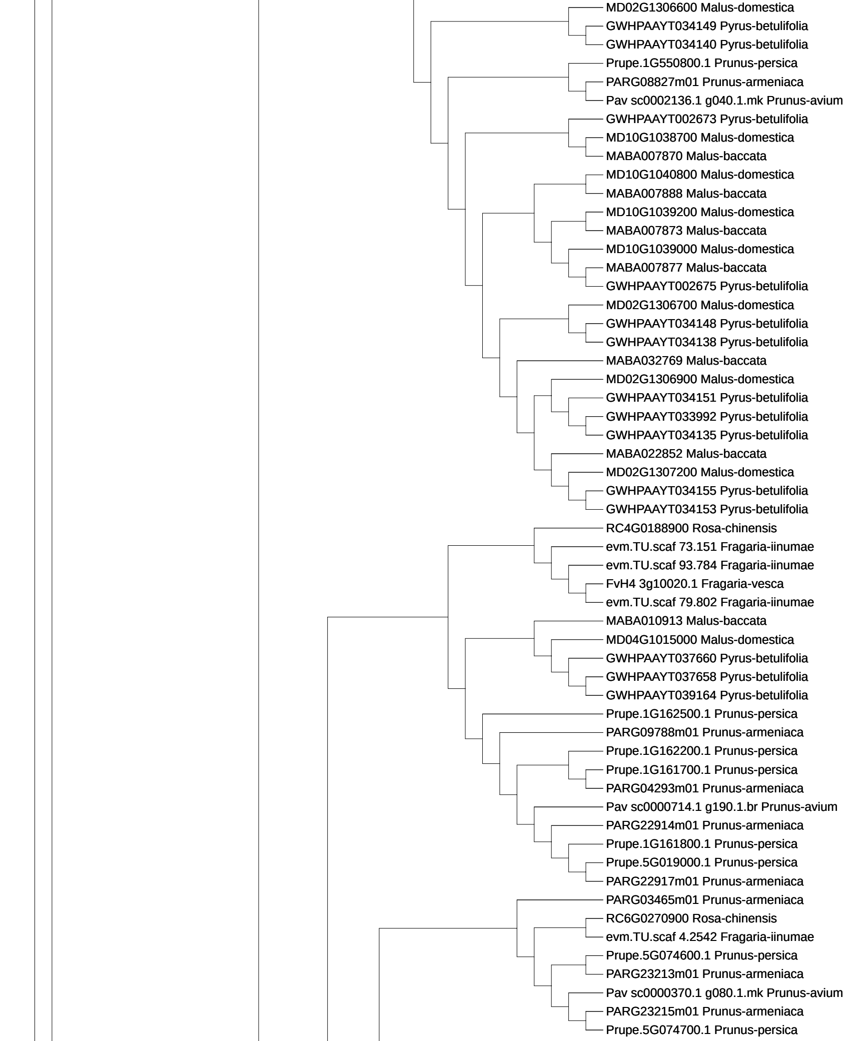
MD02G1306600 Malus-domestica
GWHPAAYT034149 Pyrus-betulifolia
GWHPAAYT034140 Pyrus-betulifolia
Prupe.1G550800.1 Prunus-persica
PARG08827m01 Prunus-armeniaca
Pav sc0002136.1 g040.1.mk Prunus-avium
GWHPAAYT002673 Pyrus-betulifolia
MD10G1038700 Malus-domestica
MABA007870 Malus-baccata
MD10G1040800 Malus-domestica
MABA007888 Malus-baccata
MD10G1039200 Malus-domestica
MABA007873 Malus-baccata
MD10G1039000 Malus-domestica
MABA007877 Malus-baccata
GWHPAAYT002675 Pyrus-betulifolia
MD02G1306700 Malus-domestica
GWHPAAYT034148 Pyrus-betulifolia
GWHPAAYT034138 Pyrus-betulifolia
MABA032769 Malus-baccata
MD02G1306900 Malus-domestica
GWHPAAYT034151 Pyrus-betulifolia
GWHPAAYT033992 Pyrus-betulifolia
GWHPAAYT034135 Pyrus-betulifolia
MABA022852 Malus-baccata
MD02G1307200 Malus-domestica
GWHPAAYT034155 Pyrus-betulifolia
GWHPAAYT034153 Pyrus-betulifolia
RC4G0188900 Rosa-chinensis
evm.TU.scaf 73.151 Fragaria-iinumae
evm.TU.scaf 93.784 Fragaria-iinumae
FvH4 3g10020.1 Fragaria-vesca
evm.TU.scaf 79.802 Fragaria-iinumae
MABA010913 Malus-baccata
MD04G1015000 Malus-domestica
GWHPAAYT037660 Pyrus-betulifolia
GWHPAAYT037658 Pyrus-betulifolia
GWHPAAYT039164 Pyrus-betulifolia
Prupe.1G162500.1 Prunus-persica
PARG09788m01 Prunus-armeniaca
Prupe.1G162200.1 Prunus-persica
Prupe.1G161700.1 Prunus-persica
PARG04293m01 Prunus-armeniaca
Pav sc0000714.1 g190.1.br Prunus-avium
PARG22914m01 Prunus-armeniaca
Prupe.1G161800.1 Prunus-persica
Prupe.5G019000.1 Prunus-persica
PARG22917m01 Prunus-armeniaca
PARG03465m01 Prunus-armeniaca
RC6G0270900 Rosa-chinensis
evm.TU.scaf 4.2542 Fragaria-iinumae
Prupe.5G074600.1 Prunus-persica
PARG23213m01 Prunus-armeniaca
Pav sc0000370.1 g080.1.mk Prunus-avium
PARG23215m01 Prunus-armeniaca
Prupe.5G074700.1 Prunus-persica

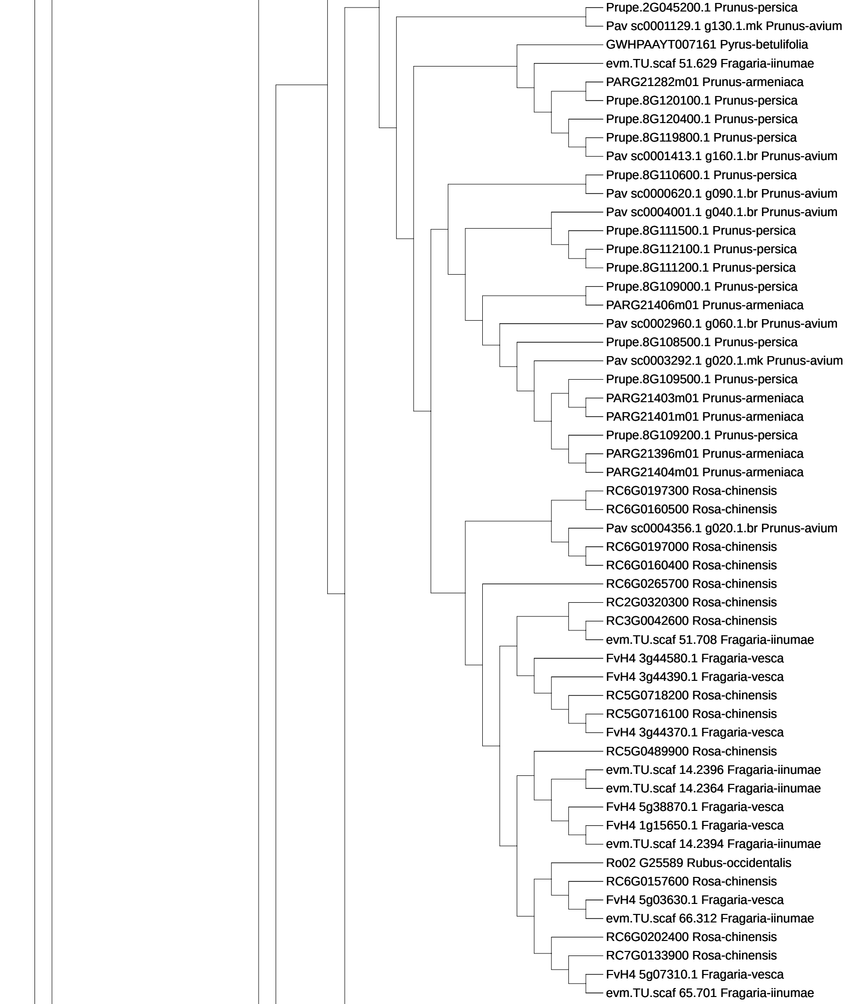
Prupe.2G045200.1 Prunus-persica
Pav sc0001129.1 g130.1.mk Prunus-avium
GWHPAAYT007161 Pyrus-betulifolia
evm.TU.scaf 51.629 Fragaria-iinumae
PARG21282m01 Prunus-armeniaca
Prupe.8G120100.1 Prunus-persica
Prupe.8G120400.1 Prunus-persica
Prupe.8G119800.1 Prunus-persica
Pav sc0001413.1 g160.1.br Prunus-avium
Prupe.8G110600.1 Prunus-persica
Pav sc0000620.1 g090.1.br Prunus-avium
Pav sc0004001.1 g040.1.br Prunus-avium
Prupe.8G111500.1 Prunus-persica
Prupe.8G112100.1 Prunus-persica
Prupe.8G111200.1 Prunus-persica
Prupe.8G109000.1 Prunus-persica
PARG21406m01 Prunus-armeniaca
Pav sc0002960.1 g060.1.br Prunus-avium
Prupe.8G108500.1 Prunus-persica
Pav sc0003292.1 g020.1.mk Prunus-avium
Prupe.8G109500.1 Prunus-persica
PARG21403m01 Prunus-armeniaca
PARG21401m01 Prunus-armeniaca
Prupe.8G109200.1 Prunus-persica
PARG21396m01 Prunus-armeniaca
PARG21404m01 Prunus-armeniaca
RC6G0197300 Rosa-chinensis
RC6G0160500 Rosa-chinensis
Pav sc0004356.1 g020.1.br Prunus-avium
RC6G0197000 Rosa-chinensis
RC6G0160400 Rosa-chinensis
RC6G0265700 Rosa-chinensis
RC2G0320300 Rosa-chinensis
RC3G0042600 Rosa-chinensis
evm.TU.scaf 51.708 Fragaria-iinumae
FvH4 3g44580.1 Fragaria-vesca
FvH4 3g44390.1 Fragaria-vesca
RC5G0718200 Rosa-chinensis
RC5G0716100 Rosa-chinensis
FvH4 3g44370.1 Fragaria-vesca
RC5G0489900 Rosa-chinensis
evm.TU.scaf 14.2396 Fragaria-iinumae
evm.TU.scaf 14.2364 Fragaria-iinumae
FvH4 5g38870.1 Fragaria-vesca
FvH4 1g15650.1 Fragaria-vesca
evm.TU.scaf 14.2394 Fragaria-iinumae
Ro02 G25589 Rubus-occidentalis
RC6G0157600 Rosa-chinensis
FvH4 5g03630.1 Fragaria-vesca
evm.TU.scaf 66.312 Fragaria-iinumae
RC6G0202400 Rosa-chinensis
RC7G0133900 Rosa-chinensis
FvH4 5g07310.1 Fragaria-vesca
evm.TU.scaf 65.701 Fragaria-iinumae

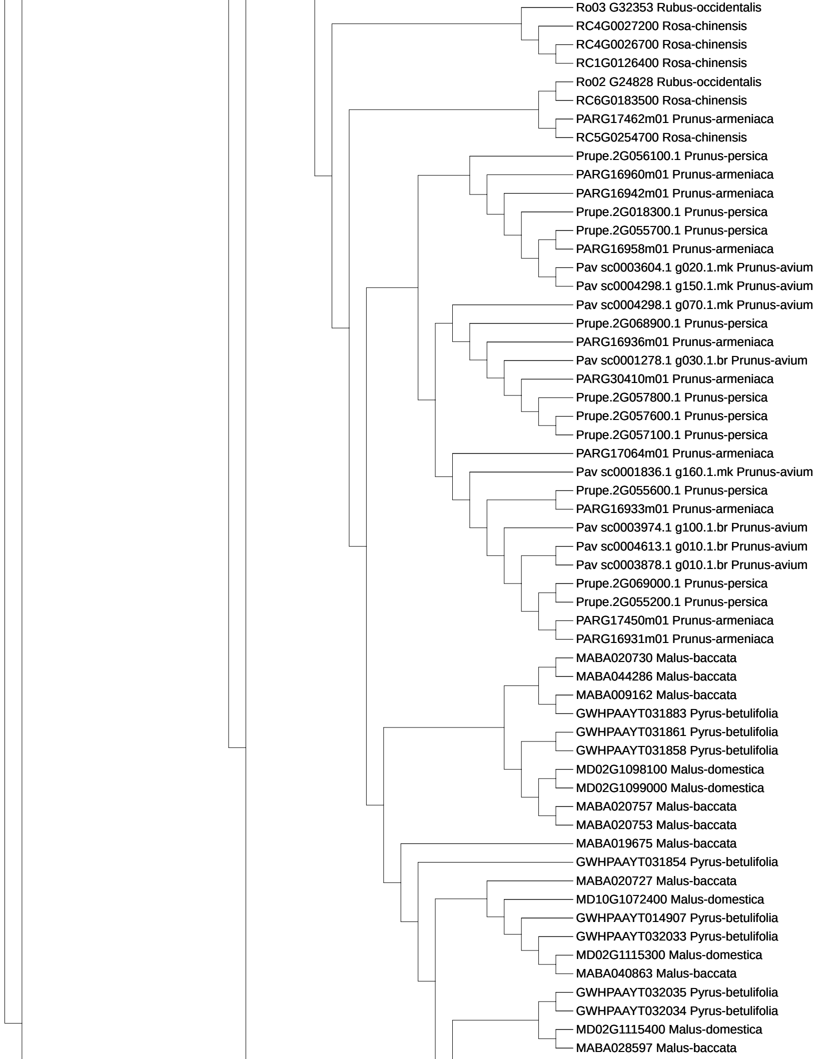
Ro03 G32353 Rubus-occidentalis
RC4G0027200 Rosa-chinensis
RC4G0026700 Rosa-chinensis
RC1G0126400 Rosa-chinensis
Ro02 G24828 Rubus-occidentalis
RC6G0183500 Rosa-chinensis
PARG17462m01 Prunus-armeniaca
RC5G0254700 Rosa-chinensis
Prupe.2G056100.1 Prunus-persica
PARG16960m01 Prunus-armeniaca
PARG16942m01 Prunus-armeniaca
Prupe.2G018300.1 Prunus-persica
Prupe.2G055700.1 Prunus-persica
PARG16958m01 Prunus-armeniaca
Pav sc0003604.1 g020.1.mk Prunus-avium
Pav sc0004298.1 g150.1.mk Prunus-avium
Pav sc0004298.1 g070.1.mk Prunus-avium
Prupe.2G068900.1 Prunus-persica
PARG16936m01 Prunus-armeniaca
Pav sc0001278.1 g030.1.br Prunus-avium
PARG30410m01 Prunus-armeniaca
Prupe.2G057800.1 Prunus-persica
Prupe.2G057600.1 Prunus-persica
Prupe.2G057100.1 Prunus-persica
PARG17064m01 Prunus-armeniaca
Pav sc0001836.1 g160.1.mk Prunus-avium
Prupe.2G055600.1 Prunus-persica
PARG16933m01 Prunus-armeniaca
Pav sc0003974.1 g100.1.br Prunus-avium
Pav sc0004613.1 g010.1.br Prunus-avium
Pav sc0003878.1 g010.1.br Prunus-avium
Prupe.2G069000.1 Prunus-persica
Prupe.2G055200.1 Prunus-persica
PARG17450m01 Prunus-armeniaca
PARG16931m01 Prunus-armeniaca
MABA020730 Malus-baccata
MABA044286 Malus-baccata
MABA009162 Malus-baccata
GWHPAAYT031883 Pyrus-betulifolia
GWHPAAYT031861 Pyrus-betulifolia
GWHPAAYT031858 Pyrus-betulifolia
MD02G1098100 Malus-domestica
MD02G1099000 Malus-domestica
MABA020757 Malus-baccata
MABA020753 Malus-baccata
MABA019675 Malus-baccata
GWHPAAYT031854 Pyrus-betulifolia
MABA020727 Malus-baccata
MD10G1072400 Malus-domestica
GWHPAAYT014907 Pyrus-betulifolia
GWHPAAYT032033 Pyrus-betulifolia
MD02G1115300 Malus-domestica
MABA040863 Malus-baccata
GWHPAAYT032035 Pyrus-betulifolia
GWHPAAYT032034 Pyrus-betulifolia
MD02G1115400 Malus-domestica
MABA028597 Malus-baccata

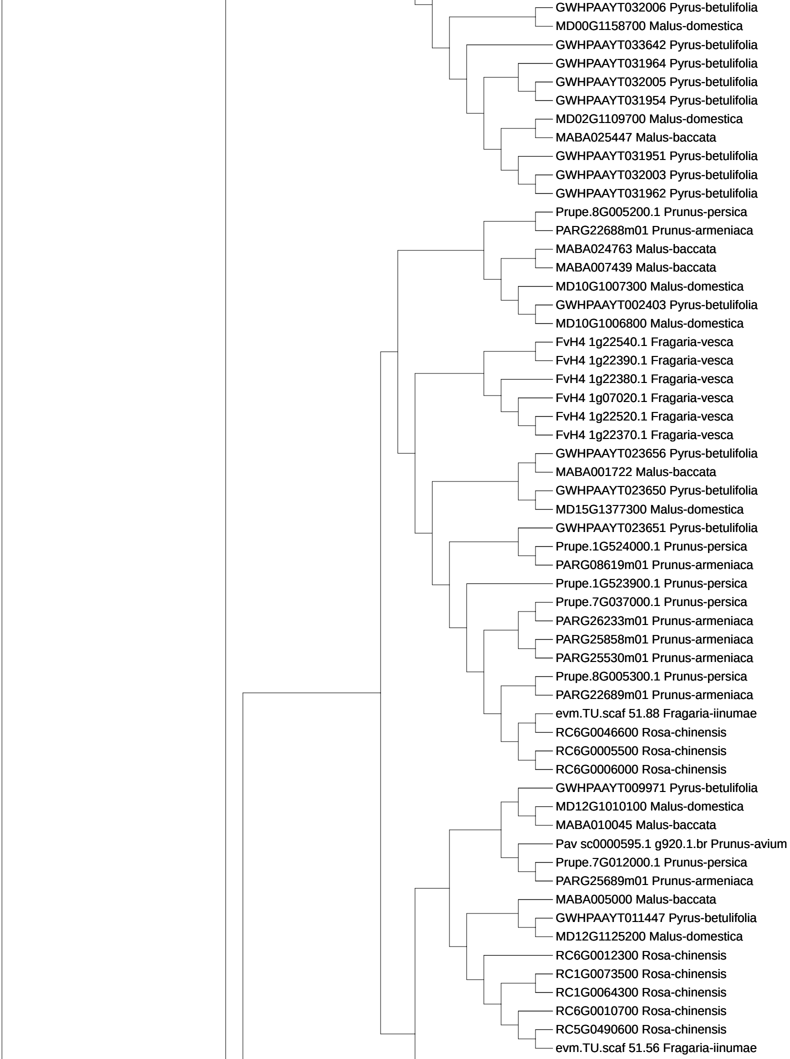
GWHPAAYT032006 Pyrus-betulifolia
MD00G1158700 Malus-domestica
GWHPAAYT033642 Pyrus-betulifolia
GWHPAAYT031964 Pyrus-betulifolia
GWHPAAYT032005 Pyrus-betulifolia
GWHPAAYT031954 Pyrus-betulifolia
MD02G1109700 Malus-domestica
MABA025447 Malus-baccata
GWHPAAYT031951 Pyrus-betulifolia
GWHPAAYT032003 Pyrus-betulifolia
GWHPAAYT031962 Pyrus-betulifolia
Prupe.8G005200.1 Prunus-persica
PARG22688m01 Prunus-armeniaca
MABA024763 Malus-baccata
MABA007439 Malus-baccata
MD10G1007300 Malus-domestica
GWHPAAYT002403 Pyrus-betulifolia
MD10G1006800 Malus-domestica
FvH4 1g22540.1 Fragaria-vesca
FvH4 1g22390.1 Fragaria-vesca
FvH4 1g22380.1 Fragaria-vesca
FvH4 1g07020.1 Fragaria-vesca
FvH4 1g22520.1 Fragaria-vesca
FvH4 1g22370.1 Fragaria-vesca
GWHPAAYT023656 Pyrus-betulifolia
MABA001722 Malus-baccata
GWHPAAYT023650 Pyrus-betulifolia
MD15G1377300 Malus-domestica
GWHPAAYT023651 Pyrus-betulifolia
Prupe.1G524000.1 Prunus-persica
PARG08619m01 Prunus-armeniaca
Prupe.1G523900.1 Prunus-persica
Prupe.7G037000.1 Prunus-persica
PARG26233m01 Prunus-armeniaca
PARG25858m01 Prunus-armeniaca
PARG25530m01 Prunus-armeniaca
Prupe.8G005300.1 Prunus-persica
PARG22689m01 Prunus-armeniaca
evm.TU.scaf 51.88 Fragaria-iinumae
RC6G0046600 Rosa-chinensis
RC6G0005500 Rosa-chinensis
RC6G0006000 Rosa-chinensis
GWHPAAYT009971 Pyrus-betulifolia
MD12G1010100 Malus-domestica
MABA010045 Malus-baccata
Pav sc0000595.1 g920.1.br Prunus-avium
Prupe.7G012000.1 Prunus-persica
PARG25689m01 Prunus-armeniaca
MABA005000 Malus-baccata
GWHPAAYT011447 Pyrus-betulifolia
MD12G1125200 Malus-domestica
RC6G0012300 Rosa-chinensis
RC1G0073500 Rosa-chinensis
RC1G0064300 Rosa-chinensis
RC6G0010700 Rosa-chinensis
RC5G0490600 Rosa-chinensis
evm.TU.scaf 51.56 Fragaria-iinumae

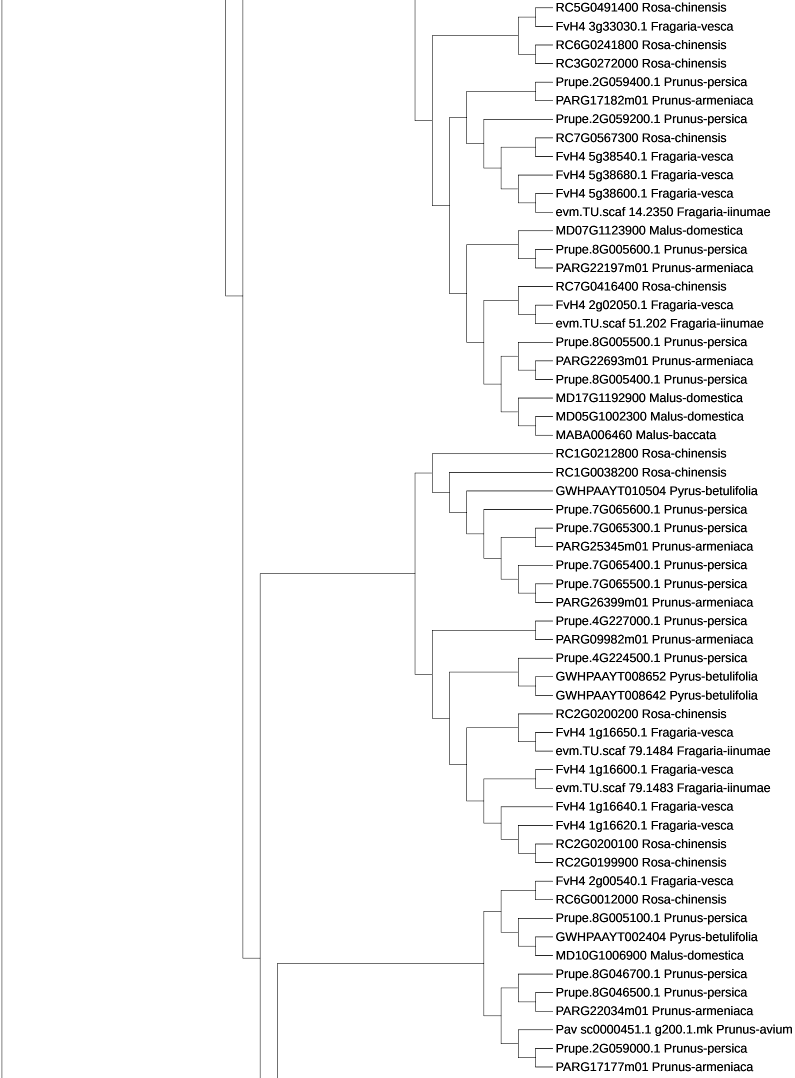
RC5G0491400 Rosa-chinensis
FvH4 3g33030.1 Fragaria-vesca
RC6G0241800 Rosa-chinensis
RC3G0272000 Rosa-chinensis
Prupe.2G059400.1 Prunus-persica
PARG17182m01 Prunus-armeniaca
Prupe.2G059200.1 Prunus-persica
RC7G0567300 Rosa-chinensis
FvH4 5g38540.1 Fragaria-vesca
FvH4 5g38680.1 Fragaria-vesca
FvH4 5g38600.1 Fragaria-vesca
evm.TU.scaf 14.2350 Fragaria-iinumae
MD07G1123900 Malus-domestica
Prupe.8G005600.1 Prunus-persica
PARG22197m01 Prunus-armeniaca
RC7G0416400 Rosa-chinensis
FvH4 2g02050.1 Fragaria-vesca
evm.TU.scaf 51.202 Fragaria-iinumae
Prupe.8G005500.1 Prunus-persica
PARG22693m01 Prunus-armeniaca
Prupe.8G005400.1 Prunus-persica
MD17G1192900 Malus-domestica
MD05G1002300 Malus-domestica
MABA006460 Malus-baccata
RC1G0212800 Rosa-chinensis
RC1G0038200 Rosa-chinensis
GWHPAAYT010504 Pyrus-betulifolia
Prupe.7G065600.1 Prunus-persica
Prupe.7G065300.1 Prunus-persica
PARG25345m01 Prunus-armeniaca
Prupe.7G065400.1 Prunus-persica
Prupe.7G065500.1 Prunus-persica
PARG26399m01 Prunus-armeniaca
Prupe.4G227000.1 Prunus-persica
PARG09982m01 Prunus-armeniaca
Prupe.4G224500.1 Prunus-persica
GWHPAAYT008652 Pyrus-betulifolia
GWHPAAYT008642 Pyrus-betulifolia
RC2G0200200 Rosa-chinensis
FvH4 1g16650.1 Fragaria-vesca
evm.TU.scaf 79.1484 Fragaria-iinumae
FvH4 1g16600.1 Fragaria-vesca
evm.TU.scaf 79.1483 Fragaria-iinumae
FvH4 1g16640.1 Fragaria-vesca
FvH4 1g16620.1 Fragaria-vesca
RC2G0200100 Rosa-chinensis
RC2G0199900 Rosa-chinensis
FvH4 2g00540.1 Fragaria-vesca
RC6G0012000 Rosa-chinensis
Prupe.8G005100.1 Prunus-persica
GWHPAAYT002404 Pyrus-betulifolia
MD10G1006900 Malus-domestica
Prupe.8G046700.1 Prunus-persica
Prupe.8G046500.1 Prunus-persica
PARG22034m01 Prunus-armeniaca
Pav sc0000451.1 g200.1.mk Prunus-avium
Prupe.2G059000.1 Prunus-persica
PARG17177m01 Prunus-armeniaca

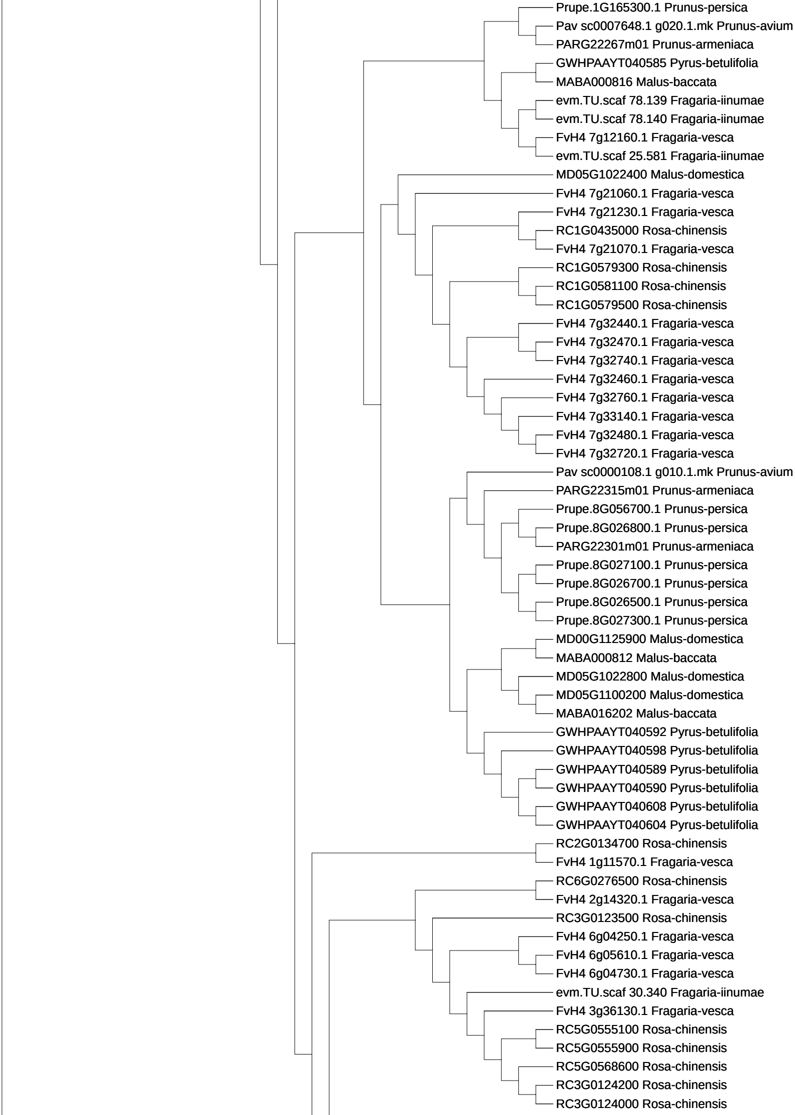
Prupe.1G165300.1 Prunus-persica
Pav sc0007648.1 g020.1.mk Prunus-avium
PARG22267m01 Prunus-armeniaca
GWHPAAYT040585 Pyrus-betulifolia
MABA000816 Malus-baccata
evm.TU.scaf 78.139 Fragaria-iinumae
evm.TU.scaf 78.140 Fragaria-iinumae
FvH4 7g12160.1 Fragaria-vesca
evm.TU.scaf 25.581 Fragaria-iinumae
MD05G1022400 Malus-domestica
FvH4 7g21060.1 Fragaria-vesca
FvH4 7g21230.1 Fragaria-vesca
RC1G0435000 Rosa-chinensis
FvH4 7g21070.1 Fragaria-vesca
RC1G0579300 Rosa-chinensis
RC1G0581100 Rosa-chinensis
RC1G0579500 Rosa-chinensis
FvH4 7g32440.1 Fragaria-vesca
FvH4 7g32470.1 Fragaria-vesca
FvH4 7g32740.1 Fragaria-vesca
FvH4 7g32460.1 Fragaria-vesca
FvH4 7g32760.1 Fragaria-vesca
FvH4 7g33140.1 Fragaria-vesca
FvH4 7g32480.1 Fragaria-vesca
FvH4 7g32720.1 Fragaria-vesca
Pav sc0000108.1 g010.1.mk Prunus-avium
PARG22315m01 Prunus-armeniaca
Prupe.8G056700.1 Prunus-persica
Prupe.8G026800.1 Prunus-persica
PARG22301m01 Prunus-armeniaca
Prupe.8G027100.1 Prunus-persica
Prupe.8G026700.1 Prunus-persica
Prupe.8G026500.1 Prunus-persica
Prupe.8G027300.1 Prunus-persica
MD00G1125900 Malus-domestica
MABA000812 Malus-baccata
MD05G1022800 Malus-domestica
MD05G1100200 Malus-domestica
MABA016202 Malus-baccata
GWHPAAYT040592 Pyrus-betulifolia
GWHPAAYT040598 Pyrus-betulifolia
GWHPAAYT040589 Pyrus-betulifolia
GWHPAAYT040590 Pyrus-betulifolia
GWHPAAYT040608 Pyrus-betulifolia
GWHPAAYT040604 Pyrus-betulifolia
RC2G0134700 Rosa-chinensis
FvH4 1g11570.1 Fragaria-vesca
RC6G0276500 Rosa-chinensis
FvH4 2g14320.1 Fragaria-vesca
RC3G0123500 Rosa-chinensis
FvH4 6g04250.1 Fragaria-vesca
FvH4 6g05610.1 Fragaria-vesca
FvH4 6g04730.1 Fragaria-vesca
evm.TU.scaf 30.340 Fragaria-iinumae
FvH4 3g36130.1 Fragaria-vesca
RC5G0555100 Rosa-chinensis
RC5G0555900 Rosa-chinensis
RC5G0568600 Rosa-chinensis
RC3G0124200 Rosa-chinensis
RC3G0124000 Rosa-chinensis

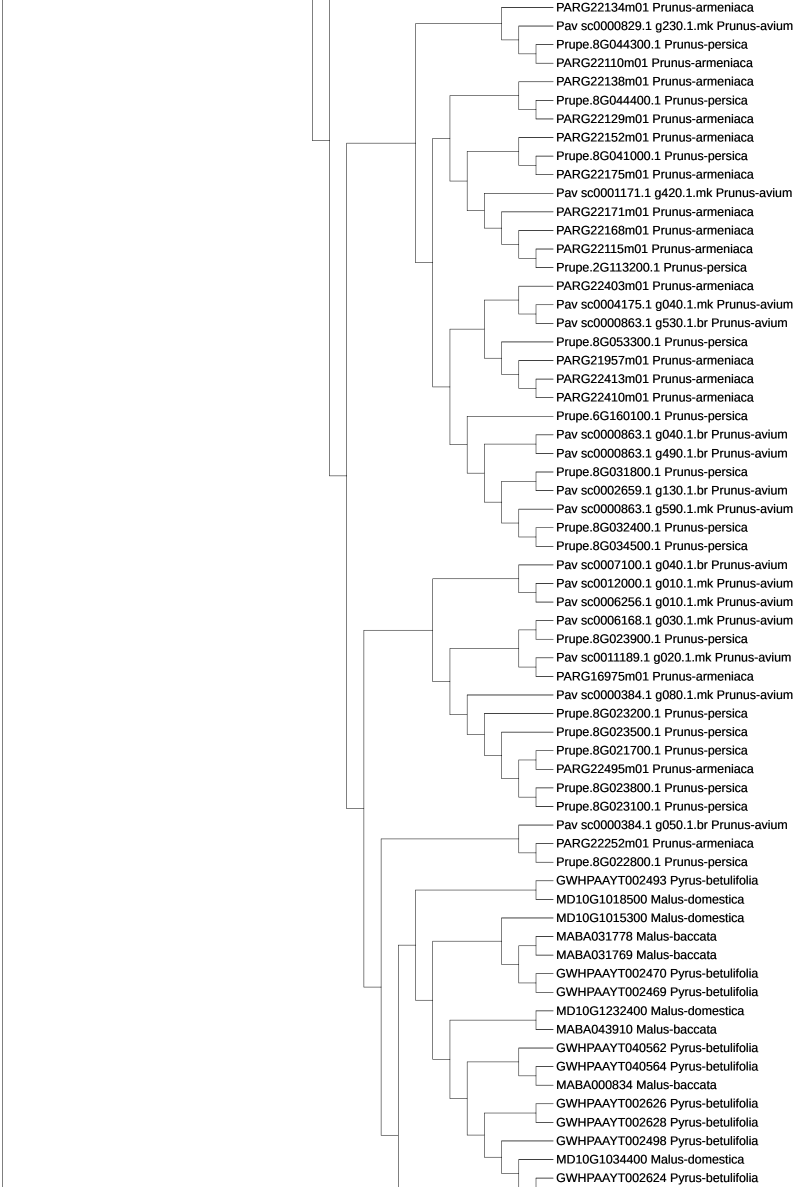
PARG22134m01 Prunus-armeniaca
Pav sc0000829.1 g230.1.mk Prunus-avium
Prupe.8G044300.1 Prunus-persica
PARG22110m01 Prunus-armeniaca
PARG22138m01 Prunus-armeniaca
Prupe.8G044400.1 Prunus-persica
PARG22129m01 Prunus-armeniaca
PARG22152m01 Prunus-armeniaca
Prupe.8G041000.1 Prunus-persica
PARG22175m01 Prunus-armeniaca
Pav sc0001171.1 g420.1.mk Prunus-avium
PARG22171m01 Prunus-armeniaca
PARG22168m01 Prunus-armeniaca
PARG22115m01 Prunus-armeniaca
Prupe.2G113200.1 Prunus-persica
PARG22403m01 Prunus-armeniaca
Pav sc0004175.1 g040.1.mk Prunus-avium
Pav sc0000863.1 g530.1.br Prunus-avium
Prupe.8G053300.1 Prunus-persica
PARG21957m01 Prunus-armeniaca
PARG22413m01 Prunus-armeniaca
PARG22410m01 Prunus-armeniaca
Prupe.6G160100.1 Prunus-persica
Pav sc0000863.1 g040.1.br Prunus-avium
Pav sc0000863.1 g490.1.br Prunus-avium
Prupe.8G031800.1 Prunus-persica
Pav sc0002659.1 g130.1.br Prunus-avium
Pav sc0000863.1 g590.1.mk Prunus-avium
Prupe.8G032400.1 Prunus-persica
Prupe.8G034500.1 Prunus-persica
Pav sc0007100.1 g040.1.br Prunus-avium
Pav sc0012000.1 g010.1.mk Prunus-avium
Pav sc0006256.1 g010.1.mk Prunus-avium
Pav sc0006168.1 g030.1.mk Prunus-avium
Prupe.8G023900.1 Prunus-persica
Pav sc0011189.1 g020.1.mk Prunus-avium
PARG16975m01 Prunus-armeniaca
Pav sc0000384.1 g080.1.mk Prunus-avium
Prupe.8G023200.1 Prunus-persica
Prupe.8G023500.1 Prunus-persica
Prupe.8G021700.1 Prunus-persica
PARG22495m01 Prunus-armeniaca
Prupe.8G023800.1 Prunus-persica
Prupe.8G023100.1 Prunus-persica
Pav sc0000384.1 g050.1.br Prunus-avium
PARG22252m01 Prunus-armeniaca
Prupe.8G022800.1 Prunus-persica
GWHPAAYT002493 Pyrus-betulifolia
MD10G1018500 Malus-domestica
MD10G1015300 Malus-domestica
MABA031778 Malus-baccata
MABA031769 Malus-baccata
GWHPAAYT002470 Pyrus-betulifolia
GWHPAAYT002469 Pyrus-betulifolia
MD10G1232400 Malus-domestica
MABA043910 Malus-baccata
GWHPAAYT040562 Pyrus-betulifolia
GWHPAAYT040564 Pyrus-betulifolia
MABA000834 Malus-baccata
GWHPAAYT002626 Pyrus-betulifolia
GWHPAAYT002628 Pyrus-betulifolia
GWHPAAYT002498 Pyrus-betulifolia
MD10G1034400 Malus-domestica
GWHPAAYT002624 Pyrus-betulifolia

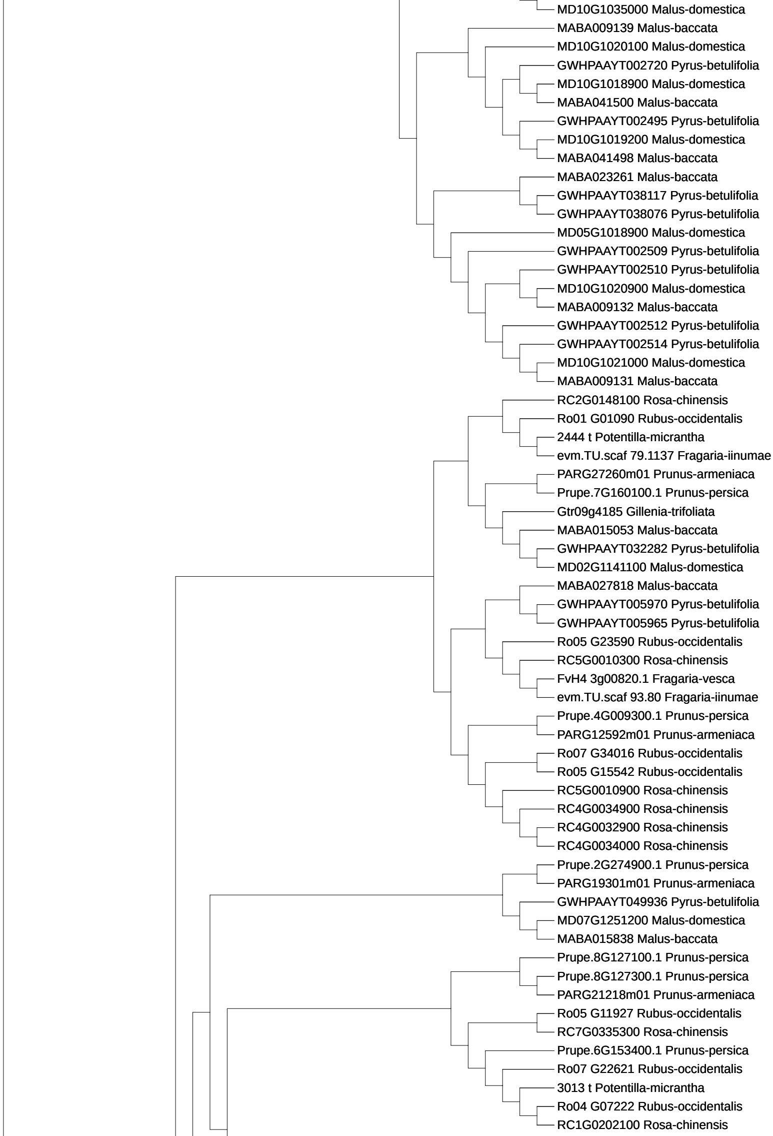
MD10G1035000 Malus-domestica
MABA009139 Malus-baccata
MD10G1020100 Malus-domestica
GWHPAAYT002720 Pyrus-betulifolia
MD10G1018900 Malus-domestica
MABA041500 Malus-baccata
GWHPAAYT002495 Pyrus-betulifolia
MD10G1019200 Malus-domestica
MABA041498 Malus-baccata
MABA023261 Malus-baccata
GWHPAAYT038117 Pyrus-betulifolia
GWHPAAYT038076 Pyrus-betulifolia
MD05G1018900 Malus-domestica
GWHPAAYT002509 Pyrus-betulifolia
GWHPAAYT002510 Pyrus-betulifolia
MD10G1020900 Malus-domestica
MABA009132 Malus-baccata
GWHPAAYT002512 Pyrus-betulifolia
GWHPAAYT002514 Pyrus-betulifolia
MD10G1021000 Malus-domestica
MABA009131 Malus-baccata
RC2G0148100 Rosa-chinensis
Ro01 G01090 Rubus-occidentalis
2444 t Potentilla-micrantha
evm.TU.scaf 79.1137 Fragaria-iinumae
PARG27260m01 Prunus-armeniaca
Prupe.7G160100.1 Prunus-persica
Gtr09g4185 Gillenia-trifoliata
MABA015053 Malus-baccata
GWHPAAYT032282 Pyrus-betulifolia
MD02G1141100 Malus-domestica
MABA027818 Malus-baccata
GWHPAAYT005970 Pyrus-betulifolia
GWHPAAYT005965 Pyrus-betulifolia
Ro05 G23590 Rubus-occidentalis
RC5G0010300 Rosa-chinensis
FvH4 3g00820.1 Fragaria-vesca
evm.TU.scaf 93.80 Fragaria-iinumae
Prupe.4G009300.1 Prunus-persica
PARG12592m01 Prunus-armeniaca
Ro07 G34016 Rubus-occidentalis
Ro05 G15542 Rubus-occidentalis
RC5G0010900 Rosa-chinensis
RC4G0034900 Rosa-chinensis
RC4G0032900 Rosa-chinensis
RC4G0034000 Rosa-chinensis
Prupe.2G274900.1 Prunus-persica
PARG19301m01 Prunus-armeniaca
GWHPAAYT049936 Pyrus-betulifolia
MD07G1251200 Malus-domestica
MABA015838 Malus-baccata
Prupe.8G127100.1 Prunus-persica
Prupe.8G127300.1 Prunus-persica
PARG21218m01 Prunus-armeniaca
Ro05 G11927 Rubus-occidentalis
RC7G0335300 Rosa-chinensis
Prupe.6G153400.1 Prunus-persica
Ro07 G22621 Rubus-occidentalis
3013 t Potentilla-micrantha
Ro04 G07222 Rubus-occidentalis
RC1G0202100 Rosa-chinensis

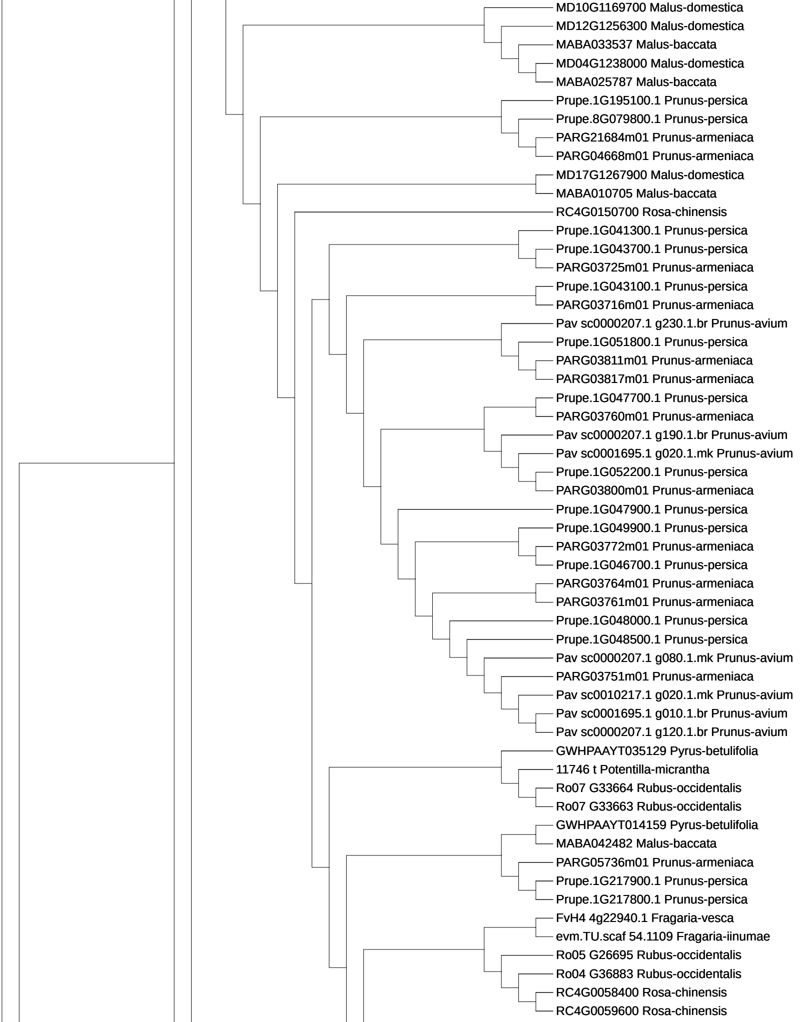
MD10G1169700 Malus-domestica
MD12G1256300 Malus-domestica
MABA033537 Malus-baccata
MD04G1238000 Malus-domestica
MABA025787 Malus-baccata
Prupe.1G195100.1 Prunus-persica
Prupe.8G079800.1 Prunus-persica
PARG21684m01 Prunus-armeniaca
PARG04668m01 Prunus-armeniaca
MD17G1267900 Malus-domestica
MABA010705 Malus-baccata
RC4G0150700 Rosa-chinensis
Prupe.1G041300.1 Prunus-persica
Prupe.1G043700.1 Prunus-persica
PARG03725m01 Prunus-armeniaca
Prupe.1G043100.1 Prunus-persica
PARG03716m01 Prunus-armeniaca
Pav sc0000207.1 g230.1.br Prunus-avium
Prupe.1G051800.1 Prunus-persica
PARG03811m01 Prunus-armeniaca
PARG03817m01 Prunus-armeniaca
Prupe.1G047700.1 Prunus-persica
PARG03760m01 Prunus-armeniaca
Pav sc0000207.1 g190.1.br Prunus-avium
Pav sc0001695.1 g020.1.mk Prunus-avium
Prupe.1G052200.1 Prunus-persica
PARG03800m01 Prunus-armeniaca
Prupe.1G047900.1 Prunus-persica
Prupe.1G049900.1 Prunus-persica
PARG03772m01 Prunus-armeniaca
Prupe.1G046700.1 Prunus-persica
PARG03764m01 Prunus-armeniaca
PARG03761m01 Prunus-armeniaca
Prupe.1G048000.1 Prunus-persica
Prupe.1G048500.1 Prunus-persica
Pav sc0000207.1 g080.1.mk Prunus-avium
PARG03751m01 Prunus-armeniaca
Pav sc0010217.1 g020.1.mk Prunus-avium
Pav sc0001695.1 g010.1.br Prunus-avium
Pav sc0000207.1 g120.1.br Prunus-avium
GWHPAAYT035129 Pyrus-betulifolia
11746 t Potentilla-micrantha
Ro07 G33664 Rubus-occidentalis
Ro07 G33663 Rubus-occidentalis
GWHPAAYT014159 Pyrus-betulifolia
MABA042482 Malus-baccata
PARG05736m01 Prunus-armeniaca
Prupe.1G217900.1 Prunus-persica
Prupe.1G217800.1 Prunus-persica
FvH4 4g22940.1 Fragaria-vesca
evm.TU.scaf 54.1109 Fragaria-iinumae
Ro05 G26695 Rubus-occidentalis
Ro04 G36883 Rubus-occidentalis
RC4G0058400 Rosa-chinensis
RC4G0059600 Rosa-chinensis

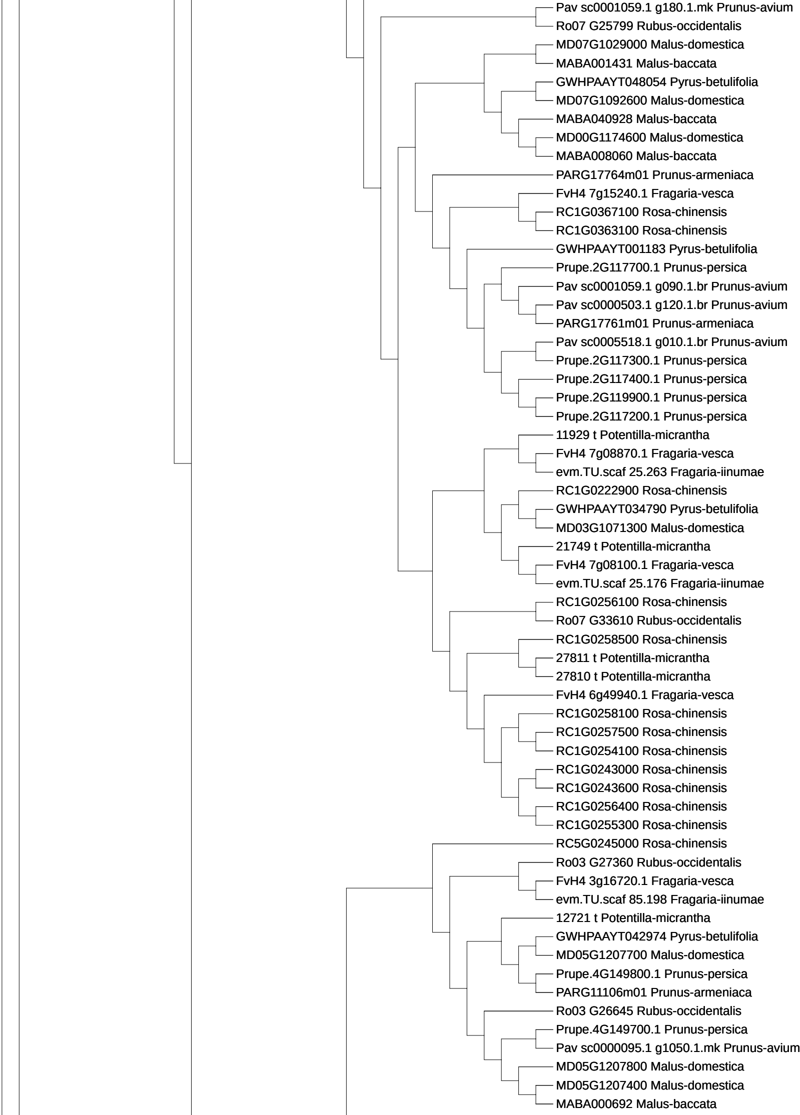
Pav sc0001059.1 g180.1.mk Prunus-avium
Ro07 G25799 Rubus-occidentalis
MD07G1029000 Malus-domestica
MABA001431 Malus-baccata
GWHPAAYT048054 Pyrus-betulifolia
MD07G1092600 Malus-domestica
MABA040928 Malus-baccata
MD00G1174600 Malus-domestica
MABA008060 Malus-baccata
PARG17764m01 Prunus-armeniaca
FvH4 7g15240.1 Fragaria-vesca
RC1G0367100 Rosa-chinensis
RC1G0363100 Rosa-chinensis
GWHPAAYT001183 Pyrus-betulifolia
Prupe.2G117700.1 Prunus-persica
Pav sc0001059.1 g090.1.br Prunus-avium
Pav sc0000503.1 g120.1.br Prunus-avium
PARG17761m01 Prunus-armeniaca
Pav sc0005518.1 g010.1.br Prunus-avium
Prupe.2G117300.1 Prunus-persica
Prupe.2G117400.1 Prunus-persica
Prupe.2G119900.1 Prunus-persica
Prupe.2G117200.1 Prunus-persica
11929 t Potentilla-micrantha
FvH4 7g08870.1 Fragaria-vesca
evm.TU.scaf 25.263 Fragaria-iinumae
RC1G0222900 Rosa-chinensis
GWHPAAYT034790 Pyrus-betulifolia
MD03G1071300 Malus-domestica
21749 t Potentilla-micrantha
FvH4 7g08100.1 Fragaria-vesca
evm.TU.scaf 25.176 Fragaria-iinumae
RC1G0256100 Rosa-chinensis
Ro07 G33610 Rubus-occidentalis
RC1G0258500 Rosa-chinensis
27811 t Potentilla-micrantha
27810 t Potentilla-micrantha
FvH4 6g49940.1 Fragaria-vesca
RC1G0258100 Rosa-chinensis
RC1G0257500 Rosa-chinensis
RC1G0254100 Rosa-chinensis
RC1G0243000 Rosa-chinensis
RC1G0243600 Rosa-chinensis
RC1G0256400 Rosa-chinensis
RC1G0255300 Rosa-chinensis
RC5G0245000 Rosa-chinensis
Ro03 G27360 Rubus-occidentalis
FvH4 3g16720.1 Fragaria-vesca
evm.TU.scaf 85.198 Fragaria-iinumae
12721 t Potentilla-micrantha
GWHPAAYT042974 Pyrus-betulifolia
MD05G1207700 Malus-domestica
Prupe.4G149800.1 Prunus-persica
PARG11106m01 Prunus-armeniaca
Ro03 G26645 Rubus-occidentalis
Prupe.4G149700.1 Prunus-persica
Pav sc0000095.1 g1050.1.mk Prunus-avium
MD05G1207800 Malus-domestica
MD05G1207400 Malus-domestica
MABA000692 Malus-baccata

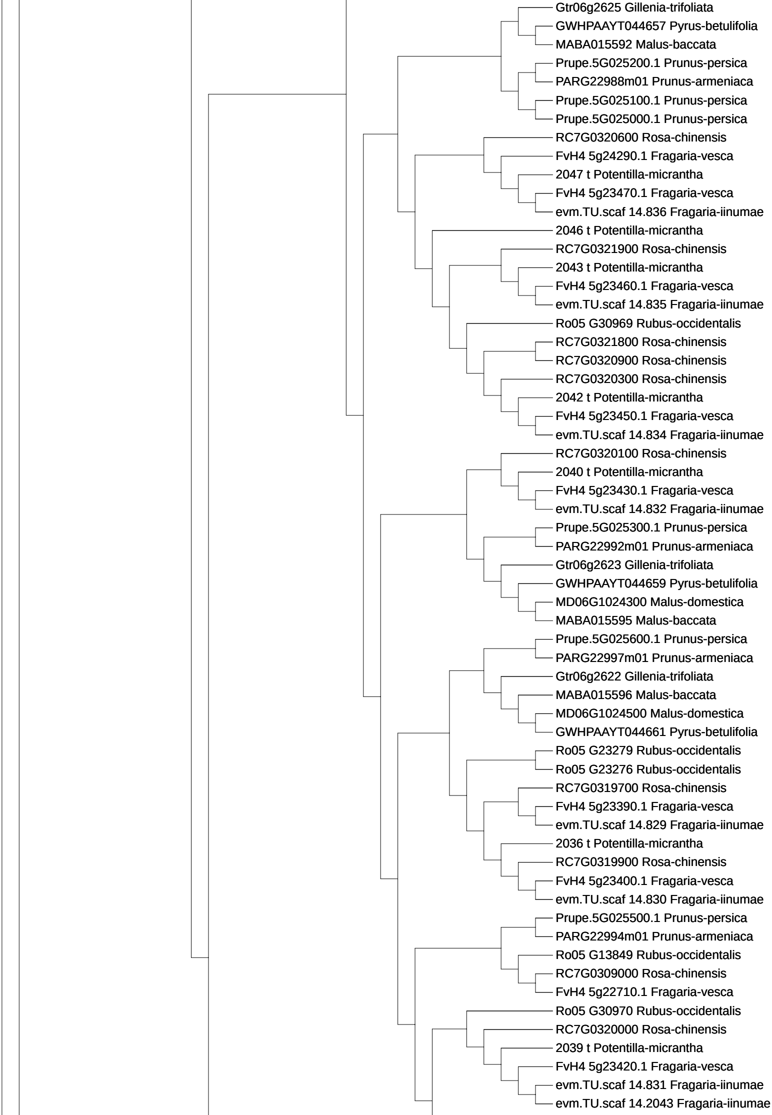
Gtr06g2625 Gillenia-trifoliata
GWHPAAYT044657 Pyrus-betulifolia
MABA015592 Malus-baccata
Prupe.5G025200.1 Prunus-persica
PARG22988m01 Prunus-armeniaca
Prupe.5G025100.1 Prunus-persica
Prupe.5G025000.1 Prunus-persica
RC7G0320600 Rosa-chinensis
FvH4 5g24290.1 Fragaria-vesca
2047 t Potentilla-micrantha
FvH4 5g23470.1 Fragaria-vesca
evm.TU.scaf 14.836 Fragaria-iinumae
2046 t Potentilla-micrantha
RC7G0321900 Rosa-chinensis
2043 t Potentilla-micrantha
FvH4 5g23460.1 Fragaria-vesca
evm.TU.scaf 14.835 Fragaria-iinumae
Ro05 G30969 Rubus-occidentalis
RC7G0321800 Rosa-chinensis
RC7G0320900 Rosa-chinensis
RC7G0320300 Rosa-chinensis
2042 t Potentilla-micrantha
FvH4 5g23450.1 Fragaria-vesca
evm.TU.scaf 14.834 Fragaria-iinumae
RC7G0320100 Rosa-chinensis
2040 t Potentilla-micrantha
FvH4 5g23430.1 Fragaria-vesca
evm.TU.scaf 14.832 Fragaria-iinumae
Prupe.5G025300.1 Prunus-persica
PARG22992m01 Prunus-armeniaca
Gtr06g2623 Gillenia-trifoliata
GWHPAAYT044659 Pyrus-betulifolia
MD06G1024300 Malus-domestica
MABA015595 Malus-baccata
Prupe.5G025600.1 Prunus-persica
PARG22997m01 Prunus-armeniaca
Gtr06g2622 Gillenia-trifoliata
MABA015596 Malus-baccata
MD06G1024500 Malus-domestica
GWHPAAYT044661 Pyrus-betulifolia
Ro05 G23279 Rubus-occidentalis
Ro05 G23276 Rubus-occidentalis
RC7G0319700 Rosa-chinensis
FvH4 5g23390.1 Fragaria-vesca
evm.TU.scaf 14.829 Fragaria-iinumae
2036 t Potentilla-micrantha
RC7G0319900 Rosa-chinensis
FvH4 5g23400.1 Fragaria-vesca
evm.TU.scaf 14.830 Fragaria-iinumae
Prupe.5G025500.1 Prunus-persica
PARG22994m01 Prunus-armeniaca
Ro05 G13849 Rubus-occidentalis
RC7G0309000 Rosa-chinensis
FvH4 5g22710.1 Fragaria-vesca
Ro05 G30970 Rubus-occidentalis
RC7G0320000 Rosa-chinensis
2039 t Potentilla-micrantha
FvH4 5g23420.1 Fragaria-vesca
evm.TU.scaf 14.831 Fragaria-iinumae
evm.TU.scaf 14.2043 Fragaria-iinumae

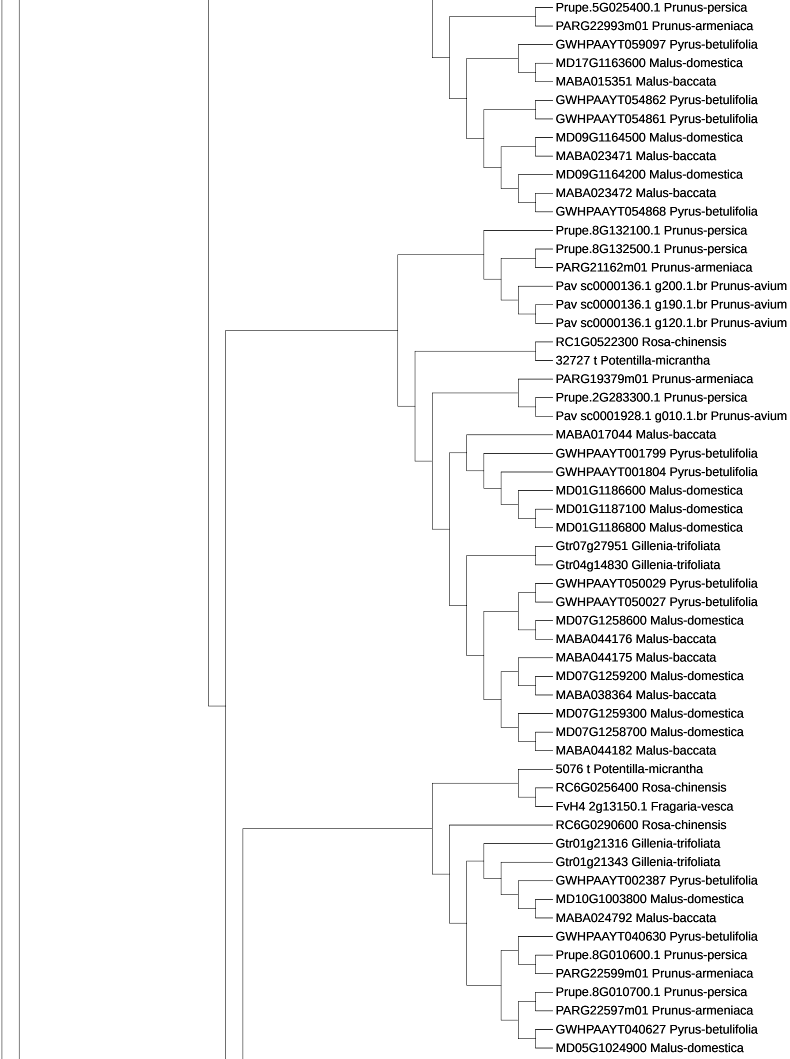
Prupe.5G025400.1 Prunus-persica
PARG22993m01 Prunus-armeniaca
GWHPAAYT059097 Pyrus-betulifolia
MD17G1163600 Malus-domestica
MABA015351 Malus-baccata
GWHPAAYT054862 Pyrus-betulifolia
GWHPAAYT054861 Pyrus-betulifolia
MD09G1164500 Malus-domestica
MABA023471 Malus-baccata
MD09G1164200 Malus-domestica
MABA023472 Malus-baccata
GWHPAAYT054868 Pyrus-betulifolia
Prupe.8G132100.1 Prunus-persica
Prupe.8G132500.1 Prunus-persica
PARG21162m01 Prunus-armeniaca
Pav sc0000136.1 g200.1.br Prunus-avium
Pav sc0000136.1 g190.1.br Prunus-avium
Pav sc0000136.1 g120.1.br Prunus-avium
RC1G0522300 Rosa-chinensis
32727 t Potentilla-micrantha
PARG19379m01 Prunus-armeniaca
Prupe.2G283300.1 Prunus-persica
Pav sc0001928.1 g010.1.br Prunus-avium
MABA017044 Malus-baccata
GWHPAAYT001799 Pyrus-betulifolia
GWHPAAYT001804 Pyrus-betulifolia
MD01G1186600 Malus-domestica
MD01G1187100 Malus-domestica
MD01G1186800 Malus-domestica
Gtr07g27951 Gillenia-trifoliata
Gtr04g14830 Gillenia-trifoliata
GWHPAAYT050029 Pyrus-betulifolia
GWHPAAYT050027 Pyrus-betulifolia
MD07G1258600 Malus-domestica
MABA044176 Malus-baccata
MABA044175 Malus-baccata
MD07G1259200 Malus-domestica
MABA038364 Malus-baccata
MD07G1259300 Malus-domestica
MD07G1258700 Malus-domestica
MABA044182 Malus-baccata
5076 t Potentilla-micrantha
RC6G0256400 Rosa-chinensis
FvH4 2g13150.1 Fragaria-vesca
RC6G0290600 Rosa-chinensis
Gtr01g21316 Gillenia-trifoliata
Gtr01g21343 Gillenia-trifoliata
GWHPAAYT002387 Pyrus-betulifolia
MD10G1003800 Malus-domestica
MABA024792 Malus-baccata
GWHPAAYT040630 Pyrus-betulifolia
Prupe.8G010600.1 Prunus-persica
PARG22599m01 Prunus-armeniaca
Prupe.8G010700.1 Prunus-persica
PARG22597m01 Prunus-armeniaca
GWHPAAYT040627 Pyrus-betulifolia
MD05G1024900 Malus-domestica

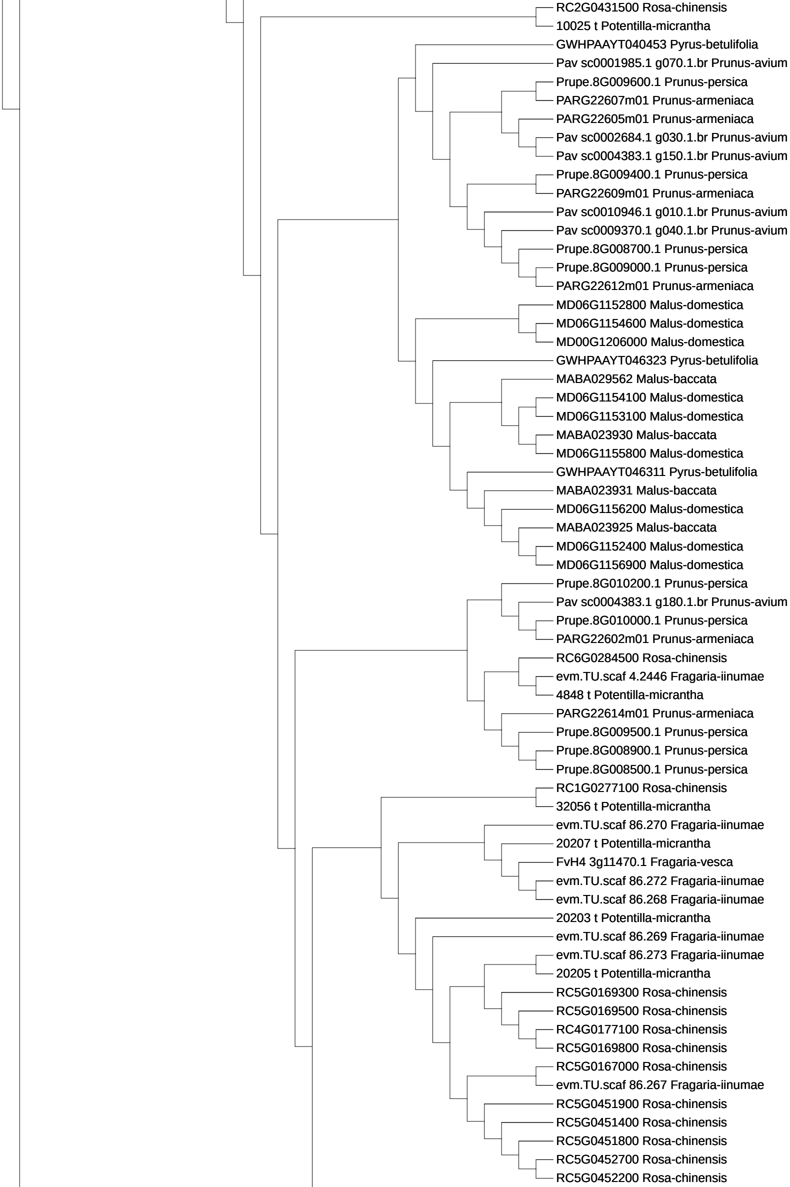
RC2G0431500 Rosa-chinensis
10025 t Potentilla-micrantha
GWHPAAYT040453 Pyrus-betulifolia
Pav sc0001985.1 g070.1.br Prunus-avium
Prupe.8G009600.1 Prunus-persica
PARG22607m01 Prunus-armeniaca
PARG22605m01 Prunus-armeniaca
Pav sc0002684.1 g030.1.br Prunus-avium
Pav sc0004383.1 g150.1.br Prunus-avium
Prupe.8G009400.1 Prunus-persica
PARG22609m01 Prunus-armeniaca
Pav sc0010946.1 g010.1.br Prunus-avium
Pav sc0009370.1 g040.1.br Prunus-avium
Prupe.8G008700.1 Prunus-persica
Prupe.8G009000.1 Prunus-persica
PARG22612m01 Prunus-armeniaca
MD06G1152800 Malus-domestica
MD06G1154600 Malus-domestica
MD00G1206000 Malus-domestica
GWHPAAYT046323 Pyrus-betulifolia
MABA029562 Malus-baccata
MD06G1154100 Malus-domestica
MD06G1153100 Malus-domestica
MABA023930 Malus-baccata
MD06G1155800 Malus-domestica
GWHPAAYT046311 Pyrus-betulifolia
MABA023931 Malus-baccata
MD06G1156200 Malus-domestica
MABA023925 Malus-baccata
MD06G1152400 Malus-domestica
MD06G1156900 Malus-domestica
Prupe.8G010200.1 Prunus-persica
Pav sc0004383.1 g180.1.br Prunus-avium
Prupe.8G010000.1 Prunus-persica
PARG22602m01 Prunus-armeniaca
RC6G0284500 Rosa-chinensis
evm.TU.scaf 4.2446 Fragaria-iinumae
4848 t Potentilla-micrantha
PARG22614m01 Prunus-armeniaca
Prupe.8G009500.1 Prunus-persica
Prupe.8G008900.1 Prunus-persica
Prupe.8G008500.1 Prunus-persica
RC1G0277100 Rosa-chinensis
32056 t Potentilla-micrantha
evm.TU.scaf 86.270 Fragaria-iinumae
20207 t Potentilla-micrantha
FvH4 3g11470.1 Fragaria-vesca
evm.TU.scaf 86.272 Fragaria-iinumae
evm.TU.scaf 86.268 Fragaria-iinumae
20203 t Potentilla-micrantha
evm.TU.scaf 86.269 Fragaria-iinumae
evm.TU.scaf 86.273 Fragaria-iinumae
20205 t Potentilla-micrantha
RC5G0169300 Rosa-chinensis
RC5G0169500 Rosa-chinensis
RC4G0177100 Rosa-chinensis
RC5G0169800 Rosa-chinensis
RC5G0167000 Rosa-chinensis
evm.TU.scaf 86.267 Fragaria-iinumae
RC5G0451900 Rosa-chinensis
RC5G0451400 Rosa-chinensis
RC5G0451800 Rosa-chinensis
RC5G0452700 Rosa-chinensis
RC5G0452200 Rosa-chinensis

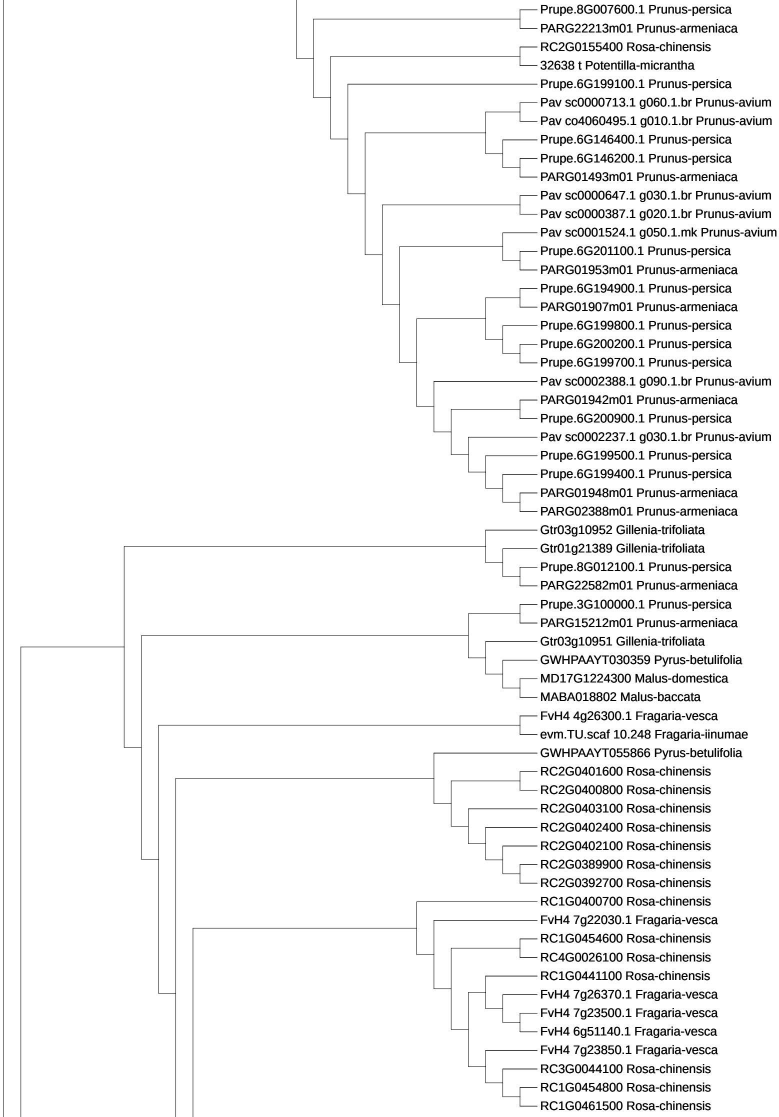
Prupe.8G007600.1 Prunus-persica
PARG22213m01 Prunus-armeniaca
RC2G0155400 Rosa-chinensis
32638 t Potentilla-micrantha
Prupe.6G199100.1 Prunus-persica
Pav sc0000713.1 g060.1.br Prunus-avium
Pav co4060495.1 g010.1.br Prunus-avium
Prupe.6G146400.1 Prunus-persica
Prupe.6G146200.1 Prunus-persica
PARG01493m01 Prunus-armeniaca
Pav sc0000647.1 g030.1.br Prunus-avium
Pav sc0000387.1 g020.1.br Prunus-avium
Pav sc0001524.1 g050.1.mk Prunus-avium
Prupe.6G201100.1 Prunus-persica
PARG01953m01 Prunus-armeniaca
Prupe.6G194900.1 Prunus-persica
PARG01907m01 Prunus-armeniaca
Prupe.6G199800.1 Prunus-persica
Prupe.6G200200.1 Prunus-persica
Prupe.6G199700.1 Prunus-persica
Pav sc0002388.1 g090.1.br Prunus-avium
PARG01942m01 Prunus-armeniaca
Prupe.6G200900.1 Prunus-persica
Pav sc0002237.1 g030.1.br Prunus-avium
Prupe.6G199500.1 Prunus-persica
Prupe.6G199400.1 Prunus-persica
PARG01948m01 Prunus-armeniaca
PARG02388m01 Prunus-armeniaca
Gtr03g10952 Gillenia-trifoliata
Gtr01g21389 Gillenia-trifoliata
Prupe.8G012100.1 Prunus-persica
PARG22582m01 Prunus-armeniaca
Prupe.3G100000.1 Prunus-persica
PARG15212m01 Prunus-armeniaca
Gtr03g10951 Gillenia-trifoliata
GWHPAAYT030359 Pyrus-betulifolia
MD17G1224300 Malus-domestica
MABA018802 Malus-baccata
FvH4 4g26300.1 Fragaria-vesca
evm.TU.scaf 10.248 Fragaria-iinumae
GWHPAAYT055866 Pyrus-betulifolia
RC2G0401600 Rosa-chinensis
RC2G0400800 Rosa-chinensis
RC2G0403100 Rosa-chinensis
RC2G0402400 Rosa-chinensis
RC2G0402100 Rosa-chinensis
RC2G0389900 Rosa-chinensis
RC2G0392700 Rosa-chinensis
RC1G0400700 Rosa-chinensis
FvH4 7g22030.1 Fragaria-vesca
RC1G0454600 Rosa-chinensis
RC4G0026100 Rosa-chinensis
RC1G0441100 Rosa-chinensis
FvH4 7g26370.1 Fragaria-vesca
FvH4 7g23500.1 Fragaria-vesca
FvH4 6g51140.1 Fragaria-vesca
FvH4 7g23850.1 Fragaria-vesca
RC3G0044100 Rosa-chinensis
RC1G0454800 Rosa-chinensis
RC1G0461500 Rosa-chinensis

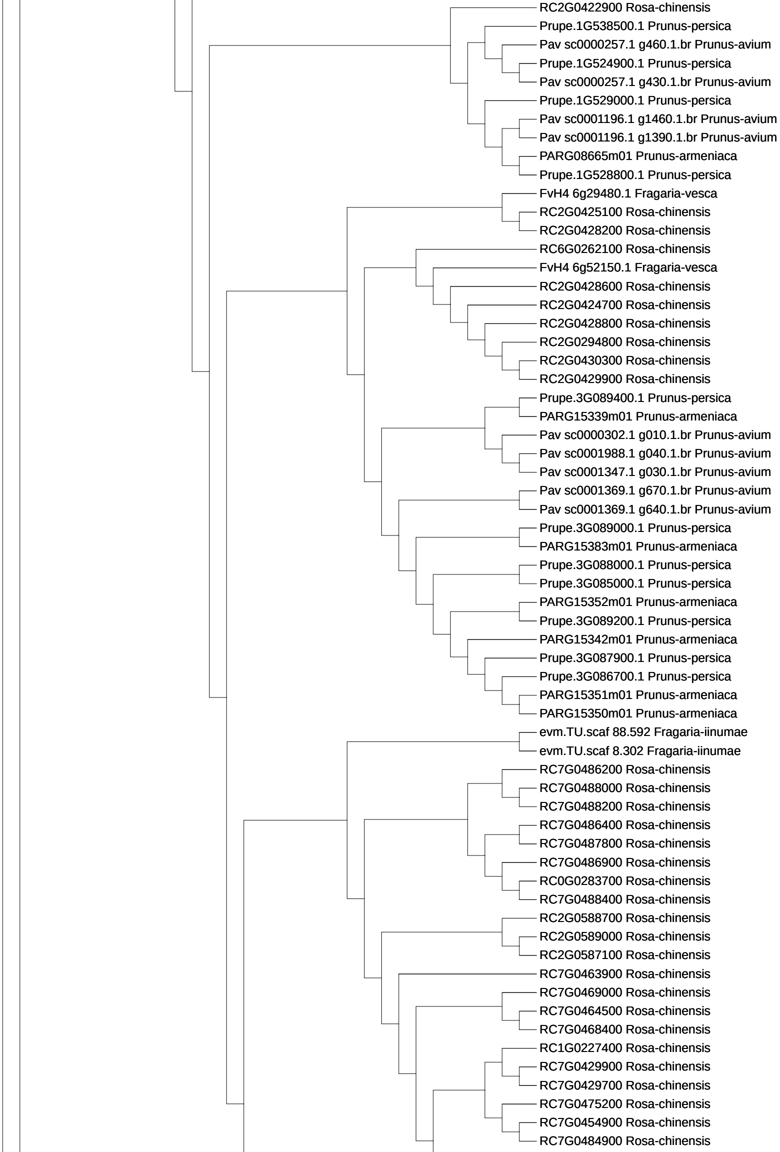
RC2G0422900 Rosa-chinensis
Prupe.1G538500.1 Prunus-persica
Pav sc0000257.1 g460.1.br Prunus-avium
Prupe.1G524900.1 Prunus-persica
Pav sc0000257.1 g430.1.br Prunus-avium
Prupe.1G529000.1 Prunus-persica
Pav sc0001196.1 g1460.1.br Prunus-avium
Pav sc0001196.1 g1390.1.br Prunus-avium
PARG08665m01 Prunus-armeniaca
Prupe.1G528800.1 Prunus-persica
FvH4 6g29480.1 Fragaria-vesca
RC2G0425100 Rosa-chinensis
RC2G0428200 Rosa-chinensis
RC6G0262100 Rosa-chinensis
FvH4 6g52150.1 Fragaria-vesca
RC2G0428600 Rosa-chinensis
RC2G0424700 Rosa-chinensis
RC2G0428800 Rosa-chinensis
RC2G0294800 Rosa-chinensis
RC2G0430300 Rosa-chinensis
RC2G0429900 Rosa-chinensis
Prupe.3G089400.1 Prunus-persica
PARG15339m01 Prunus-armeniaca
Pav sc0000302.1 g010.1.br Prunus-avium
Pav sc0001988.1 g040.1.br Prunus-avium
Pav sc0001347.1 g030.1.br Prunus-avium
Pav sc0001369.1 g670.1.br Prunus-avium
Pav sc0001369.1 g640.1.br Prunus-avium
Prupe.3G089000.1 Prunus-persica
PARG15383m01 Prunus-armeniaca
Prupe.3G088000.1 Prunus-persica
Prupe.3G085000.1 Prunus-persica
PARG15352m01 Prunus-armeniaca
Prupe.3G089200.1 Prunus-persica
PARG15342m01 Prunus-armeniaca
Prupe.3G087900.1 Prunus-persica
Prupe.3G086700.1 Prunus-persica
PARG15351m01 Prunus-armeniaca
PARG15350m01 Prunus-armeniaca
evm.TU.scaf 88.592 Fragaria-iinumae
evm.TU.scaf 8.302 Fragaria-iinumae
RC7G0486200 Rosa-chinensis
RC7G0488000 Rosa-chinensis
RC7G0488200 Rosa-chinensis
RC7G0486400 Rosa-chinensis
RC7G0487800 Rosa-chinensis
RC7G0486900 Rosa-chinensis
RC0G0283700 Rosa-chinensis
RC7G0488400 Rosa-chinensis
RC2G0588700 Rosa-chinensis
RC2G0589000 Rosa-chinensis
RC2G0587100 Rosa-chinensis
RC7G0463900 Rosa-chinensis
RC7G0469000 Rosa-chinensis
RC7G0464500 Rosa-chinensis
RC7G0468400 Rosa-chinensis
RC1G0227400 Rosa-chinensis
RC7G0429900 Rosa-chinensis
RC7G0429700 Rosa-chinensis
RC7G0475200 Rosa-chinensis
RC7G0454900 Rosa-chinensis
RC7G0484900 Rosa-chinensis

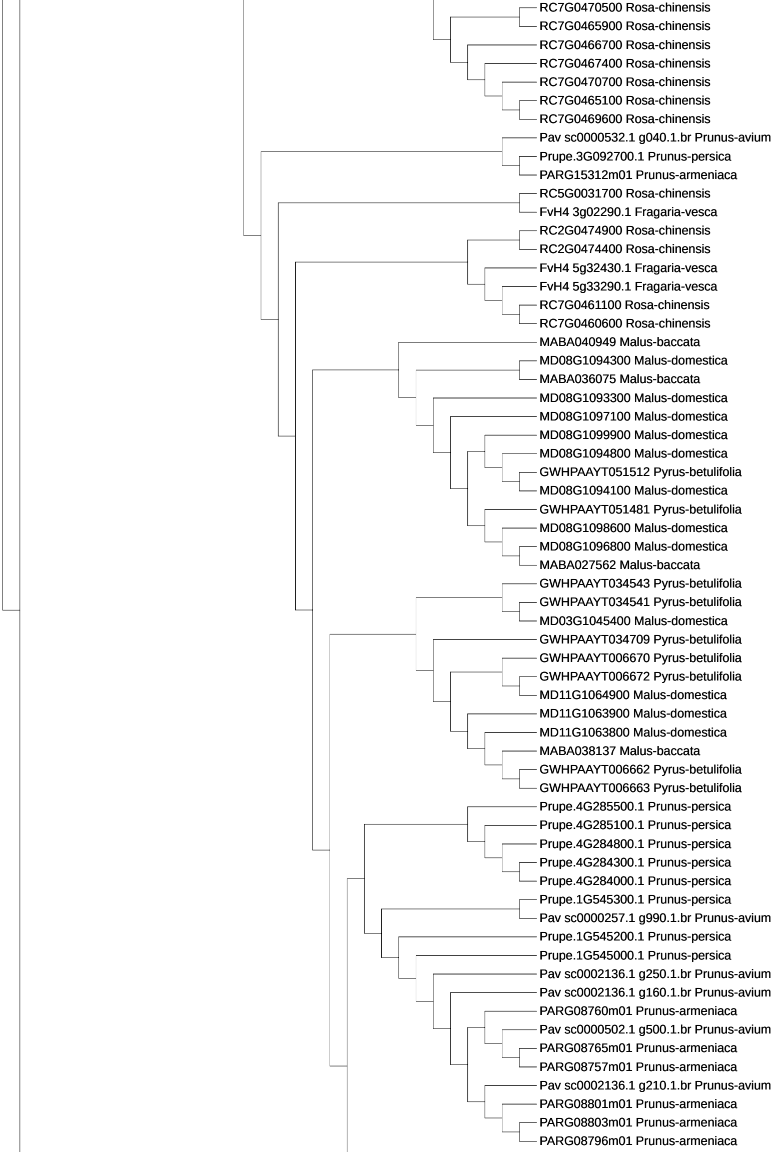
RC7G0470500 Rosa-chinensis
RC7G0465900 Rosa-chinensis
RC7G0466700 Rosa-chinensis
RC7G0467400 Rosa-chinensis
RC7G0470700 Rosa-chinensis
RC7G0465100 Rosa-chinensis
RC7G0469600 Rosa-chinensis
Pav sc0000532.1 g040.1.br Prunus-avium
Prupe.3G092700.1 Prunus-persica
PARG15312m01 Prunus-armeniaca
RC5G0031700 Rosa-chinensis
FvH4 3g02290.1 Fragaria-vesca
RC2G0474900 Rosa-chinensis
RC2G0474400 Rosa-chinensis
FvH4 5g32430.1 Fragaria-vesca
FvH4 5g33290.1 Fragaria-vesca
RC7G0461100 Rosa-chinensis
RC7G0460600 Rosa-chinensis
MABA040949 Malus-baccata
MD08G1094300 Malus-domestica
MABA036075 Malus-baccata
MD08G1093300 Malus-domestica
MD08G1097100 Malus-domestica
MD08G1099900 Malus-domestica
MD08G1094800 Malus-domestica
GWHPAAYT051512 Pyrus-betulifolia
MD08G1094100 Malus-domestica
GWHPAAYT051481 Pyrus-betulifolia
MD08G1098600 Malus-domestica
MD08G1096800 Malus-domestica
MABA027562 Malus-baccata
GWHPAAYT034543 Pyrus-betulifolia
GWHPAAYT034541 Pyrus-betulifolia
MD03G1045400 Malus-domestica
GWHPAAYT034709 Pyrus-betulifolia
GWHPAAYT006670 Pyrus-betulifolia
GWHPAAYT006672 Pyrus-betulifolia
MD11G1064900 Malus-domestica
MD11G1063900 Malus-domestica
MD11G1063800 Malus-domestica
MABA038137 Malus-baccata
GWHPAAYT006662 Pyrus-betulifolia
GWHPAAYT006663 Pyrus-betulifolia
Prupe.4G285500.1 Prunus-persica
Prupe.4G285100.1 Prunus-persica
Prupe.4G284800.1 Prunus-persica
Prupe.4G284300.1 Prunus-persica
Prupe.4G284000.1 Prunus-persica
Prupe.1G545300.1 Prunus-persica
Pav sc0000257.1 g990.1.br Prunus-avium
Prupe.1G545200.1 Prunus-persica
Prupe.1G545000.1 Prunus-persica
Pav sc0002136.1 g250.1.br Prunus-avium
Pav sc0002136.1 g160.1.br Prunus-avium
PARG08760m01 Prunus-armeniaca
Pav sc0000502.1 g500.1.br Prunus-avium
PARG08765m01 Prunus-armeniaca
PARG08757m01 Prunus-armeniaca
Pav sc0002136.1 g210.1.br Prunus-avium
PARG08801m01 Prunus-armeniaca
PARG08803m01 Prunus-armeniaca
PARG08796m01 Prunus-armeniaca

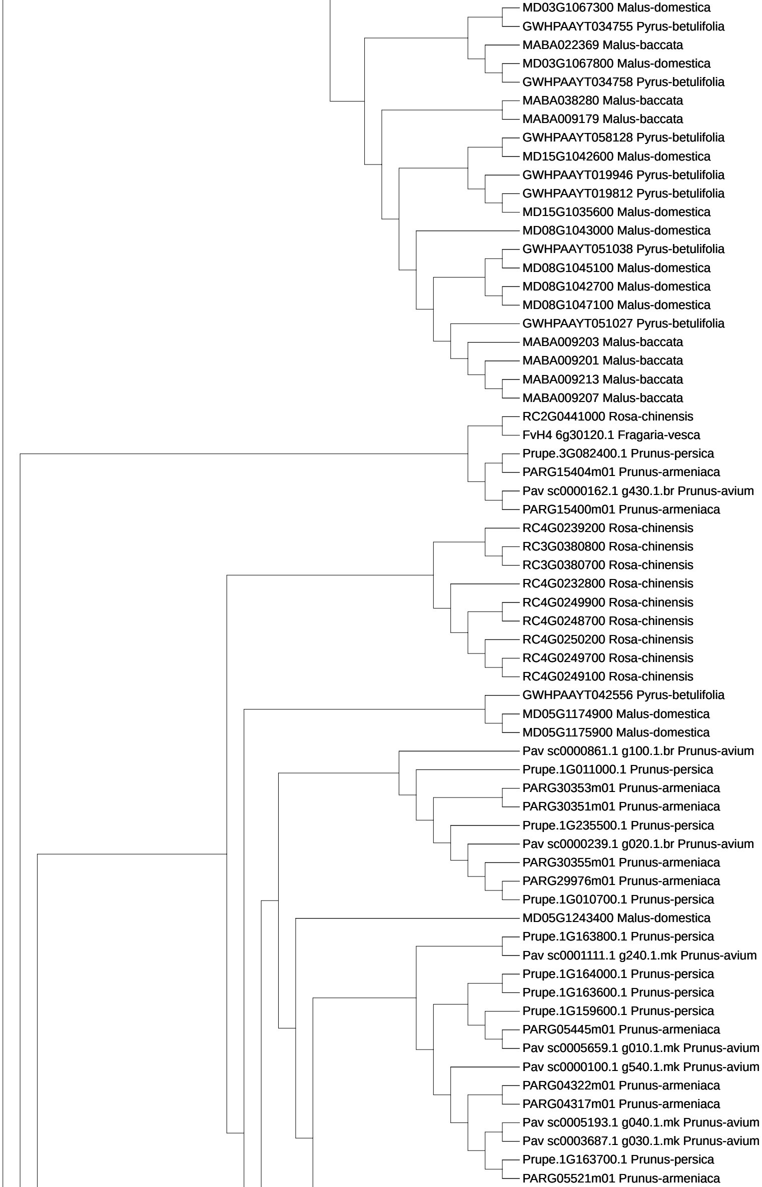
MD03G1067300 Malus-domestica
GWHPAAYT034755 Pyrus-betulifolia
MABA022369 Malus-baccata
MD03G1067800 Malus-domestica
GWHPAAYT034758 Pyrus-betulifolia
MABA038280 Malus-baccata
MABA009179 Malus-baccata
GWHPAAYT058128 Pyrus-betulifolia
MD15G1042600 Malus-domestica
GWHPAAYT019946 Pyrus-betulifolia
GWHPAAYT019812 Pyrus-betulifolia
MD15G1035600 Malus-domestica
MD08G1043000 Malus-domestica
GWHPAAYT051038 Pyrus-betulifolia
MD08G1045100 Malus-domestica
MD08G1042700 Malus-domestica
MD08G1047100 Malus-domestica
GWHPAAYT051027 Pyrus-betulifolia
MABA009203 Malus-baccata
MABA009201 Malus-baccata
MABA009213 Malus-baccata
MABA009207 Malus-baccata
RC2G0441000 Rosa-chinensis
FvH4 6g30120.1 Fragaria-vesca
Prupe.3G082400.1 Prunus-persica
PARG15404m01 Prunus-armeniaca
Pav sc0000162.1 g430.1.br Prunus-avium
PARG15400m01 Prunus-armeniaca
RC4G0239200 Rosa-chinensis
RC3G0380800 Rosa-chinensis
RC3G0380700 Rosa-chinensis
RC4G0232800 Rosa-chinensis
RC4G0249900 Rosa-chinensis
RC4G0248700 Rosa-chinensis
RC4G0250200 Rosa-chinensis
RC4G0249700 Rosa-chinensis
RC4G0249100 Rosa-chinensis
GWHPAAYT042556 Pyrus-betulifolia
MD05G1174900 Malus-domestica
MD05G1175900 Malus-domestica
Pav sc0000861.1 g100.1.br Prunus-avium
Prupe.1G011000.1 Prunus-persica
PARG30353m01 Prunus-armeniaca
PARG30351m01 Prunus-armeniaca
Prupe.1G235500.1 Prunus-persica
Pav sc0000239.1 g020.1.br Prunus-avium
PARG30355m01 Prunus-armeniaca
PARG29976m01 Prunus-armeniaca
Prupe.1G010700.1 Prunus-persica
MD05G1243400 Malus-domestica
Prupe.1G163800.1 Prunus-persica
Pav sc0001111.1 g240.1.mk Prunus-avium
Prupe.1G164000.1 Prunus-persica
Prupe.1G163600.1 Prunus-persica
Prupe.1G159600.1 Prunus-persica
PARG05445m01 Prunus-armeniaca
Pav sc0005659.1 g010.1.mk Prunus-avium
Pav sc0000100.1 g540.1.mk Prunus-avium
PARG04322m01 Prunus-armeniaca
PARG04317m01 Prunus-armeniaca
Pav sc0005193.1 g040.1.mk Prunus-avium
Pav sc0003687.1 g030.1.mk Prunus-avium
Prupe.1G163700.1 Prunus-persica
PARG05521m01 Prunus-armeniaca

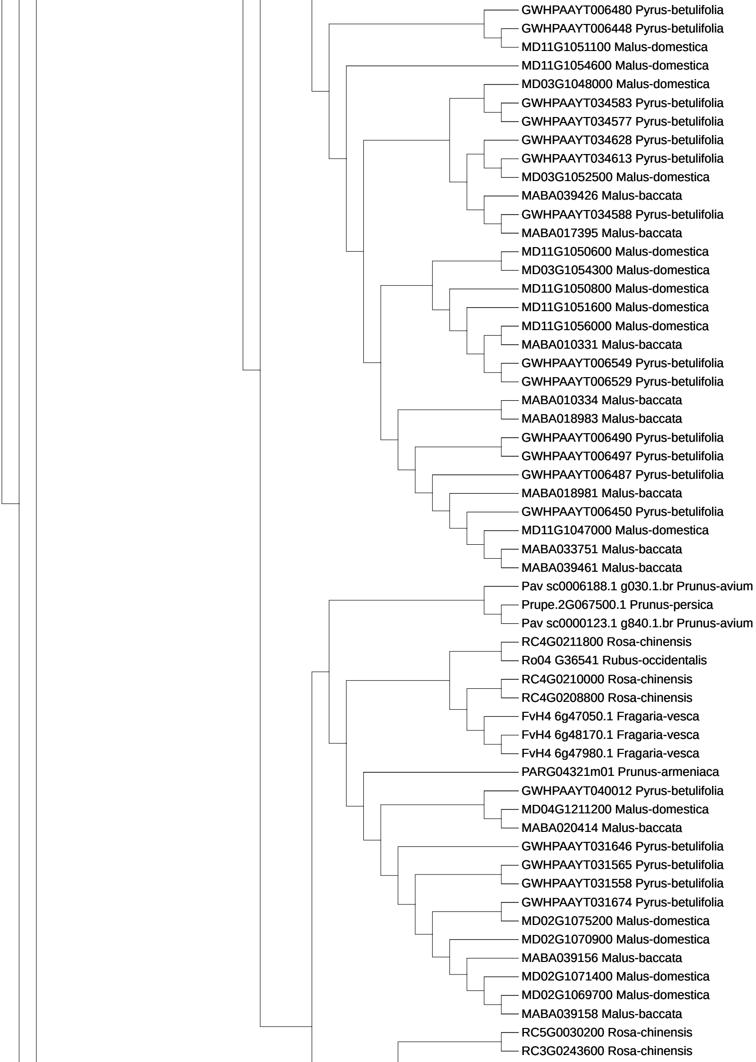
GWHPAAYT006480 Pyrus-betulifolia
GWHPAAYT006448 Pyrus-betulifolia
MD11G1051100 Malus-domestica
MD11G1054600 Malus-domestica
MD03G1048000 Malus-domestica
GWHPAAYT034583 Pyrus-betulifolia
GWHPAAYT034577 Pyrus-betulifolia
GWHPAAYT034628 Pyrus-betulifolia
GWHPAAYT034613 Pyrus-betulifolia
MD03G1052500 Malus-domestica
MABA039426 Malus-baccata
GWHPAAYT034588 Pyrus-betulifolia
MABA017395 Malus-baccata
MD11G1050600 Malus-domestica
MD03G1054300 Malus-domestica
MD11G1050800 Malus-domestica
MD11G1051600 Malus-domestica
MD11G1056000 Malus-domestica
MABA010331 Malus-baccata
GWHPAAYT006549 Pyrus-betulifolia
GWHPAAYT006529 Pyrus-betulifolia
MABA010334 Malus-baccata
MABA018983 Malus-baccata
GWHPAAYT006490 Pyrus-betulifolia
GWHPAAYT006497 Pyrus-betulifolia
GWHPAAYT006487 Pyrus-betulifolia
MABA018981 Malus-baccata
GWHPAAYT006450 Pyrus-betulifolia
MD11G1047000 Malus-domestica
MABA033751 Malus-baccata
MABA039461 Malus-baccata
Pav sc0006188.1 g030.1.br Prunus-avium
Prupe.2G067500.1 Prunus-persica
Pav sc0000123.1 g840.1.br Prunus-avium
RC4G0211800 Rosa-chinensis
Ro04 G36541 Rubus-occidentalis
RC4G0210000 Rosa-chinensis
RC4G0208800 Rosa-chinensis
FvH4 6g47050.1 Fragaria-vesca
FvH4 6g48170.1 Fragaria-vesca
FvH4 6g47980.1 Fragaria-vesca
PARG04321m01 Prunus-armeniaca
GWHPAAYT040012 Pyrus-betulifolia
MD04G1211200 Malus-domestica
MABA020414 Malus-baccata
GWHPAAYT031646 Pyrus-betulifolia
GWHPAAYT031565 Pyrus-betulifolia
GWHPAAYT031558 Pyrus-betulifolia
GWHPAAYT031674 Pyrus-betulifolia
MD02G1075200 Malus-domestica
MD02G1070900 Malus-domestica
MABA039156 Malus-baccata
MD02G1071400 Malus-domestica
MD02G1069700 Malus-domestica
MABA039158 Malus-baccata
RC5G0030200 Rosa-chinensis
RC3G0243600 Rosa-chinensis

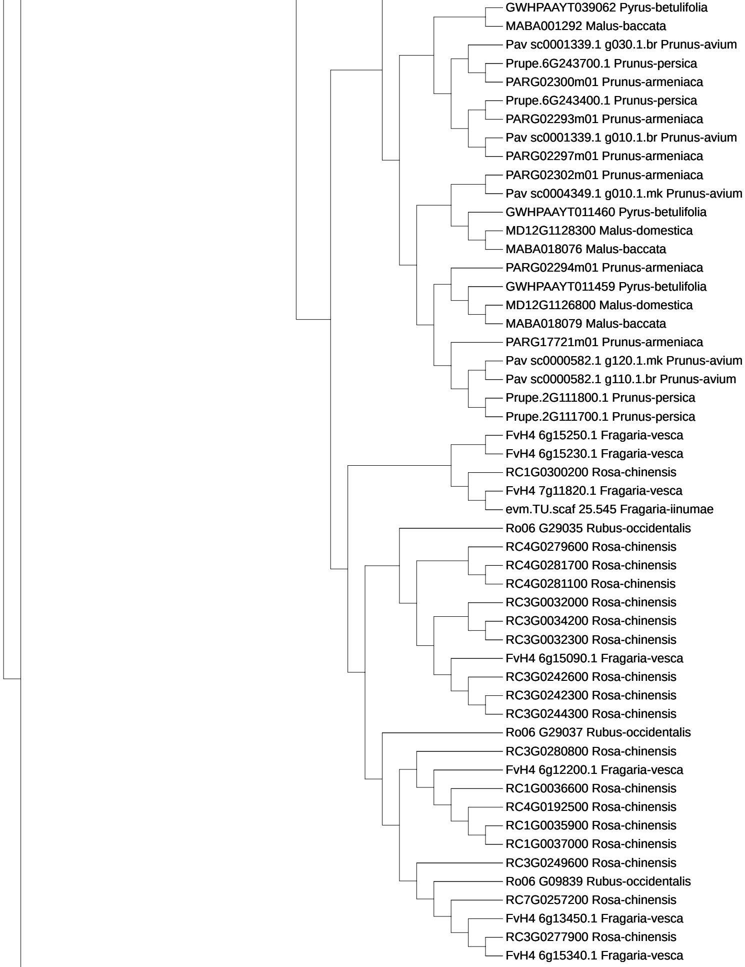
GWHPAAYT039062 Pyrus-betulifolia
MABA001292 Malus-baccata
Pav sc0001339.1 g030.1.br Prunus-avium
Prupe.6G243700.1 Prunus-persica
PARG02300m01 Prunus-armeniaca
Prupe.6G243400.1 Prunus-persica
PARG02293m01 Prunus-armeniaca
Pav sc0001339.1 g010.1.br Prunus-avium
PARG02297m01 Prunus-armeniaca
PARG02302m01 Prunus-armeniaca
Pav sc0004349.1 g010.1.mk Prunus-avium
GWHPAAYT011460 Pyrus-betulifolia
MD12G1128300 Malus-domestica
MABA018076 Malus-baccata
PARG02294m01 Prunus-armeniaca
GWHPAAYT011459 Pyrus-betulifolia
MD12G1126800 Malus-domestica
MABA018079 Malus-baccata
PARG17721m01 Prunus-armeniaca
Pav sc0000582.1 g120.1.mk Prunus-avium
Pav sc0000582.1 g110.1.br Prunus-avium
Prupe.2G111800.1 Prunus-persica
Prupe.2G111700.1 Prunus-persica
FvH4 6g15250.1 Fragaria-vesca
FvH4 6g15230.1 Fragaria-vesca
RC1G0300200 Rosa-chinensis
FvH4 7g11820.1 Fragaria-vesca
evm.TU.scaf 25.545 Fragaria-iinumae
Ro06 G29035 Rubus-occidentalis
RC4G0279600 Rosa-chinensis
RC4G0281700 Rosa-chinensis
RC4G0281100 Rosa-chinensis
RC3G0032000 Rosa-chinensis
RC3G0034200 Rosa-chinensis
RC3G0032300 Rosa-chinensis
FvH4 6g15090.1 Fragaria-vesca
RC3G0242600 Rosa-chinensis
RC3G0242300 Rosa-chinensis
RC3G0244300 Rosa-chinensis
Ro06 G29037 Rubus-occidentalis
RC3G0280800 Rosa-chinensis
FvH4 6g12200.1 Fragaria-vesca
RC1G0036600 Rosa-chinensis
RC4G0192500 Rosa-chinensis
RC1G0035900 Rosa-chinensis
RC1G0037000 Rosa-chinensis
RC3G0249600 Rosa-chinensis
Ro06 G09839 Rubus-occidentalis
RC7G0257200 Rosa-chinensis
FvH4 6g13450.1 Fragaria-vesca
RC3G0277900 Rosa-chinensis
FvH4 6g15340.1 Fragaria-vesca

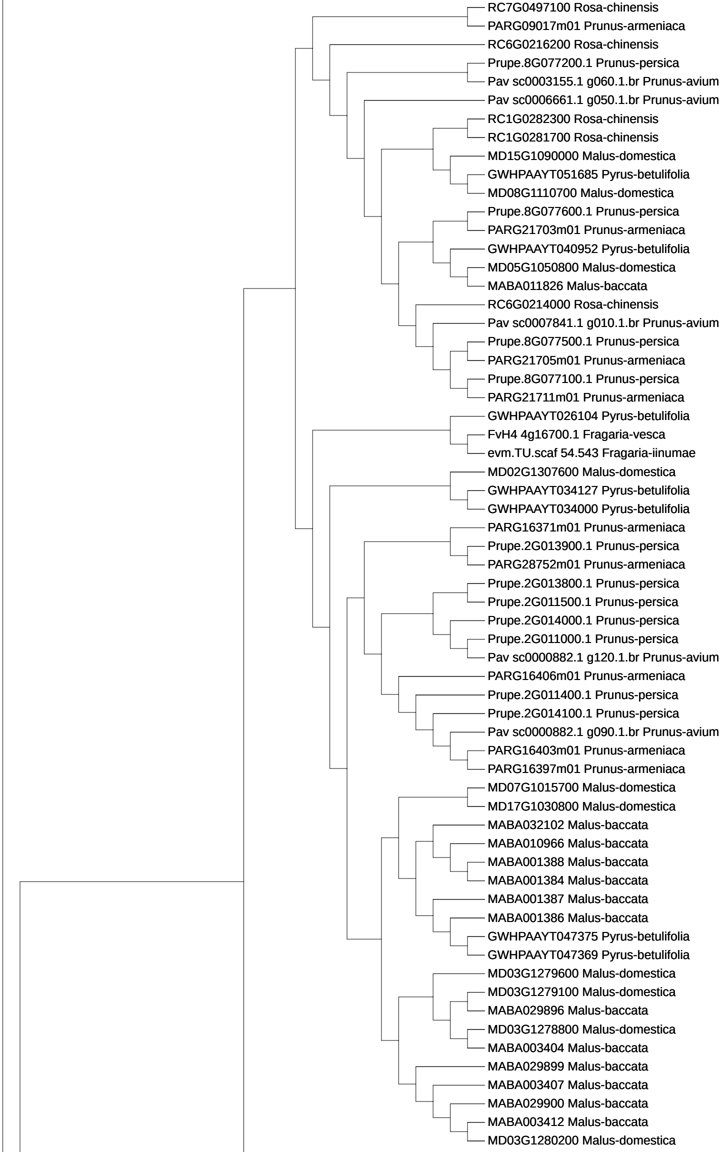
RC7G0497100 Rosa-chinensis
PARG09017m01 Prunus-armeniaca
RC6G0216200 Rosa-chinensis
Prupe.8G077200.1 Prunus-persica
Pav sc0003155.1 g060.1.br Prunus-avium
Pav sc0006661.1 g050.1.br Prunus-avium
RC1G0282300 Rosa-chinensis
RC1G0281700 Rosa-chinensis
MD15G1090000 Malus-domestica
GWHPAAYT051685 Pyrus-betulifolia
MD08G1110700 Malus-domestica
Prupe.8G077600.1 Prunus-persica
PARG21703m01 Prunus-armeniaca
GWHPAAYT040952 Pyrus-betulifolia
MD05G1050800 Malus-domestica
MABA011826 Malus-baccata
RC6G0214000 Rosa-chinensis
Pav sc0007841.1 g010.1.br Prunus-avium
Prupe.8G077500.1 Prunus-persica
PARG21705m01 Prunus-armeniaca
Prupe.8G077100.1 Prunus-persica
PARG21711m01 Prunus-armeniaca
GWHPAAYT026104 Pyrus-betulifolia
FvH4 4g16700.1 Fragaria-vesca
evm.TU.scaf 54.543 Fragaria-iinumae
MD02G1307600 Malus-domestica
GWHPAAYT034127 Pyrus-betulifolia
GWHPAAYT034000 Pyrus-betulifolia
PARG16371m01 Prunus-armeniaca
Prupe.2G013900.1 Prunus-persica
PARG28752m01 Prunus-armeniaca
Prupe.2G013800.1 Prunus-persica
Prupe.2G011500.1 Prunus-persica
Prupe.2G014000.1 Prunus-persica
Prupe.2G011000.1 Prunus-persica
Pav sc0000882.1 g120.1.br Prunus-avium
PARG16406m01 Prunus-armeniaca
Prupe.2G011400.1 Prunus-persica
Prupe.2G014100.1 Prunus-persica
Pav sc0000882.1 g090.1.br Prunus-avium
PARG16403m01 Prunus-armeniaca
PARG16397m01 Prunus-armeniaca
MD07G1015700 Malus-domestica
MD17G1030800 Malus-domestica
MABA032102 Malus-baccata
MABA010966 Malus-baccata
MABA001388 Malus-baccata
MABA001384 Malus-baccata
MABA001387 Malus-baccata
MABA001386 Malus-baccata
GWHPAAYT047375 Pyrus-betulifolia
GWHPAAYT047369 Pyrus-betulifolia
MD03G1279600 Malus-domestica
MD03G1279100 Malus-domestica
MABA029896 Malus-baccata
MD03G1278800 Malus-domestica
MABA003404 Malus-baccata
MABA029899 Malus-baccata
MABA003407 Malus-baccata
MABA029900 Malus-baccata
MABA003412 Malus-baccata
MD03G1280200 Malus-domestica

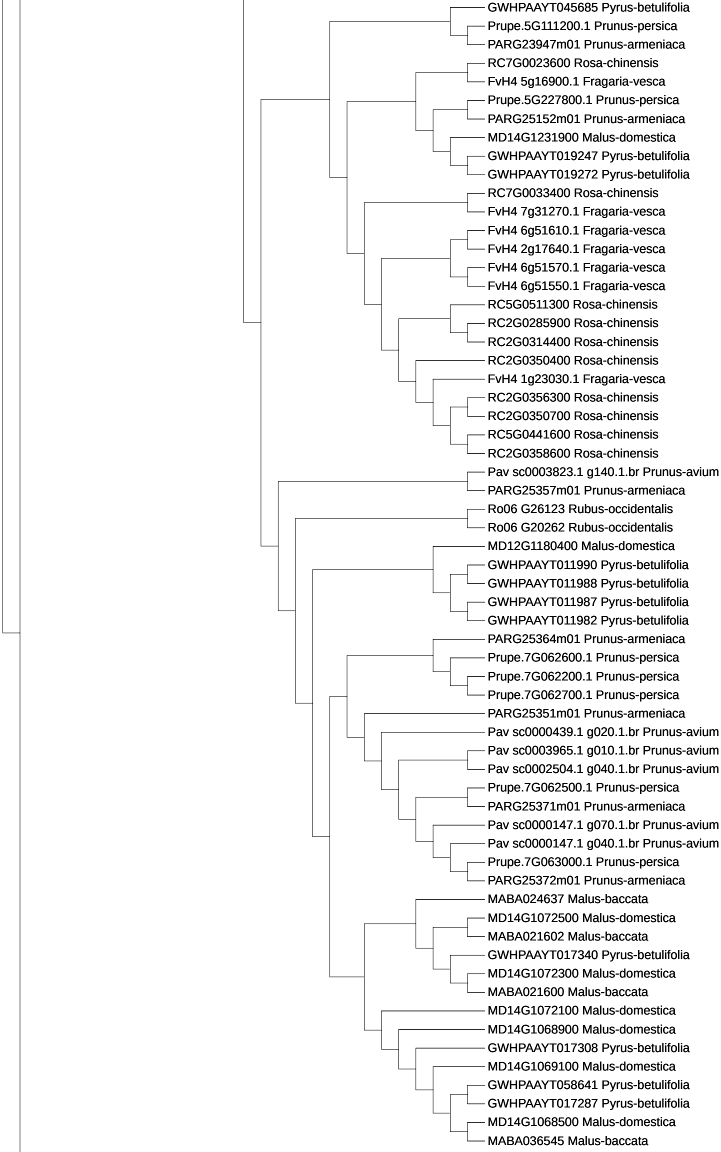

GWHPAAYT045685 Pyrus-betulifolia
Prupe.5G111200.1 Prunus-persica
PARG23947m01 Prunus-armeniaca
RC7G0023600 Rosa-chinensis
FvH4 5g16900.1 Fragaria-vesca
Prupe.5G227800.1 Prunus-persica
PARG25152m01 Prunus-armeniaca
MD14G1231900 Malus-domestica
GWHPAAYT019247 Pyrus-betulifolia
GWHPAAYT019272 Pyrus-betulifolia
RC7G0033400 Rosa-chinensis
FvH4 7g31270.1 Fragaria-vesca
FvH4 6g51610.1 Fragaria-vesca
FvH4 2g17640.1 Fragaria-vesca
FvH4 6g51570.1 Fragaria-vesca
FvH4 6g51550.1 Fragaria-vesca
RC5G0511300 Rosa-chinensis
RC2G0285900 Rosa-chinensis
RC2G0314400 Rosa-chinensis
RC2G0350400 Rosa-chinensis
FvH4 1g23030.1 Fragaria-vesca
RC2G0356300 Rosa-chinensis
RC2G0350700 Rosa-chinensis
RC5G0441600 Rosa-chinensis
RC2G0358600 Rosa-chinensis
Pav sc0003823.1 g140.1.br Prunus-avium
PARG25357m01 Prunus-armeniaca
Ro06 G26123 Rubus-occidentalis
Ro06 G20262 Rubus-occidentalis
MD12G1180400 Malus-domestica
GWHPAAYT011990 Pyrus-betulifolia
GWHPAAYT011988 Pyrus-betulifolia
GWHPAAYT011987 Pyrus-betulifolia
GWHPAAYT011982 Pyrus-betulifolia
PARG25364m01 Prunus-armeniaca
Prupe.7G062600.1 Prunus-persica
Prupe.7G062200.1 Prunus-persica
Prupe.7G062700.1 Prunus-persica
PARG25351m01 Prunus-armeniaca
Pav sc0000439.1 g020.1.br Prunus-avium
Pav sc0003965.1 g010.1.br Prunus-avium
Pav sc0002504.1 g040.1.br Prunus-avium
Prupe.7G062500.1 Prunus-persica
PARG25371m01 Prunus-armeniaca
Pav sc0000147.1 g070.1.br Prunus-avium
Pav sc0000147.1 g040.1.br Prunus-avium
Prupe.7G063000.1 Prunus-persica
PARG25372m01 Prunus-armeniaca
MABA024637 Malus-baccata
MD14G1072500 Malus-domestica
MABA021602 Malus-baccata
GWHPAAYT017340 Pyrus-betulifolia
MD14G1072300 Malus-domestica
MABA021600 Malus-baccata
MD14G1072100 Malus-domestica
MD14G1068900 Malus-domestica
GWHPAAYT017308 Pyrus-betulifolia
MD14G1069100 Malus-domestica
GWHPAAYT058641 Pyrus-betulifolia
GWHPAAYT017287 Pyrus-betulifolia
MD14G1068500 Malus-domestica
MABA036545 Malus-baccata

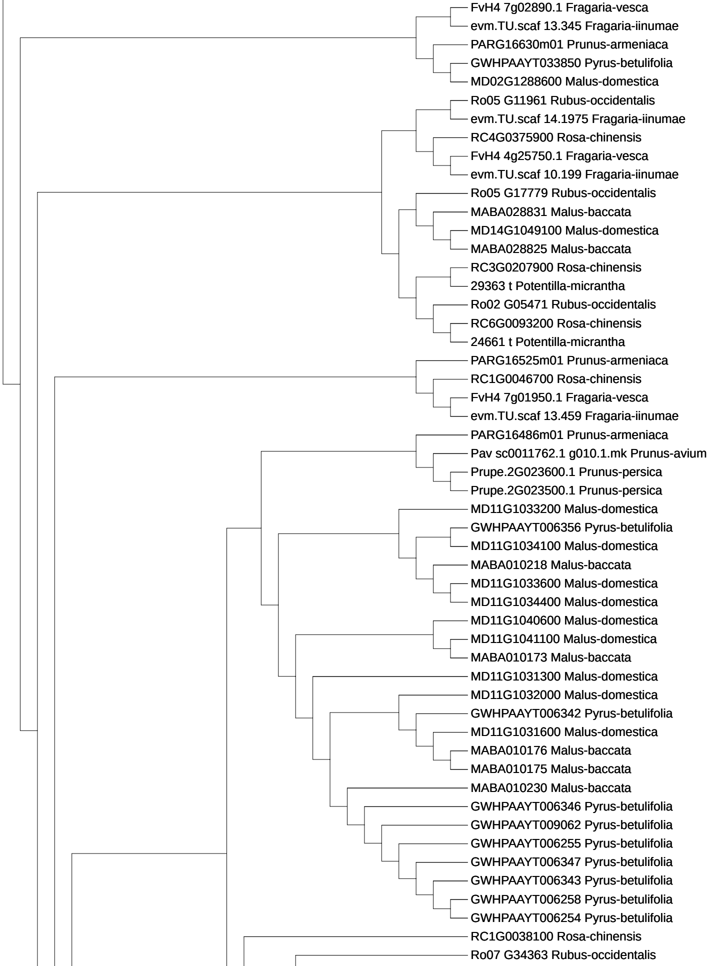
FvH4 7g02890.1 Fragaria-vesca
evm.TU.scaf 13.345 Fragaria-iinumae
PARG16630m01 Prunus-armeniaca
GWHPAAYT033850 Pyrus-betulifolia
MD02G1288600 Malus-domestica
Ro05 G11961 Rubus-occidentalis
evm.TU.scaf 14.1975 Fragaria-iinumae
RC4G0375900 Rosa-chinensis
FvH4 4g25750.1 Fragaria-vesca
evm.TU.scaf 10.199 Fragaria-iinumae
Ro05 G17779 Rubus-occidentalis
MABA028831 Malus-baccata
MD14G1049100 Malus-domestica
MABA028825 Malus-baccata
RC3G0207900 Rosa-chinensis
29363 t Potentilla-micrantha
Ro02 G05471 Rubus-occidentalis
RC6G0093200 Rosa-chinensis
24661 t Potentilla-micrantha
PARG16525m01 Prunus-armeniaca
RC1G0046700 Rosa-chinensis
FvH4 7g01950.1 Fragaria-vesca
evm.TU.scaf 13.459 Fragaria-iinumae
PARG16486m01 Prunus-armeniaca
Pav sc0011762.1 g010.1.mk Prunus-avium
Prupe.2G023600.1 Prunus-persica
Prupe.2G023500.1 Prunus-persica
MD11G1033200 Malus-domestica
GWHPAAYT006356 Pyrus-betulifolia
MD11G1034100 Malus-domestica
MABA010218 Malus-baccata
MD11G1033600 Malus-domestica
MD11G1034400 Malus-domestica
MD11G1040600 Malus-domestica
MD11G1041100 Malus-domestica
MABA010173 Malus-baccata
MD11G1031300 Malus-domestica
MD11G1032000 Malus-domestica
GWHPAAYT006342 Pyrus-betulifolia
MD11G1031600 Malus-domestica
MABA010176 Malus-baccata
MABA010175 Malus-baccata
MABA010230 Malus-baccata
GWHPAAYT006346 Pyrus-betulifolia
GWHPAAYT009062 Pyrus-betulifolia
GWHPAAYT006255 Pyrus-betulifolia
GWHPAAYT006347 Pyrus-betulifolia
GWHPAAYT006343 Pyrus-betulifolia
GWHPAAYT006258 Pyrus-betulifolia
GWHPAAYT006254 Pyrus-betulifolia
RC1G0038100 Rosa-chinensis
Ro07 G34363 Rubus-occidentalis

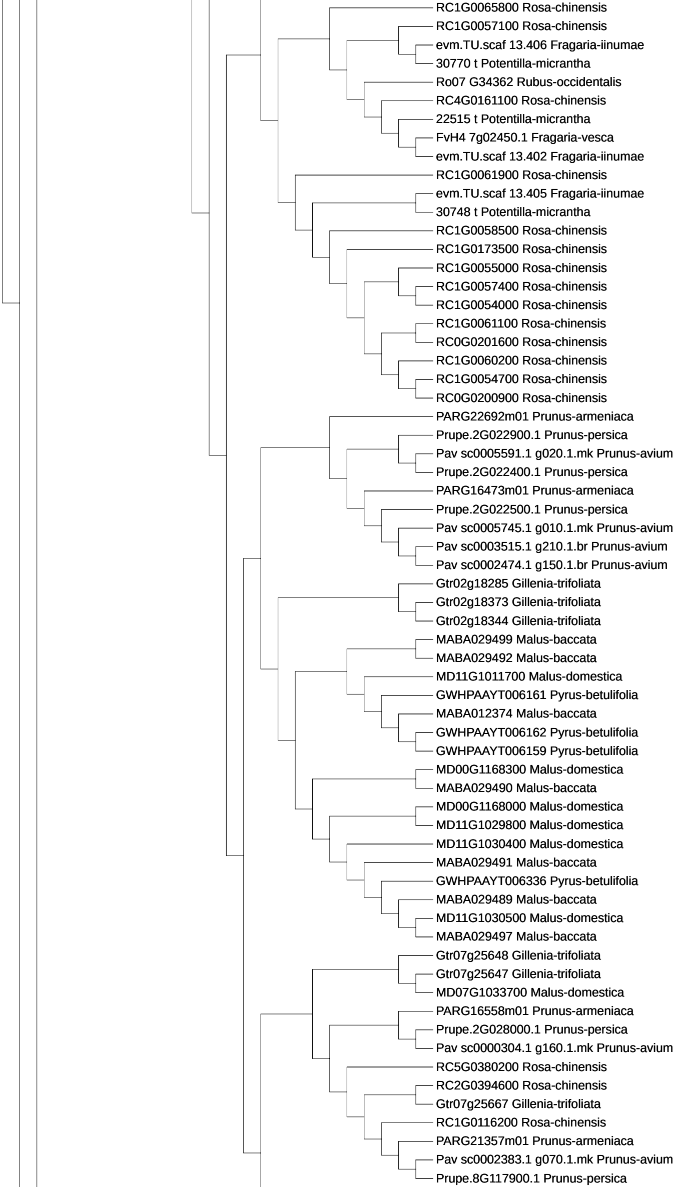
RC1G0065800 Rosa-chinensis
RC1G0057100 Rosa-chinensis
evm.TU.scaf 13.406 Fragaria-iinumae
30770 t Potentilla-micrantha
Ro07 G34362 Rubus-occidentalis
RC4G0161100 Rosa-chinensis
22515 t Potentilla-micrantha
FvH4 7g02450.1 Fragaria-vesca
evm.TU.scaf 13.402 Fragaria-iinumae
RC1G0061900 Rosa-chinensis
evm.TU.scaf 13.405 Fragaria-iinumae
30748 t Potentilla-micrantha
RC1G0058500 Rosa-chinensis
RC1G0173500 Rosa-chinensis
RC1G0055000 Rosa-chinensis
RC1G0057400 Rosa-chinensis
RC1G0054000 Rosa-chinensis
RC1G0061100 Rosa-chinensis
RC0G0201600 Rosa-chinensis
RC1G0060200 Rosa-chinensis
RC1G0054700 Rosa-chinensis
RC0G0200900 Rosa-chinensis
PARG22692m01 Prunus-armeniaca
Prupe.2G022900.1 Prunus-persica
Pav sc0005591.1 g020.1.mk Prunus-avium
Prupe.2G022400.1 Prunus-persica
PARG16473m01 Prunus-armeniaca
Prupe.2G022500.1 Prunus-persica
Pav sc0005745.1 g010.1.mk Prunus-avium
Pav sc0003515.1 g210.1.br Prunus-avium
Pav sc0002474.1 g150.1.br Prunus-avium
Gtr02g18285 Gillenia-trifoliata
Gtr02g18373 Gillenia-trifoliata
Gtr02g18344 Gillenia-trifoliata
MABA029499 Malus-baccata
MABA029492 Malus-baccata
MD11G1011700 Malus-domestica
GWHPAAYT006161 Pyrus-betulifolia
MABA012374 Malus-baccata
GWHPAAYT006162 Pyrus-betulifolia
GWHPAAYT006159 Pyrus-betulifolia
MD00G1168300 Malus-domestica
MABA029490 Malus-baccata
MD00G1168000 Malus-domestica
MD11G1029800 Malus-domestica
MD11G1030400 Malus-domestica
MABA029491 Malus-baccata
GWHPAAYT006336 Pyrus-betulifolia
MABA029489 Malus-baccata
MD11G1030500 Malus-domestica
MABA029497 Malus-baccata
Gtr07g25648 Gillenia-trifoliata
Gtr07g25647 Gillenia-trifoliata
MD07G1033700 Malus-domestica
PARG16558m01 Prunus-armeniaca
Prupe.2G028000.1 Prunus-persica
Pav sc0000304.1 g160.1.mk Prunus-avium
RC5G0380200 Rosa-chinensis
RC2G0394600 Rosa-chinensis
Gtr07g25667 Gillenia-trifoliata
RC1G0116200 Rosa-chinensis
PARG21357m01 Prunus-armeniaca
Pav sc0002383.1 g070.1.mk Prunus-avium
Prupe.8G117900.1 Prunus-persica

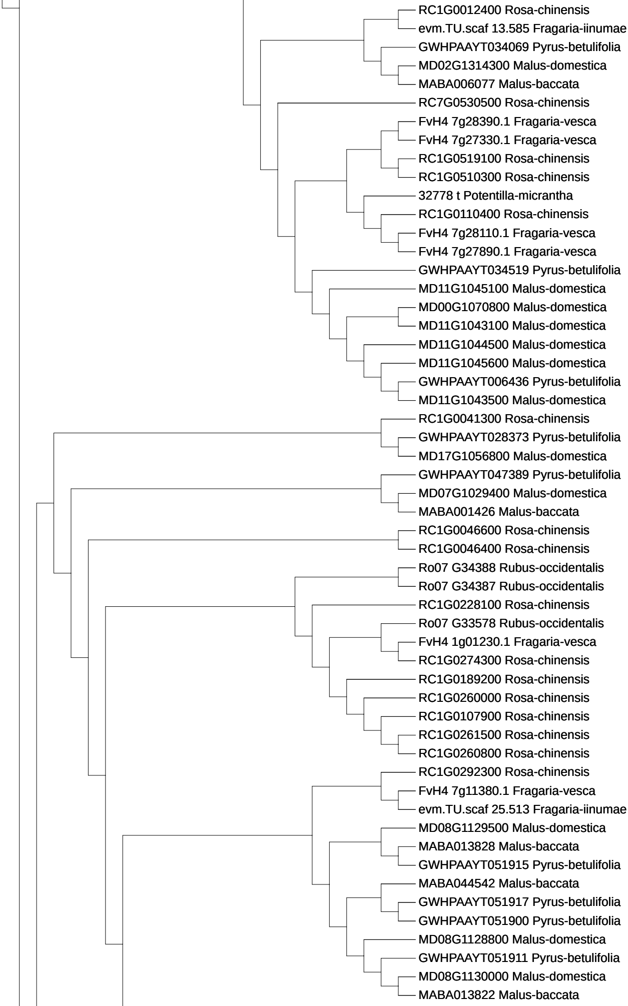
RC1G0012400 Rosa-chinensis
evm.TU.scaf 13.585 Fragaria-iinumae
GWHPAAYT034069 Pyrus-betulifolia
MD02G1314300 Malus-domestica
MABA006077 Malus-baccata
RC7G0530500 Rosa-chinensis
FvH4 7g28390.1 Fragaria-vesca
FvH4 7g27330.1 Fragaria-vesca
RC1G0519100 Rosa-chinensis
RC1G0510300 Rosa-chinensis
32778 t Potentilla-micrantha
RC1G0110400 Rosa-chinensis
FvH4 7g28110.1 Fragaria-vesca
FvH4 7g27890.1 Fragaria-vesca
GWHPAAYT034519 Pyrus-betulifolia
MD11G1045100 Malus-domestica
MD00G1070800 Malus-domestica
MD11G1043100 Malus-domestica
MD11G1044500 Malus-domestica
MD11G1045600 Malus-domestica
GWHPAAYT006436 Pyrus-betulifolia
MD11G1043500 Malus-domestica
RC1G0041300 Rosa-chinensis
GWHPAAYT028373 Pyrus-betulifolia
MD17G1056800 Malus-domestica
GWHPAAYT047389 Pyrus-betulifolia
MD07G1029400 Malus-domestica
MABA001426 Malus-baccata
RC1G0046600 Rosa-chinensis
RC1G0046400 Rosa-chinensis
Ro07 G34388 Rubus-occidentalis
Ro07 G34387 Rubus-occidentalis
RC1G0228100 Rosa-chinensis
Ro07 G33578 Rubus-occidentalis
FvH4 1g01230.1 Fragaria-vesca
RC1G0274300 Rosa-chinensis
RC1G0189200 Rosa-chinensis
RC1G0260000 Rosa-chinensis
RC1G0107900 Rosa-chinensis
RC1G0261500 Rosa-chinensis
RC1G0260800 Rosa-chinensis
RC1G0292300 Rosa-chinensis
FvH4 7g11380.1 Fragaria-vesca
evm.TU.scaf 25.513 Fragaria-iinumae
MD08G1129500 Malus-domestica
MABA013828 Malus-baccata
GWHPAAYT051915 Pyrus-betulifolia
MABA044542 Malus-baccata
GWHPAAYT051917 Pyrus-betulifolia
GWHPAAYT051900 Pyrus-betulifolia
MD08G1128800 Malus-domestica
GWHPAAYT051911 Pyrus-betulifolia
MD08G1130000 Malus-domestica
MABA013822 Malus-baccata

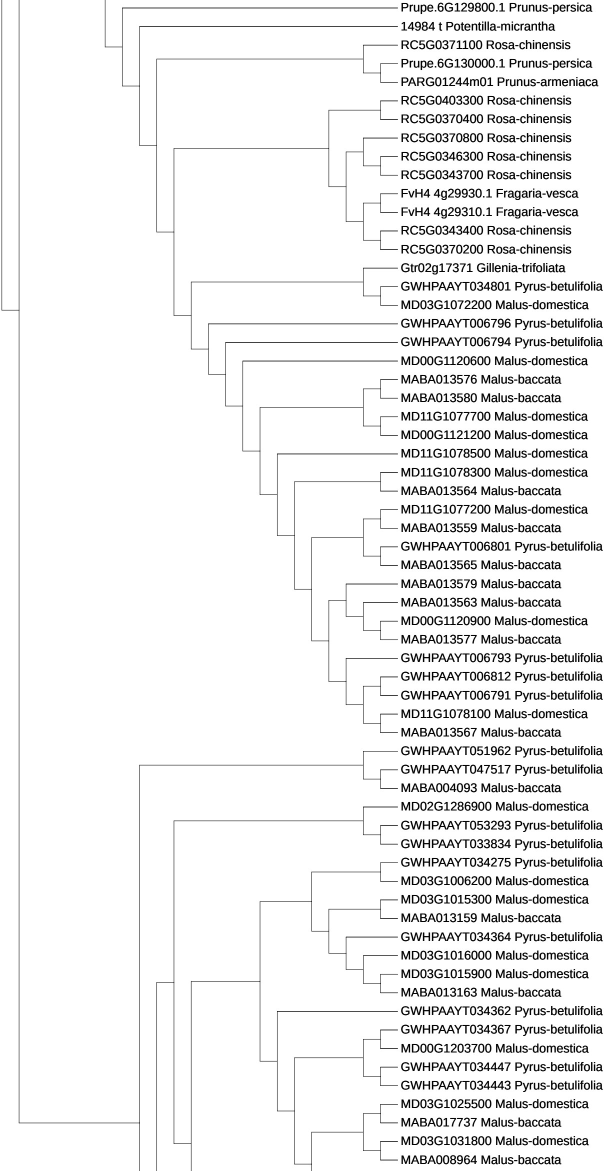
Prupe.6G129800.1 Prunus-persica
14984 t Potentilla-micrantha
RC5G0371100 Rosa-chinensis
Prupe.6G130000.1 Prunus-persica
PARG01244m01 Prunus-armeniaca
RC5G0403300 Rosa-chinensis
RC5G0370400 Rosa-chinensis
RC5G0370800 Rosa-chinensis
RC5G0346300 Rosa-chinensis
RC5G0343700 Rosa-chinensis
FvH4 4g29930.1 Fragaria-vesca
FvH4 4g29310.1 Fragaria-vesca
RC5G0343400 Rosa-chinensis
RC5G0370200 Rosa-chinensis
Gtr02g17371 Gillenia-trifoliata
GWHPAAYT034801 Pyrus-betulifolia
MD03G1072200 Malus-domestica
GWHPAAYT006796 Pyrus-betulifolia
GWHPAAYT006794 Pyrus-betulifolia
MD00G1120600 Malus-domestica
MABA013576 Malus-baccata
MABA013580 Malus-baccata
MD11G1077700 Malus-domestica
MD00G1121200 Malus-domestica
MD11G1078500 Malus-domestica
MD11G1078300 Malus-domestica
MABA013564 Malus-baccata
MD11G1077200 Malus-domestica
MABA013559 Malus-baccata
GWHPAAYT006801 Pyrus-betulifolia
MABA013565 Malus-baccata
MABA013579 Malus-baccata
MABA013563 Malus-baccata
MD00G1120900 Malus-domestica
MABA013577 Malus-baccata
GWHPAAYT006793 Pyrus-betulifolia
GWHPAAYT006812 Pyrus-betulifolia
GWHPAAYT006791 Pyrus-betulifolia
MD11G1078100 Malus-domestica
MABA013567 Malus-baccata
GWHPAAYT051962 Pyrus-betulifolia
GWHPAAYT047517 Pyrus-betulifolia
MABA004093 Malus-baccata
MD02G1286900 Malus-domestica
GWHPAAYT053293 Pyrus-betulifolia
GWHPAAYT033834 Pyrus-betulifolia
GWHPAAYT034275 Pyrus-betulifolia
MD03G1006200 Malus-domestica
MD03G1015300 Malus-domestica
MABA013159 Malus-baccata
GWHPAAYT034364 Pyrus-betulifolia
MD03G1016000 Malus-domestica
MD03G1015900 Malus-domestica
MABA013163 Malus-baccata
GWHPAAYT034362 Pyrus-betulifolia
GWHPAAYT034367 Pyrus-betulifolia
MD00G1203700 Malus-domestica
GWHPAAYT034447 Pyrus-betulifolia
GWHPAAYT034443 Pyrus-betulifolia
MD03G1025500 Malus-domestica
MABA017737 Malus-baccata
MD03G1031800 Malus-domestica
MABA008964 Malus-baccata

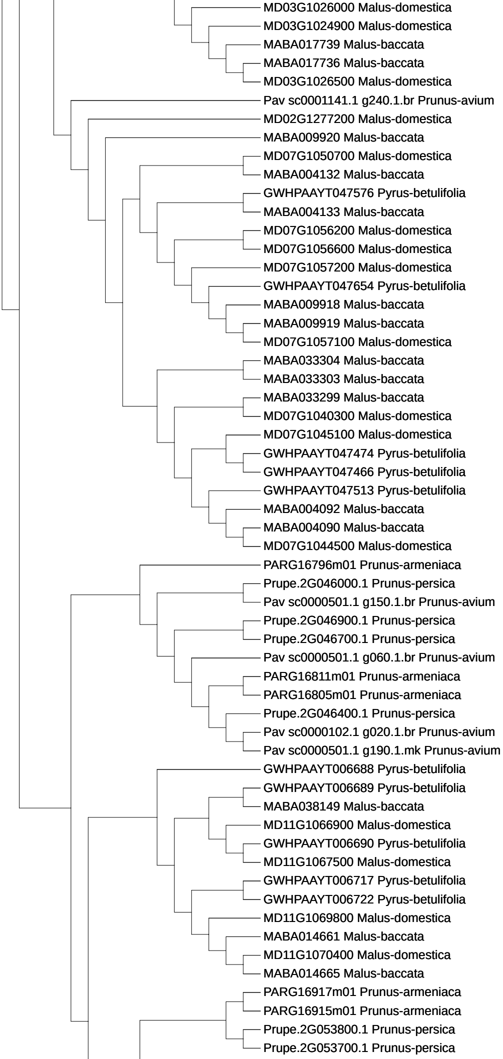
MD03G1026000 Malus-domestica
MD03G1024900 Malus-domestica
MABA017739 Malus-baccata
MABA017736 Malus-baccata
MD03G1026500 Malus-domestica
Pav sc0001141.1 g240.1.br Prunus-avium
MD02G1277200 Malus-domestica
MABA009920 Malus-baccata
MD07G1050700 Malus-domestica
MABA004132 Malus-baccata
GWHPAAYT047576 Pyrus-betulifolia
MABA004133 Malus-baccata
MD07G1056200 Malus-domestica
MD07G1056600 Malus-domestica
MD07G1057200 Malus-domestica
GWHPAAYT047654 Pyrus-betulifolia
MABA009918 Malus-baccata
MABA009919 Malus-baccata
MD07G1057100 Malus-domestica
MABA033304 Malus-baccata
MABA033303 Malus-baccata
MABA033299 Malus-baccata
MD07G1040300 Malus-domestica
MD07G1045100 Malus-domestica
GWHPAAYT047474 Pyrus-betulifolia
GWHPAAYT047466 Pyrus-betulifolia
GWHPAAYT047513 Pyrus-betulifolia
MABA004092 Malus-baccata
MABA004090 Malus-baccata
MD07G1044500 Malus-domestica
PARG16796m01 Prunus-armeniaca
Prupe.2G046000.1 Prunus-persica
Pav sc0000501.1 g150.1.br Prunus-avium
Prupe.2G046900.1 Prunus-persica
Prupe.2G046700.1 Prunus-persica
Pav sc0000501.1 g060.1.br Prunus-avium
PARG16811m01 Prunus-armeniaca
PARG16805m01 Prunus-armeniaca
Prupe.2G046400.1 Prunus-persica
Pav sc0000102.1 g020.1.br Prunus-avium
Pav sc0000501.1 g190.1.mk Prunus-avium
GWHPAAYT006688 Pyrus-betulifolia
GWHPAAYT006689 Pyrus-betulifolia
MABA038149 Malus-baccata
MD11G1066900 Malus-domestica
GWHPAAYT006690 Pyrus-betulifolia
MD11G1067500 Malus-domestica
GWHPAAYT006717 Pyrus-betulifolia
GWHPAAYT006722 Pyrus-betulifolia
MD11G1069800 Malus-domestica
MABA014661 Malus-baccata
MD11G1070400 Malus-domestica
MABA014665 Malus-baccata
PARG16917m01 Prunus-armeniaca
PARG16915m01 Prunus-armeniaca
Prupe.2G053800.1 Prunus-persica
Prupe.2G053700.1 Prunus-persica

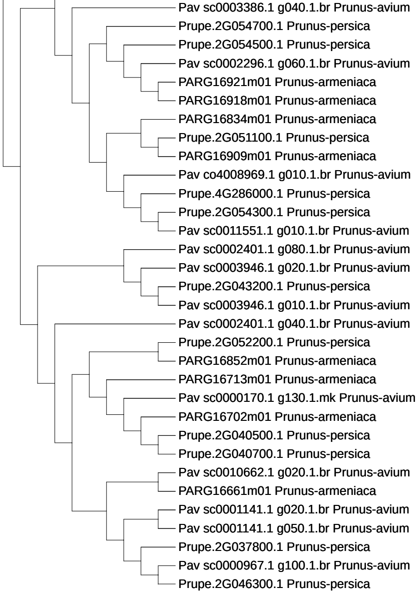

Figure S1 | A detailed phylogenetic tree with all sequence names. The tree was reconstructed based on the NBS domain sequences of the *NBS-LRR* genes from the 12 Rosaceae genomes
